# A Kampo medicine, kamishoyosan, ameliorates menstruation-associated symptoms in patients with premenstrual syndrome: An exploratory open-label, randomized controlled trial

**DOI:** 10.1371/journal.pone.0362303

**Published:** 2026-11-06

**Authors:** Jun Chikumi, Eri Sato, Ai Ikebuchi, Yohei Tokita, Mitsuto Fukushige, Mayu Yamamoto, Hiroshi Sunada, Yusuke Endo, Hisashi Noma, Tasuku Harada, Fuminori Taniguchi

**Affiliations:** 1 Tottori University Faculty of Medicine, Obstetrics and Gynecology, Tottori, Japan; 2 Tottori University Faculty of Medicine, Health Service Center, Tottori, Japan; 3 TSUMURA Kampo Research Laboratories, Research & Development Division, TSUMURA & CO., Tokyo, Japan; 4 Advanced Medicine, Innovation and Clinical Research Center, Tottori University Hospital, Tottori, Japan; 5 Organization for Research Institute and Promotion, Tottori University, Tottori, Japan; 6 Department of Data Science, The Institute of Statistical Mathematics, Tokyo, Japan; Kwame Nkrumah University of Science and Technology Faculty of Pharmacy and Pharmaceutical Sciences, GHANA

## Abstract

Premenstrual syndrome (PMS) is a psychological or physical symptom. We conducted an open-label, randomized, exploratory trial with the aim of evaluating the efficacy and safety of kamishoyosan (KSS) for PMS. KSS was administered orally during three menstrual cycles. The primary endpoint was the change in the premenstrual Menstrual Distress Questionnaire (MDQ) total score. The secondary endpoints included MDQ total and subscale scores, a daily symptom questionnaire, the Work Productivity and Activity Impairment (WPAI) Questionnaire, overall impressions, sleep status, and heart rate. A total of 50 patients were enrolled. The primary endpoint, the premenstrual MDQ total score, was significantly ameliorated after KSS treatment. Furthermore, KSS reduced MDQ scores during the menstrual cycle. Among the MDQ subscales, KSS decreased score of pain, concentration, behavioral change, and negative feelings during the premenstrual cycle. In addition, hyperthymia reduced during the menstrual cycle. The results of the daily symptom questionnaire indicated that KSS treatment alleviated irritability, easy anger, anxiety, emotional instability, abdominal distension, coldness, and stiff shoulders.The WPAI activity impairment score was lower in the KSS group, and overall impression favoured in KSS treatment.. The only adverse event related to KSS was headache in one case. These exploratory findings suggest that KSS may improve menstrual-related symptoms in PMS.

## Introduction

Premenstrual syndrome (PMS) is characterized by a variety of symptoms, including mood disturbances, physical discomfort, and behavioral changes, which usually occur during the luteal phase of the menstrual cycle and subside shortly after menstruation begins [1]. PMS has the potential to impair substantially women’s social, work, and academic performance [2]. In Japan, PMS is defined as the presence of psychological or physical symptoms during the luteal phase (from 3 to 10 days before menstruation), which diminish or disappear with the beginning of menstruation. PMS is diagnosed according to the criteria established by the American College of Obstetricians and Gynecologists. Among PMS symptoms, psychological symptoms include signs of depression, anxiety, anger explosion, irritability, and mood swings, while physical symptoms include breast tenderness, abdominal bloating, headache, fatigue, lower abdominal pain, and edema [3,4].

Treatment for PMS includes non-pharmacological therapies such as counseling, lifestyle improvement, exercise therapy, and cognitive behavioral therapy [4]. In terms of pharmacotherapy, oral contraceptive pills (OCPs), low-dose estrogen/progestin formulations, and selective serotonin reuptake inhibitors are considered effective treatment options for PMS [5]. Moreover, Kampo medicines, including kamishoyosan (KSS), are recommended for a wide variety of symptoms in the Japanese guideline [5].

The Kampo medicine KSS is prescribed for the treatment of neuropsychiatric symptoms such as anxiety and irritability, as well as physical symptoms associated with changes in female hormone levels during menstruation and menopause. According to a survey on PMS/premenstrual dysphoric disorder (PMDD) treatment conducted on obstetricians and gynecologists in Japan, the most commonly used medication was OCPs (98.1%), followed by KSS (73.6%). As a first-line treatment, OCPs were the most commonly prescribed in 76.8% of cases, followed by Kampo medicine in 19.5% [6]. However, the therapeutic effect of KSS for PMS remains poorly understood. In a double-blind, placebo-controlled randomized clinical trial for patients with menopausal disorder, KSS showed the potential to improve irritability in premenopausal women, indicating a potential therapeutic effect on symptoms induced by fluctuating female hormones [7]. At the same time, some of the mechanisms underlying the anxiolytic/antidepressant effects of KSS have been investigated [8,9]. Furthermore, the involvement of serotonin (5-HT) and γ-aminobutyric acid (GABA) receptors has been proposed as the mechanism of action of KSS [10,11]. Regarding treatment for PMS, KSS is well recognized, but its effectiveness remains unclear. Therefore, we conducted an open-label, randomized controlled trial with the aim of evaluating the efficacy and safety of KSS for PMS.

## Methods

### Study drug

This study used KSS extract granules for ethical use (TSUMURA & CO., Tokyo, Japan; see STORK: http://mpdb.nibiohn.go.jp/stork/). KSS has been approved for medicinal use by the Japanese Ministry of Health and Welfare. KSS contains 4.0 g of a dried extract of the following mixed crude drugs: Japanese Pharmacopoeia (JP) Bupleurum root 3.0 g, JP Peony root 3.0 g, JP *Atractylodes lancea* rhizome 3.0 g, JP Japanese Angelica root 3.0 g, JP *Poria sclerotium* 3.0 g, JP Gardenia fruit 2.0 g, JP Moutan bark 2.0 g, JP Glycyrrhiza 1.5 g, JP Ginger 1.0 g, and JP Mentha herb 1.0 g. The KSS production process was regulated by the Drug Good Manufacturing Practice (GMP) issued by the Ministry of Health, Labour and Welfare of Japan and the self-imposed Ethical Kampo Medicine GMP regulations of the Japan Kampo Medicine Manufacturing Association. The KSS medication used in this study has been recognized as an approved indication from the Certified Review Board (CRB).

### Design

This open-label, randomized, exploratory trial was conducted to evaluate the efficacy and safety of KSS for PMS. The CRB of Tottori University Hospital approved this study on 01/03/2022 (Certification No.: 21C004). All study procedures were conducted in accordance with the Declaration of Helsinki and Clinical Trials ACT in Japan. This trial was registered with the Japan Registry of Clinical Trials and published on 11/04/2022. (jRCTs061220001; https://jrct.mhlw.go.jp/latest-detail/jRCTs061220001). The detailed protocol has been published elsewhere [12]. In brief, the study was conducted at a single center at Tottori University Hospital in Japan. Patients with PMS were enrolled from April 2022 to March 2024. The investigators and sub-investigator verified the eligibility of potential participants based on the inclusion and exclusion criteria and obtained their written informed consent before the initiation of any study-specific procedures. The study design and flow are shown in Fig 1, and the eligibility criteria are shown in Table 1. This study consisted of a KSS treatment group that received the study drug and an observation group that received no study drug. Patients were randomly assigned to either the KSS treatment or observation group. The primary endpoint was the Menstrual Distress Questionnaire (MDQ) score, which assesses the automatic imbalance syndrome peculiar to women in KSS indications. The allocation sequence was implemented using the APS-Cloud randomization framework. Participants were randomly assigned to the KSS treatment group or the observation group, stratified by MDQ scores during the pretreatment period (≥ 80 or not). A permutation block randomization method with a 1:1 allocation ratio was conducted for the random allocation. Only the data manager had access to this system.

**Table 1:** Criteria.

Inclusion criteria
• Patients diagnosed with PMS based on ACOG^a^ PMS diagnostic criteria
• Patients with a total score of ≥ 30 on the premenstrual MDQ^b^ at the pretreatment period
• Patients aged 18–45 years at the time of consent
• Patients with a regular menstrual cycle of 25–38 days
• Patients who can take the study drug orally
• Outpatients
• Written informed consent provided to participate in the study
Exclusion criteria
• The use of oral contraceptives, sex hormones, antidepressants, anxiolytics, sleeping pills, diuretics, or effective drugs against PMS, including supplements, within 4 weeks before obtaining consent
• Serious mental disease
• Serious comorbidities (e.g., gynecological disease, liver disease, renal disease, heart disease, blood disease, metabolic disease, malignant tumor)
• Patients who are pregnant, may be pregnant, or wish to become pregnant
• Lactating women
• History of allergies to Kampo medicine
• Patients who participated in clinical or other studies within 3 months before obtaining consent
• Patients treated with Kampo medicine, including the study drug, within 4 weeks prior to consent
• Any other medical condition that makes the patient unsuitable for inclusion in the study according to the opinion of the investigator
Discontinuation criteria
• Consent is withdrawn.
• Investigators judge that it is difficult to continue the study because of the deterioration of the patient’s primary disease, exacerbation of complications, or adverse events.
• Pregnant
• Investigators agree to discontinue the study.

^a^ACOG: American College of Obstetricians and Gynecologists, ^b^MDQ: Menstrual Distress Questionnaire.

**Fig 1:**
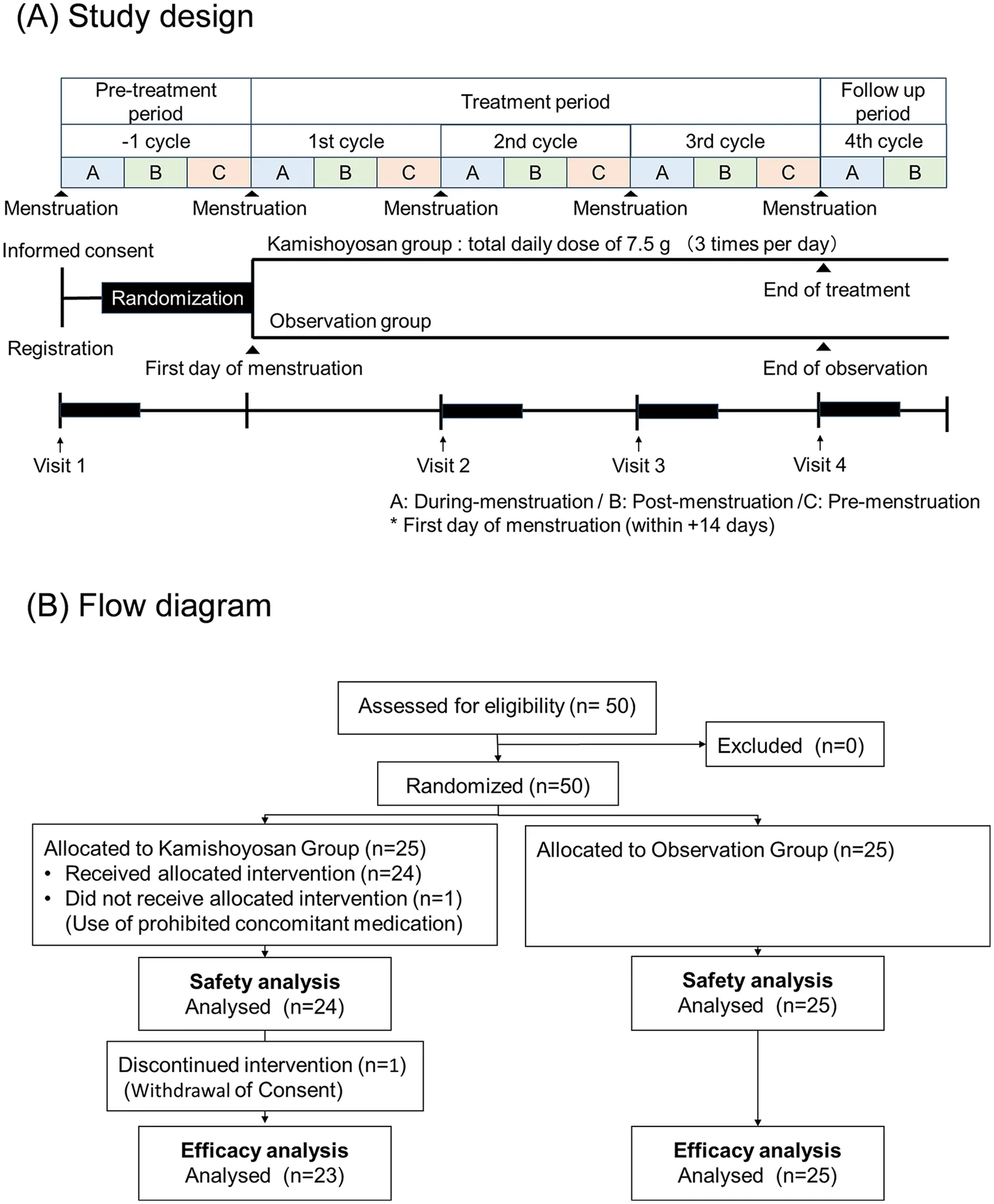
Study design and flow diagram of the study subjects. (A) This was an open-label, randomized control study. The total study duration was four menstrual cycles. The participants were assigned to a KSS treatment group or an observation group stratified by MDQ scores during the pretreatment period (≥ 80 or not). KSS treatment started the first day of menstruation after enrollment, which was administered orally for four menstrual cycles. (B) Flow diagram of the patients through the study phases. A total of 50 patients were enrolled. The safety analysis population included 49 patients (n = 24 in the KSS group and n = 25 in the observation group), and the efficacy analysis population included 48 participants (n = 24 in the KSS group and n = 25 in the observation group).

The CONSORT checklist is provided in S1 File, the study protocol is provided in S2 File, and the English translation of the study protocol is provided in S3 File.

### Interventions

The menstrual cycle is categorized into three phases: during menstruation, post-menstruation, and pre-menstruation based on the first day of menstruation. This study consisted of a pretreatment, a treatment, and a follow-up period (Fig 1A). Informed consent was obtained during the pretreatment period, along with the confirmation of eligibility, registration, and randomization. The treatment period was set as three cycles, with the study drug administered from the first day of menstruation in the first cycle. The study drug (KSS) was administered orally at 7.5 g/day in three divided doses (before or between meals) for the third cycle. The participants visited the hospital on the first day of menstruation (within + 14 days) for treatment and during the follow-up period for assessments and observations. Questionnaires were collected on the first day of menstruation during the second and third cycles, as well as the fourth cycle of the follow-up period. Moreover, throughout the study period, participants used the smartphone app YaDoc® (Integrity Healthcare Co., Ltd., Tokyo, Japan) to perform assessments of daily symptoms (once per day, at the same time whenever possible) in regard to sleep quality, Kampo medicine evaluation-related mental status, and menstruation-related symptoms.

### Outcomes

The primary outcome was the change in the premenstrual MDQ total score from baseline (–1 cycle) to the final evaluation. The secondary outcomes were as follows: 1) change in MDQ scores (premenstrual total, during menstrual total, post-menstrual total, and each subscale) over time; 2) sleep quality evaluation (daytime drowsiness, sleep satisfaction); 3) Kampo medicine evaluation-related mental status (irritability, easily angered, anxiety, emotional instability); 4) menstruation-related symptoms (headache, easy fatigue, excessive appetite, abdominal distension), for which assessment items 2–4 were rated on a numerical rating scale (NRS) from 0 to 10, recorded in YaDoc®, and classified by menstrual cycle (hereafter, daily symptom questionnaires); 5) labor productivity assessment based on the Work Productivity and Activity Impairment (WPAI) Questionnaire; 6) coldness and stiff shoulder score (with symptom severity assessed using an NRS from 0 to 10); 7) overall impressions after participating in the study (with scores assessed using an NRS from 1 to 7; e.g., at the completion of treatment, the patients were asked whether their PMS had changed since they started treatment; 8) sleep status and heart rate evaluation using a smartwatch (Fitbit Versa 3; Fitbit, San Francisco, CA, USA) every day at bedtime; and 9) the incidence of adverse events. Adverse events were observed from the start of the treatment period until the end of the follow-up period. The investigators confirmed the abnormality of subjective symptoms and abnormal changes in laboratory tests when the participants visit the hospital.

### Sample size and statistical analysis

The sample size was designed based on a two-sample t-test to detect a 6.0 mean difference with a 6.73 standard deviation (SD) for the primary outcome (MDQ total score), with a two-sided α level of 0.05 and 80% power between groups [12]. Assuming a 20% dropout rate, a minimum of 25 participants per group (a total of 50) was considered necessary.

For the primary efficacy analysis, a mixed-effects model for repeated-measures (MMRM) was used to estimate the mean difference in MDQ total scores [13]. The MMRM included repeated measurements of the MDQ total score as outcome variables, and the covariance matrix structure was set to unstructured. The covariance matrix structure was specified as unstructured because this approach places minimal assumptions on the correlation pattern among repeated measurements. The regression model included fixed effects for group, time (categorical), and group-by-time interactions, adjusted for the baseline MDQ score. The standard error estimates and degrees of freedom were adjusted using the Kenward–Roger method [14]. The least-squares (LS) means and mean differences in the change score were calculated based on the MMRM estimates. The primary analysis was conducted using MMRM, which uses all available observations under the missing-at-random assumption.

The secondary endpoints for efficacy and safety were analyzed in an exploratory manner. For the other MDQ scores (premenstrual total, mid-menstrual total, post-menstrual total, and each subscale score), we also performed MMRM analyses via the same steps used in the primary efficacy analysis. No adjustment for multiplicity was performed for secondary, subscale, or time-specific analyses; therefore, the corresponding P-values should be interpreted as nominal and exploratory and should be interpreted as hypothesis-generating rather than confirmatory. To assess Kampo medicine daily symptom questionnaires, coldness and stiff shoulder score, labor productivity, and overall impressions after treatment, we calculated summary statistics for individual cycles for the observation and KSS groups. The first four questionnaires were evaluated using Student’s *t* test, and overall impressions were evaluated using Fisher’s exact test to assess differences between groups. NRS outcomes were treated as approximately continuous variables for analysis, consistent with common practice in clinical research. All reported adverse events were summarized using basic statistics by event and degree of severity. All P-values were two-sided, with the significance level set at 5%. R version 4.4.3 (R Foundation for Statistical Computing, Vienna, Austria) was used for the statistical computations. The Full Analysis Set (FAS) consisted of all randomized participants who had available post-baseline efficacy data. Missing values were not imputed, and the analysis was conducted on theFAS set.

The change in the MDQ total score was expressed as the LS mean and 95% confidence interval (CI) range. The MDQ total score for the menstrual cycle was expressed as the mean ± SD. The MDQ subscales, Kampo medicine evaluation-related mental status, and menstruation-related symptoms were shown as means in a heatmap. Coldness, stiff shoulders, and WPAI were expressed as mean ± SD. The population displayed the overall impression.

### Data collection and management

This study used an electronic patient-reported outcome data capture system (Tottori University Hospital Research Support System) to collect participant data, along with a paper-based report form from YaDoc® and Fitbit. The collected data were stored in a secure database server. Monitoring was conducted to ensure reliability and safety. The study monitor reviewed the consent forms, medical records, and case report forms of all participants.

## Results

### Participant characteristics

A total of 50 women were enrolled in this study and randomly allocated to the KSS group (n = 25) and the observation group (n = 25). Two patients in the KSS group discontinued the study because of the use of restricted medications and the withdrawal of consent during the second cycle. The safety analysis population included 49 patients (n = 24 in the KSS group and n = 25 in the observation group). The efficacy analysis population included 48 participants (n = 24 in the KSS group and n = 25 in the observation group up to the second cycle, and n = 23 in the KSS group and n = 25 in the observation group from the third cycle onwards) (Fig 1B). No significant differences in patient characteristics were observed between the two groups (Table 2).

**Table 2:** Patient characteristics.

Patient characteristics	Kamishoyosan group (n = 24)	Observation group (n = 25)
Age at consent (years)	29.2 ± 9.2	29.1 ± 8.9
Age at menarche (years)	11.7 ± 1.2	12.4 ± 1.4
Allergy history (Yes/No)	10 (41.7%)/ 14 (58.3%)	7 (28.0%)/ 18 (72.0%)
Height (cm)	158.2 ± 6.0	159.0 ± 5.0
Weight (kg)	54.9 ± 8.4	52.3 ± 7.6
BMI (kg/m^2^)	22.0 ± 3.5	20.7 ± 3.0
Systolic BP (mmHg)	113.7 ± 16.2	113.5 ± 11.4
Diastolic BP (mmHg)	66.2 ± 12.9	67.6 ± 11.1
Pulse rate	75.9 ± 12.3	80.0 ± 11.6
γ-GTP (IU/L)	16.4 ± 5.9	17.8 ± 9.4
AST (IU/L)	18.0 ± 4.5	20.0 ± 6.0
ALT (IU/L)	15.4 ± 6.8	21.1 ± 12.9
Na (mEq/L)	140.6 ± 1.6	141.0 ± 1.5
K (mEq/L)	4.1 ± 0.3	4.1 ± 0.2
Menstrual cycle (days)	29.3 ± 2.4	30.2 ± 2.8
Stiff shoulders (Yes/No)	19 (79.2%)/ 5 (20.8%)	17 (68.0%)/ 8 (32.0%)
Headache (Yes/No)	13 (54.2%)/ 11 (45.8%)	10 (40.0%)/ 15 (60.0%)
Dizziness (Yes/No)	3 (12.5%)/ 21 (87.5%)	5 (20.0%)/ 20 (80.0%)
Burning sensation (Yes/No)	4 (16.7%)/ 20 (83.3%)	5 (20.0%)/ 20 (80.0%)
Paroxysmal episodes (Yes/No)	1 (4.2%)/ 23 (95.8%)	2 (8.0%)/ 23 (92.0%)
Coldness (Yes/No)	11 (45.8%)/ 13 (54.2%)	13 (52.0%)/ 12 (48.0%)
Constipation (Yes/No)	12 (50.0%)/ 12 (50.0%)	13 (52.0%)/ 12 (48.0%)
Skin issues (Yes/No)	14 (58.3%)/ 10 (41.7%)	10 (40.0%)/ 15 (60.0%)
Easy fatigue (Yes/No)	13 (54.2%)/ 11 (45.8%)	18 (72.0%)/ 7 (28.0%)
Balanced diet (Yes/No)	16 (66.7%)/ 8 (33.3%)	18 (72.0%)/ 7 (28.0%)
Exercise habit (Yes/No)	4 (16.7%)/ 20 (83.3%)	5 (20.0%)/ 20 (80.0%)
Alcohol consumption (Yes/No)	1 (4.2%)/ 23 (95.8%)	2 (8.0%)/ 23 (92.0%)
Sleep difficulty (Yes/No)	9 (37.5%)/ 15 (62.5%)	13 (52.0%)/ 12 (48.0%)
Medical history (Yes/No)	4 (16.7%)/ 20 (83.3%)	5 (20.0%)/ 20 (80.0%)
Comorbidities (Yes/No)	9 (37.5%)/ 15 (62.5%)	3 (12.0%)/ 22 (88.0%)
Edema (Yes/No)	5 (20.8%)/ 19 (79.2%)	7 (28.0%)/ 18 (72.0%)
Abnormal physical findings (Yes/No)	0 (0.0%)/ 24 (100.0%)	1 (4.0%)/ 24 (96.0%)

Data are expressed as the mean ± SD or number (%).

### Evaluation of the treatment based on questionnaires

#### MDQ total score.

The primary outcome was the change in premenstrual MDQ scores from baseline to the third cycle (Visit 4). The change was –4.0 (95%CI: –11.9 to 3.9) in the observation group and –21.6 (95%CI: –29.7 to –13.4) in the KSS group. The LS mean difference in change between the two groups was –17.6 (95%CI: –28.9 to –6.2, p = 0.0031), which reached the primary endpoint (Fig 2A). Furthermore, the change in premenstrual MDQ scores at the first cycle were 4.7 (95%CI: –3.7 to 13.2) in the observation group and –8.0 (95%CI, –16.7 to 0.7) in the KSS group. The LS mean indicates that the difference between the two groups at the first cycle was –12.7 (95%CI: –24.8 to –0.6; p = 0.040). At the second cycle, the change in premenstrual MDQ scores was –0.46 (95%CI: −8.4 to 7.5) in the observation group and –14.9 (95%CI: –23.0 to –6.7) in the KSS group. The LS mean difference between the two groups at the second cycle was –14.4 (95%CI: –25.8 to –3.1, p = 0.014).

**Fig 2:**
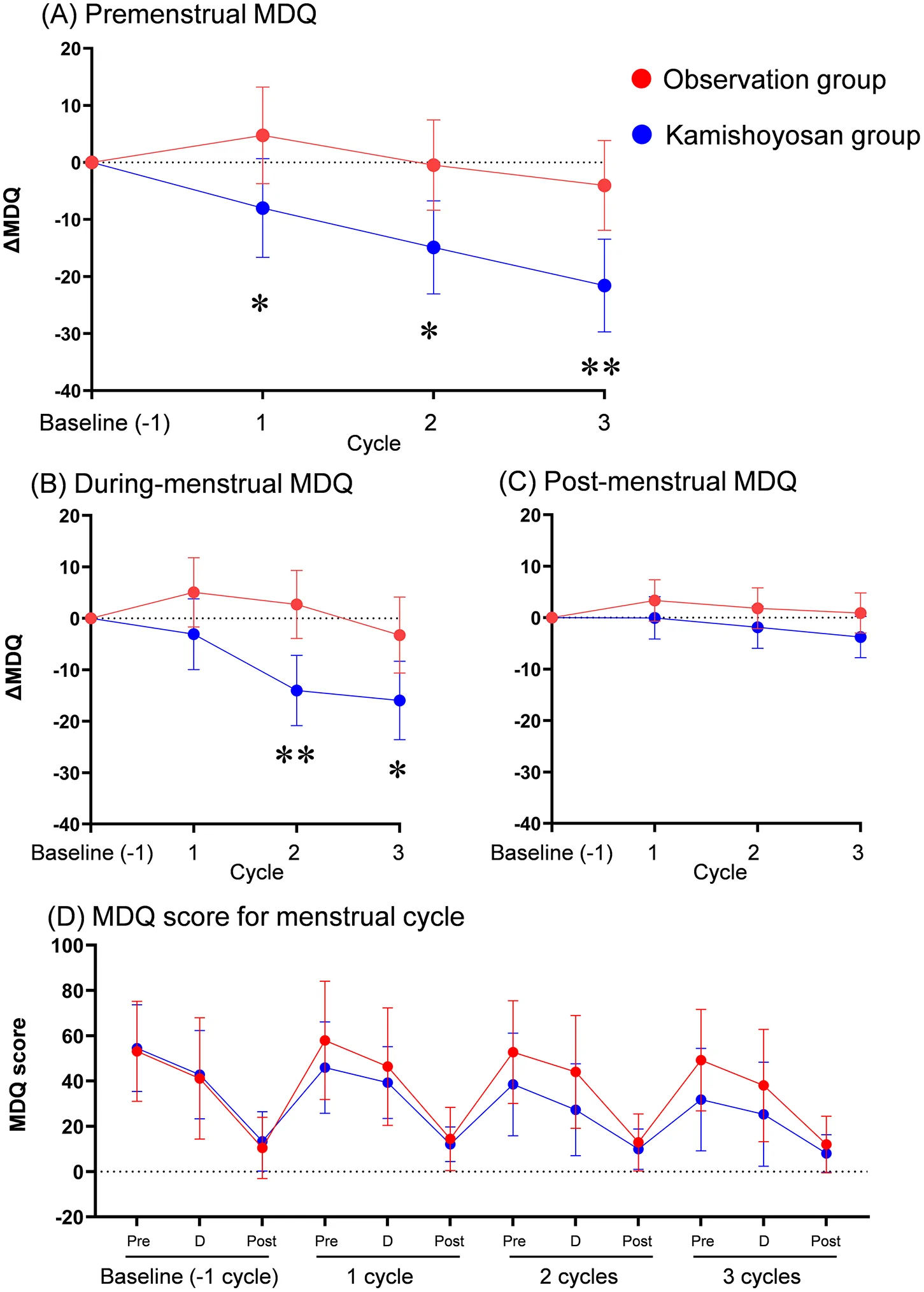
Evaluation of KSS treatment based on the MDQ total score. (A) Change in the premenstrual MDQ total score. The primary endpoint, the LS mean difference in premenstrual MDQ at the third cycle, was –17.6 (95%CI: –28.9 to –6.2, p = 0.0031, Observation group; n = 25, KSS group; n = 23). The LS mean difference in change between the two groups was –12.7 (95%CI: –24.8 to –0.6; p = 0.040) at the first cycle and –14.4 [95%CI: –25.8 to –3.1, p = 0.014] at the second cycle. (B) Change in the during menstrual MDQ total score. The LS mean difference in change between the two groups was –8.1 (95%CI: –17.8 to 1.5, p = 0.096) at the first cycle, –16.7 (95%CI: –26.2 to –7.3, p = 0.0009) at the second cycle, and –12.7 (95%CI: –23.3 to –2.1, p = 0.020) at the third cycle. The LS mean differences showed a decrease in the second and third cycles in the KSS group. (C) Change in the post-menstrual MDQ total score. No significant differences were observed at any time point between the two groups. (D) Changes in MDQ scores. (A)–(C) Data are expressed as the LS means and [95%CI range]. (D) Data are expressed as the mean ± SD. (Observation group; n = 25, KSS group; n = 23–25). The comparison at the third cycle in Fig 2(A) represents the primary endpoint. All remaining p-values should be interpreted as nominal. *p < 0.05, **p < 0.01 (MMRM analyses).

The secondary outcomes were changes in the during- and post-menstrual MDQ total scores and each subscale score over time. The LS mean difference between the two groups in the menstrual MDQ total score was –8.1 (95%CI: –17.8 to 1.5, p = 0.096) at the first cycle, –16.7 (95%CI: –26.2 to –7.3, p = 0.0009) at the second cycle, and –12.7 (95%CI: –23.3 to –2.1, p = 0.020) at the third cycle (Fig 2B). No significant differences in the post-menstrual MDQ total score were observed between the two groups at any time point (Fig 2C).

#### MDQ subscales.

A heatmap of MDQ subscale scores is shown in Fig 3. The following subscales on the premenstrual MDQ showed nominally significant between-group differences in LS mean changes:: Pain: –2.4 (95%CI: –4.4 to –0.4, p = 0.020) at the third cycle; Concentration: –4.7 (95%CI: –7.6 to –1.7, p = 0.0025) at the second cycle and –3.9 (95%CI: –6.9 to –0.9, p = 0.012) at the third cycle; Behavioral change: –2.3 (95%CI: –4.1 to –0.4, p = 0.019) at the third cycle; Fluid accumulation: –1.4 (95%CI: –2.7 to –0.2, p = 0.029) at the first cycle; and Negative feeling: –4.5 (95%CI: –7.7 to –1.3, p = 0.0069), –4.7 (95%CI: –7.4 to –2.0, p = 0.0010), and –5.0 (95%CI: –8.0 to –2.0, p = 0.0015) at the first, second, and third cycles, respectively.

**Fig 3:**
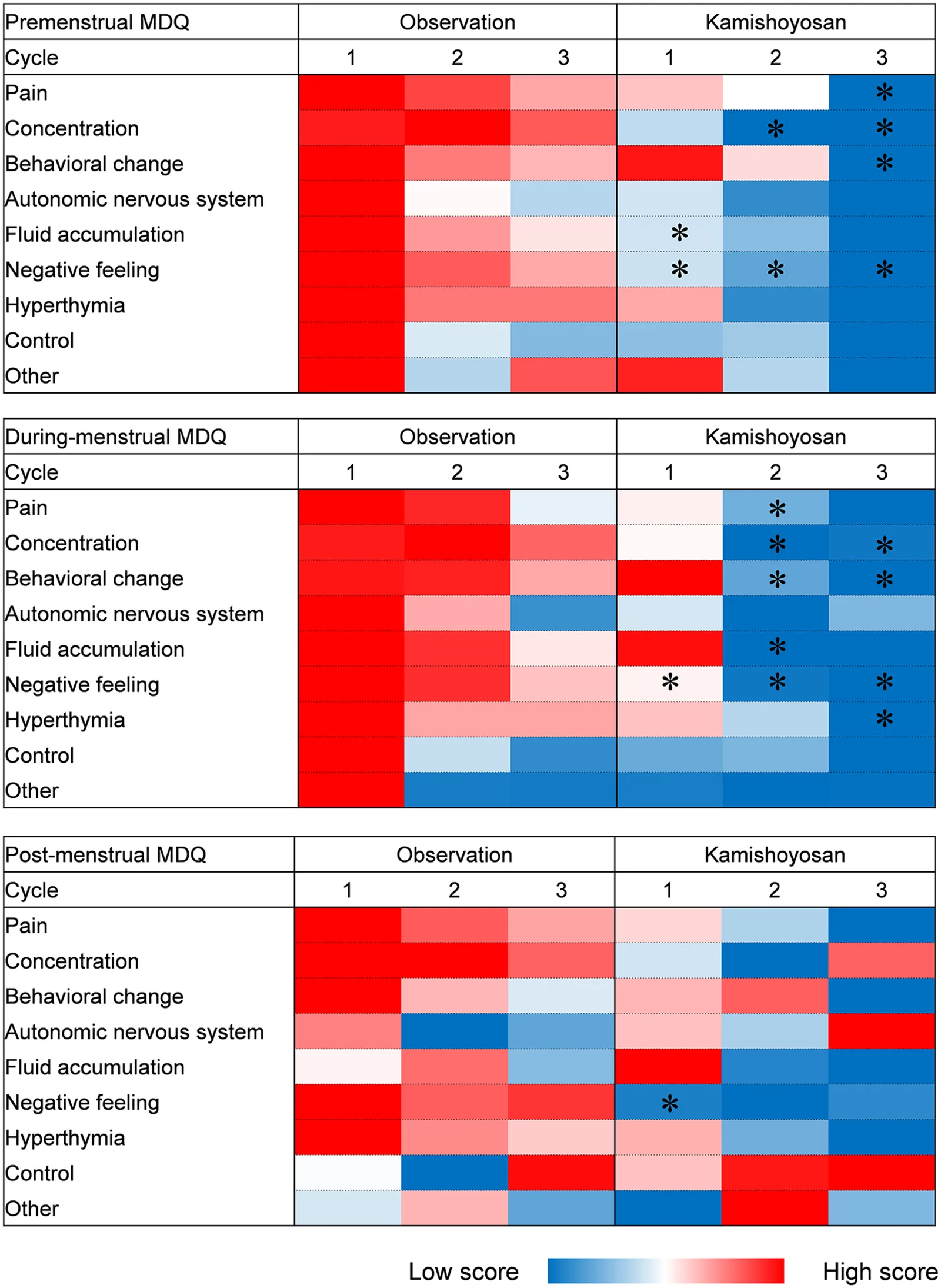
Evaluation of KSS treatment based on the MDQ subscales. Heatmap showing the LS means of each subscale (Observation group; n = 25, KSS group; n = 23–25). The red and blue regions indicate high and low scores relative to the LS means of each row, respectively. MDQ subscale scores generally decreased in the KSS group compared with the observation group. Nominal between-group differences (nominal p < 0.05) were observed for the following subscales: premenstrual; pain at the third cycle, concentration at the second and third cycles, behavioral change at the third cycle, fluid accumulation at the first cycle, negative feeling at the first to third cycles: during menstrual; pain at the second cycle, concentration at the second and third cycles, behavioral change at the second and third cycles, fluid accumulation at the second cycle, negative feeling at the first to third cycles, hyperthymia at the third cycle: post-menstrual; negative feeling at the first cycle. * nominal p < 0.05, ** nominal p < 0.01 (MMRM analyses).

The following subscales of the during menstrual MDQ showed between-group differences in LS mean changes:: Pain: –1.9 (95%CI: –3.7 to –0.1, p = 0.036) at the second cycle; Concentration: –4.9 (95%CI: –7.4 to –2.4, p = 0.0002) and –3.8 (95%CI: –6.4 to –1.2, p = 0.0047) at the second and third cycles, respectively; Behavioral change: –2.5 (95%CI: –4.2 to –0.8, p = 0.053) and –2.2 (95%CI: –4.2 to –0.2, p = 0.031) at the second and third cycles, respectively; Fluid accumulation: –1.2 (95%CI: –2.3 to –0.2, p = 0.025) at the second cycle; Negative feeling: –2.5 (95%CI: –5.0 to –0.1, p = 0.040), –4.7 (95%CI: –7.2 to –2.3, p = 0.0003), and –3.3 (95%CI: –5.9 to –0.8, p = 0.011) at the first, second, and third cycles, respectively; and Hyperthymia: –1.3 (95%CI: –2.3 to –0.3, p = 0.013) at the third cycle. Among the post-menstrual subscale MDQ scores, a nominally significant between-group difference in LS mean change was observed only for “Negative feeling” during the first cycle (–1.5, 95%CI: –2.9 to –0.1, p = 0.038).

#### Daily symptom questionnaires.

A heatmap of diary symptoms (NRS scores) is shown in Fig 4. In the patient diaries during pre-menstruation, between-group differences were observed for irritability, easy anger, anxiety, and emotional instability in the second cycle were observed between the observation and KSS groups. In the third cycle, lower scores were observed in the KSS group for anxiety, emotional instability, easy fatigue, and abdominal distention. During menstruation, between-group differences were observed for excessive appetite in the second cycle and for anxiety and emotional instability in the third cycle. In post-menstruation, lower emotional instability scores were observed in the KSS group. However, baseline imbalances in anxiety and emotional instability were observed before treatment. Accordingly, the corresponding exploratory between-group findings should be interpreted cautiously and should not be regarded as evidence of treatment effects independent of these baseline differences.

**Fig 4:**
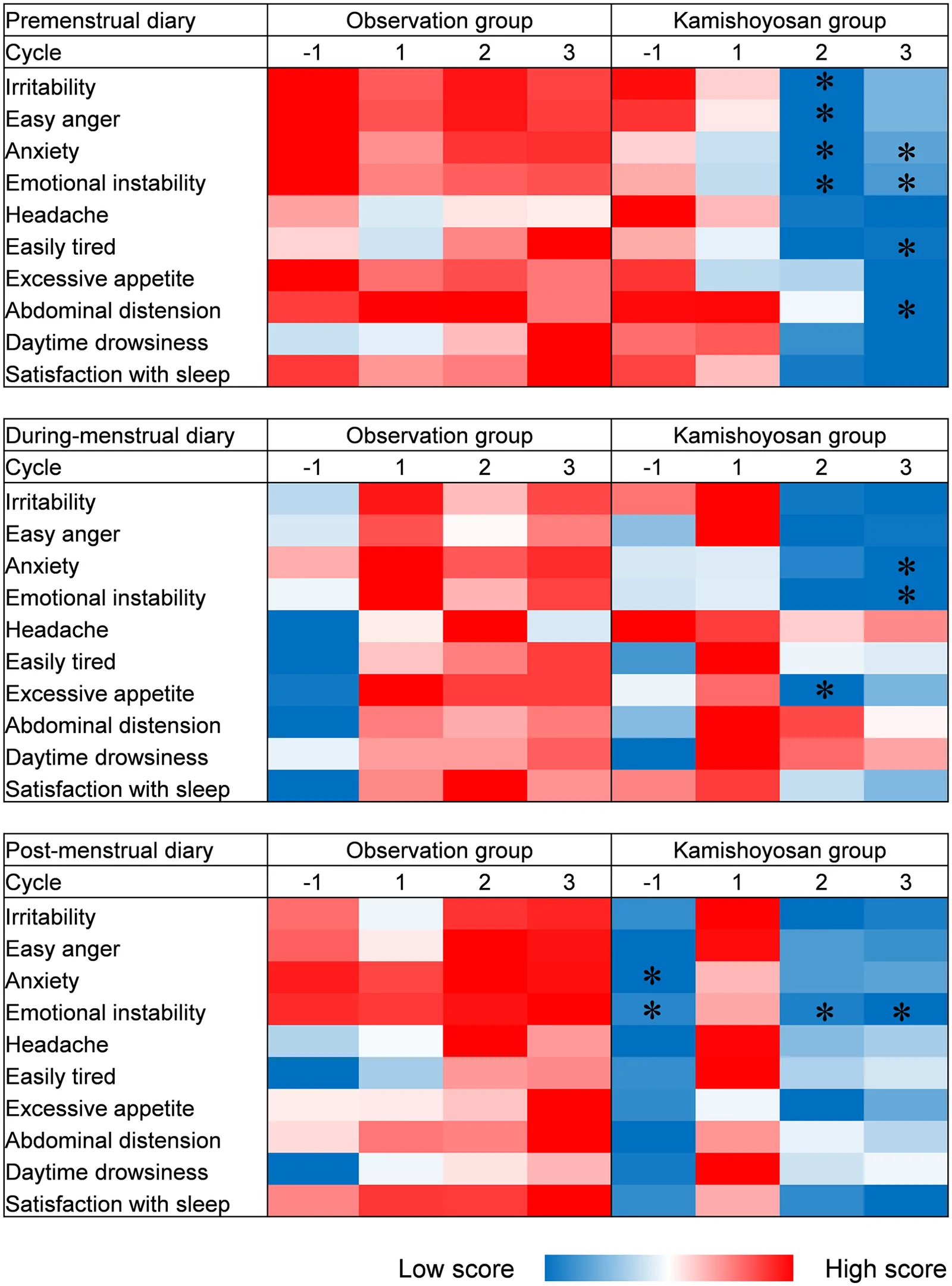
Evaluation of KSS treatment based on symptom diaries. Heatmap showing the means of each daily symptom questionnaire (Observation group; n = 25, KSS group; n = 23–25). The red and blue regions indicate high and low scores relative to the means of each row, respectively.

Between-group differences were observed for the following diary symptoms:: premenstrual; irritability at the second cycle, easy anger at the second cycle, anxiety at the second and third cycles, emotional instability at the second and third cycles, easy fatigue at the third cycle, abdominal distension at the third cycle: during menstrual; anxiety at the third cycle, emotional instability at the third cycle, excessive appetite at the second cycle: Baseline differences were observed for post-menstrual anxiety and emotional instability. *nominal p < 0.05, **nominal p < 0.01 (Student’s *t* tes*t*).

#### Coldness and stiff shoulder scores.

The coldness score is presented in Fig 5A. The coldness scores for the observation group were 3.4 ± 2.8, 3.7 ± 2.4, 3.0 ± 2.4, and 2.4 ± 2.3 (at the first to fourth visits, respectively). In comparison, the scores for the KSS group were 2.7 ± 2.2, 1.9 ± 2.4, 1.2 ± 1.3, and 1.2 ± 1.5 (at the first to fourth visits, respectively). The lower coldness scores were observed at the second, third, and fourth visits (p = 0.012, 0.004, and 0.033, respectively). In the observation group, the stiff shoulder scores were 4.1 ± 3.0, 4.6 ± 2.5, 4.3 ± 2.6, and 3.8 ± 2.7 (at the first to fourth visits, respectively, Fig 5B). The stiff shoulder scores for the KSS group were 4.7 ± 2.3, 3.7 ± 2.6, 3.5 ± 2.4, and 2.3 ± 1.7 (at the first to fourth visits, respectively). The lower stiff shoulder scores were observed at the fourth visit (p = 0.033).

**Fig 5:**
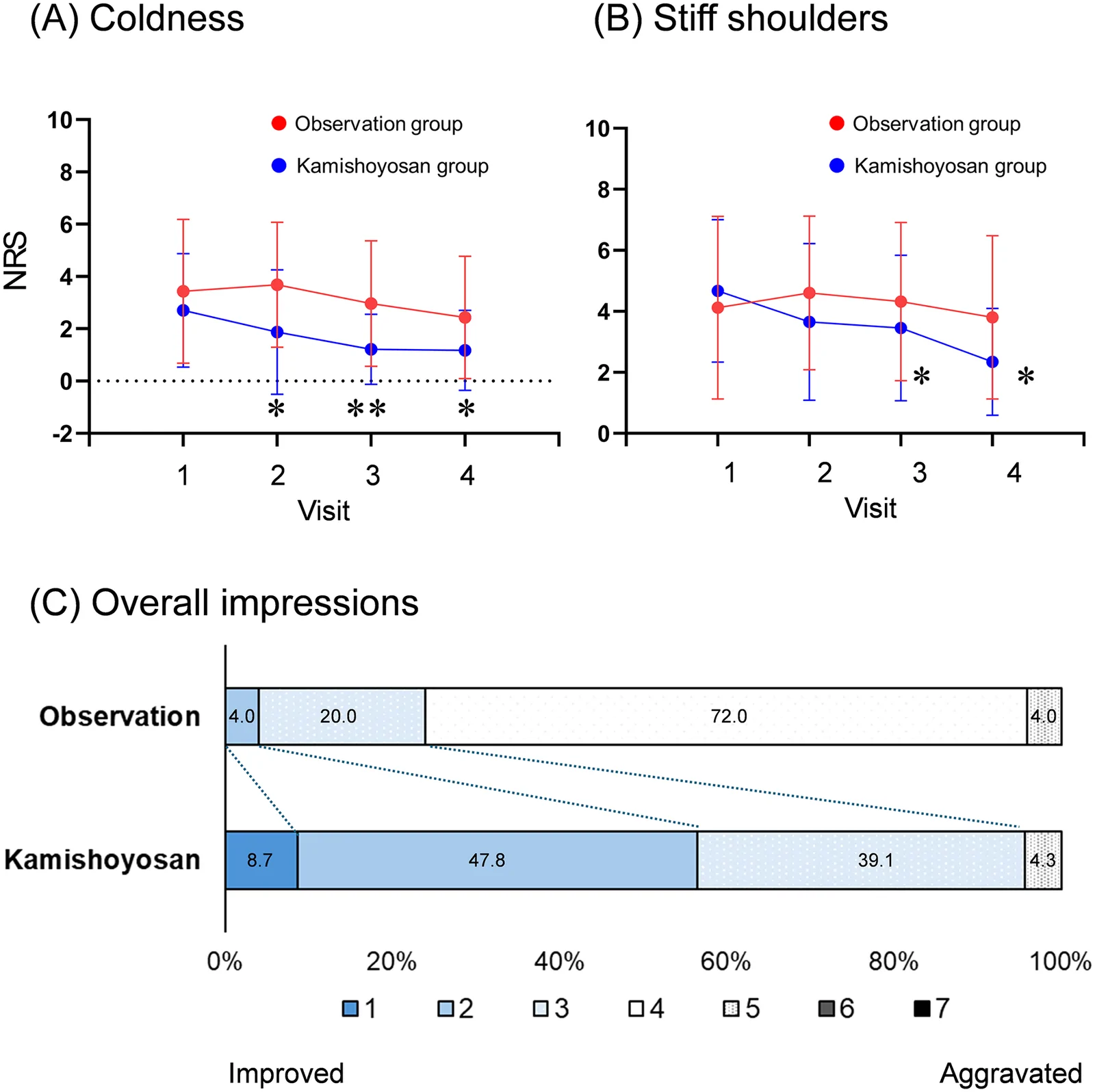
Evaluation of KSS treatment based on the coldness and stiff shoulder score and overall impressions. (A) Change in the premenstrual coldness score. Symptom severity was assessed using a 0 to 10 NRS. At the second visit, the observation group showed an NRS score of 3.7 ± 2.4 and the KSS 1.9 ± 2.4 (nominal p = 0.012). At the third visit, the observation group showed an NRS score of 3.0 ± 2.4 and the KSS group 1.2 ± 1.3 (p = 0.004). At the fourth visit, the observation group showed an NRS score of 2.4 ± 2.3 and the KSS group 1.2 ± 1.5 (p = 0.033). (B) Change in the premenstrual stiff shoulders score. At the fourth visit, the observation group showed an NRS score of 3.8 ± 2.7 and the KSS group 2.3 ± 1.7 (p = 0.033). Data are expressed as the mean ± SD (Observation group; n = 25, KSS group; n = 23–25). *p < 0.05, **p < 0.01 (Student’s *t* tes*t*). (C) Overall impressions after participating in the study. The ratings were made on an NRS (from 1, extremely improved, to 7, extremely aggravated). The lower the score, the better the symptoms, including PMS. In the observation group, 4.0% (n = 1) responded with 2, 20.0% (n = 5) with 3, 72.0% (n = 18) with 4, and 4.0% (n = 1) with 5. In the KSS group, 8.7% (n = 2) responded with 1, 47.8% (n = 11) with 2, 39.1% (n = 9) with 3, and 4.3% (n = 1) with 5. A between-group difference was observed in the distribution of ratings (p < 0.001, Fisher’s exact test). The p-values shown in this figure are nominal and should be interpreted accordingly.

#### Overall impressions.

Overall impressions are shown in Fig 5C. In the observation group, 4.0% (n = 1) responded with 2, 20.0% (n = 5) with 3, 72.0% (n = 18) with 4, and 4.0% (n = 1) with 5. In the KSS group, 8.7% (n = 2) responded with 1, 47.8% (n = 11) with 2, 39.1% (n = 9) with 3, and 4.3% (n = 1) with 5. A between-group difference was observed in (p < 0.001).

#### WPAI scores.

The WPAI scores are shown in Fig 6. (A) absenteeism, (B) presenteesim and (C) work productivity loss represented the analysis for workers only. A total of 26 workers were enrolled in this study, consisting of an observation group (n = 12) and a KSS group (n = 14). (D) Activity impairment was evaluated in participants including non-workers (Observation group, n = 25; KSS group, n = 23). Lower activity impairment scores were observed in the KSS group at visits 3 and 4. (nominal p = 0.012 and 0.041, respectively).

**Fig 6:**
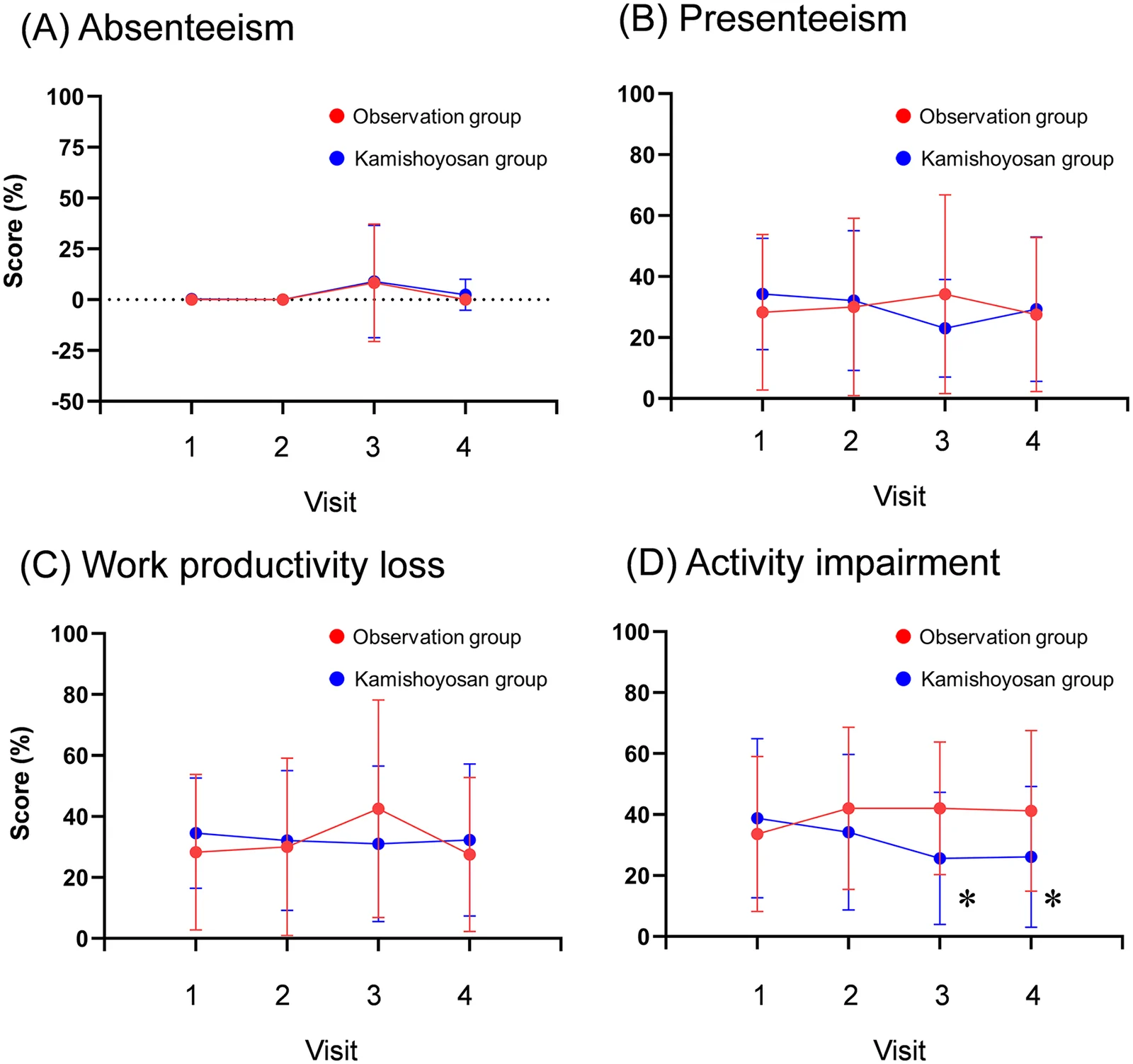
Evaluation of KSS treatment based on the WPAI. (A) Change in the premenstrual absenteeism score. (B) Change in the premenstrual presenteeism score. (C) Change in the premenstrual work productivity loss score. No between-group differences were observed for (A) – (C) at any visit (Observation group, n = 12; KSS group, n = 14). (D) Change in the premenstrual activity impairment score. Lower activity impairment scores were observed in the KSS group at the third and fourth visits (Observation group: 42.0 ± 21.8 and 41.2 ± 26.4; KSS group: 25.7 ± 21.7 and 26.1 ± 23.1; p = 0.012 and 0.041, respectively). Data are expressed as the mean ± SD (Observation group; n = 25, KSS group; n = 23). *nominal p < 0.05 (Student’s *t t*est). Absenteeism = percent worktime missed during the past 7 days. Presenteeism = percent impairment while working in the last 7 days. Work productivity loss = percent overall (absenteeism + presenteeism) in the last 7 days. Activity impairment = percent impaired while doing regular daily activities, other than work at a job.

#### Sleep status and heart rate evaluation (Fitbit).

The integrated results for the sleep and heart rate data collected by Fitbit are shown in the supporting information. However, many missing values were found because of incorrect device attachment. According to the Fitbit measurements, significant baseline differences in the following items were observed between the observation and KSS groups: total sleep time, time spent awake, wake after sleep onset (WASO), and light levels. Significant differences in light levels were also observed between the two groups at menstruation and post-menstruation within the first cycle. In addition, WASO was significantly lower in the KSS group. Given the substantial missing data and the baseline imbalances in several Fitbit measures, these results should be considered descriptive and hypothesis-generating and should not be interpreted as confirmatory evidence of a treatment effect (S1 Table).

### Adverse events and safety

Adverse events were observed in both groups. In the KSS group, there were 12 cases (30 events) of mild adverse events and three cases (five events) of moderate adverse events. In the observation group, there were nine cases (18 events) of mild adverse events and two cases (two events) of moderate adverse events. No severe adverse events were reported in either group. The only adverse event related to KSS was a headache in one case (two events). No participants discontinued the trial because of adverse events. Adverse events classified by the MedDRA organ system during the trial period.

## Discussion

The present randomized controlled clinical trial was conducted in an exploratory manner to evaluate the efficacy and safety of KSS for the treatment of PMS. The findings indicated an amelioration of the menstrual symptoms associated with PMS. In terms of safety, no serious adverse events were observed, and the incidence of side effects was low. These results suggest that KSS may have beneficial effects for PMS treatment.

The primary endpoint, the premenstrual MDQ score at the third cycle, decreased after treatment with KSS, suggesting a potential reduction in menstrual-related symptoms in patients with PMS (Fig 2A). Moreover, this improvement in the MDQ score was observed at an early stage, and the effect persisted throughout the third cycle. The observation group also showed a tendency toward improvement in premenstrual MDQ scores. PMS has been reported to be treated with counseling and cognitive behavioral therapy [15]. We believe that the self-monitoring effect of physician counseling and daily symptom diaries facilitated the improvement seen in the observation group.

We also investigated MDQ total scores during and post-menstruation. The KSS treatment group showed lower scoresin the MDQ total from the second cycle onward during menstruation (Fig 2B). Few reports have been published on the efficacy of KSS for menstrual symptoms, and its efficacy has not been sufficiently established in clinical practice. Accordingly, we analyzed MDQ trends over three cycles and observed a gradual improvement in symptoms (Fig 2D). These findings suggest that the improvement in the KSS group during pre-menstruation may be related to the subsequent improvement in menstrual symptoms, which are mitigated by premenstrual symptoms.

Fig 3 shows heatmap of the MDQ subscales. Among the subscales, the domains that showed lower scores during pre-menstruation in the KSS group were pain, concentration, behavioral change, and negative feelings (Fig 3). In addition to these domains, hyperthymia was also ameliorated during the menstrual cycle (Fig 3). In particular, the patients in the KSS group reported the amelioration of negative feelings and concentration in the early menstrual cycle. KSS is used empirically to treat the mental symptoms associated with hormonal changes in PMS and menopause [16,17]. The negative emotional domains improved by KSS included anxiety, irritability, mood swings, depression, and loneliness. These findings suggested that KSS may be associated with a reduction in several psychological symptoms of PMS; in addition, we clarified that its effects were exerted from an early cycle. In this study, daily symptom questionnaires were also recorded in a symptom diary using a smartphone app. The questionnaires indicated that irritability, anger, anxiety, and emotional instability improved from an early stage (Fig 4). The MDQ subscale and symptom diary NRS scores showed similar results for mental symptoms, which is consistent with the observed effects of KSS.. KSS has been shown to increase extracellular 5-HT levels in the prefrontal cortex and reduce anxiety-like behavior in a PMS-like rat model [10]. Furthermore, KSS was found to ameliorate anxiety-like behavior via GABA_A_ and dopamine D_1_ receptor-mediated mechanisms in an ovariectomy model [11]. Numerous clinical and preclinical studies have reported a correlation between GABAergic system and the pathophysiology of mood disorders [18]. GABA conductance and changes in levels of neurosteroids, especially allopregnanolone, are suspected to play a substantial role in the etiology of PMS/PMDD [19]. The results of the present study are consistent with these previous findings.

In this study, the KSS group also showed improvement in the pain domain (Fig 3). Kampo medicines have been used as remedies for various types of pain in Japan [20]. Concerning the symptoms associated with PMS, the presence of pain, including abdominal pain, pelvic pain, and headaches, has been observed to have adverse effects on quality of life (QOL) [21]; however, the underlying mechanisms remain unclear. Among the herbal ingredients in KSS, paeoniflorin (in peony root) has been demonstrated to exert anti-nociceptive effects via spinal α_2_ adrenergic receptors [22]. The analgesic effect of KSS is suggested to be related to the components of peony root. Moreover, anxiety has been associated with an exacerbation of pain, and there is a reciprocal link [23]. The antianxiety effect of KSS showed improvement from the early cycle and persisted through menstruation. Therefore, we considered the possibility that pain improvement is potentially correlated with anxiety relief. However, no improvement in headaches was seen based on the symptom diaries (Fig 4). Therefore, further investigation of pain is necessary.

PMS often involves sleep disorders, and there are reports that allopregnanolone and serotonin are involved [24,25]. The present study indicated a tendency toward increased sleep satisfaction in the KSS group (Fig 4). Sleep evaluation using wearable devices was also performed. Still, the number of cases analyzed was small as a result of missing values. KSS has been reported to improve sleep disorders in menopausal disorders [26]. Although sufficient sleep evaluation could not be performed in this study, these results support KSS-induced improvement in sleep, and thus, further studies are needed.

KSS has been empirically prescribed for coldness and stiff shoulders in a variety of women’s health issues. Coldness is regarded as a vasomotor symptom, and stiff shoulders are frequently caused by autonomic imbalance and blood flow disorders [27]. This study observed lower coldness and stiff shoulder scores in KSS treatment, which was empirically confirmed by the relief of these symptoms (Fig 5A, B). In the present study, participants in the KSS group reported more positive overall impressions.(Fig 5C). The amelioration of overall impression may be related to the alleviation of menstrual distress symptoms from the premenstrual phase through during-menstruation, in addition to improvements in daily activity impairment levels. As an important note, these results were obtained as an exploratory open-label clinical study and should be interpreted with caution.

Women’s health issues related to menstruation have become a social problem and lead to economic loss. The impact of menstruation-related symptoms on QOL and work productivity is a significant concern. Among WPAI assessments, lower activity impairment scores were observed in the KSS group. (Fig 6D). However, no significant effects were observed on absenteeism, presenteeism, or work productivity loss. These three scales are evaluations that target workers, and as such, the evaluation was limited because the number of workers included in this study was small.

In the present study, the only adverse effect observed with KSS administration was one case of mild headache; no symptoms exceeding grade 3 were observed. In addition, only one case was discontinued. These findings suggest that KSS was generally well tolerated throughout the study period.

Overall, the findings from the secondary analyses should be interpreted with caution, as these analyses involved numerous outcomes and assessment times without adjustment for multiplicity. Individual nominally significant findings should therefore be regarded as hypothesis-generating rather than as confirmatory evidence of treatment efficacy.

## Limitations

The study has several additional limitations. First, the trial design was open-label and not blinded; thus, the potential for a placebo effect cannot be excluded. Futhermore, as several outcomes were assessed using patient-reported outcome measures, including symptom questionnaires and global impression ratings, the therapeutic effects observed in this open-label study may have been overestimated due to expectation and reporting bias. Second, the sample size was relatively small, which may have limited the precision and stability of some estimates, particularly for secondary and exploratory analyses. Third, the study was exploratory, and no adjustment for multiplicity was performed for secondary, subscale, or time-specific analyses. Accordingly, these nominal findings should be regarded as hypothesis-generating rather than confirmatory. Chance baseline imbalances were also present for some exploratory outcomes, and these may have influenced the corresponding unadjusted comparisons. In addition, The Fitbit measurements were obtained as exploratory assessments, and interpretation requires caution due to missing data..

## Conclusion

The findings of this exploratory randomized trial suggest that KSS may improve menstrual-related symptoms in PMS. Additionally, no serious adverse events were reported during study. Taken together, these findings suggest the potential clinical utility of KSS for PMS.

## Supporting information

S1 Table Sleep status and heart rate evaluation using a smartwatch (Fitbit). TST = total sleep time; time spent awake = time spent awake in bed; WASO = wake after sleep onset (time spent awake during sleep); TIB = total time in bed; RMSSD = root mean square of successive differences; NREMHR = heart rate during non-REM sleep; LF/HF = ratio of low frequency to high frequency. *p < 0.05, **p < 0.01 (Student’s *t* test).
(XLSX)

S1 File CONSORT Checklist. (DOCX)

S2 File Study protocol. (PDF)

S3 File Protocol English Translation v1.3 for PLOS One (1). (PDF)
