# Supplementary material for: A Kampo medicine, kamishoyosan, ameliorates menstruation-associated symptoms in patients with premenstrual syndrome: An exploratory open-label, randomized controlled trial
Source: PLoS One. 2026 Nov 6;21(11):e0362303. doi: 10.1371/journal.pone.0362303 (PMC13742958; doi:10.1371/journal.pone.0362303)
Supplement: Supplementary file 1 [file pone.0362303.s003.pdf]

月経前症候群に対する加味逍遥散の有効性及び安全性を評価する

非盲検、無作為化臨床研究

臨床研究実施計画書
整理番号：21C004

研究責任医師：谷口　文紀　鳥取大学医学部附属病院　女性診療科　教授
住所：鳥取県米子市西町36-1
TEL：0859-38-6647／FAX：0859-38-6649

研究事務局：遠藤　佑輔　鳥取大学医学部附属病院　新規医療研究推進センター
住所：鳥取県米子市西町36-1
TEL：0859-38-6946／FAX：0859-38-6947

緊急連絡先：0859-38-6913（鳥取大学医学部附属病院　女性診療科病棟）
臨床研究実施予定期間：2022年4月（認定臨床研究審査委員会承認後）～2025年3月

作成日：2024 年 4 月 15 日（第 1.3 版）

0. 研究概要

0.1. 研究実施計画の概要

目的	月経前症候群（PMS）患者を対象に、加味逍遥散投与群と経過観察群を比較し、加味逍遥散の有効性及び安全性を評価する。
評価項目	【主要評価項目】<br>月経前の Menstrual Distress Questionnaire（MDQ）総スコアのベースライン時から最終評価時までの変化量<br>【副次評価項目】<br>1） 月経前、月経中、月経後の MDQ 総スコア、各下位尺度スコアの経時変化<br>2） 睡眠評価<br>3） 漢方医学的評価<br>4） 月経関連症状評価<br>5） 労働生産性評価<br>6） 冷え、肩こりスコア<br>7） 研究参加後の全体印象<br>8） Fitbit（スマートウォッチ）による睡眠評価・心拍評価<br>9） 有害事象
研究デザイン	非盲検、無作為化、臨床研究
選択基準	1） ACOG の PMS 診断基準に基づき PMS と診断された患者<br>2） プレ治療期の月経前の MDQ 総スコアが 30 点以上の患者<br>3） 同意取得時に 18 歳以上、45 歳以下の患者<br>4） 月経周期が 25 日から 38 日間の規則的な患者<br>5） 研究薬を経口服用できる患者<br>6） 受診区分：外来<br>7） 本研究への文書による参加同意が得られた患者
除外基準	1） 同意取得前 4 週間以内に経口避妊薬や性ホルモン製剤の治療歴のある患者<br>2） 同意取得前 4 週間以内に抗うつ薬の治療歴のある患者<br>3） 同意取得前 4 週間以内に抗不安薬の治療歴のある患者<br>4） 同意取得前 4 週間以内に睡眠薬の治療歴のある患者<br>5） 同意取得前 4 週間以内に利尿薬の治療歴のある患者<br>6） 同意取得前 4 週間以内に PMS に対して効果が報告されている薬剤（OTC：プレフェミンなど、サプリメント含む）による治療歴のある患者<br>7） 重篤な精神疾患を有する患者

	8） 重篤な合併疾患（婦人科疾患、肝疾患、腎疾患、心疾患、血液疾患、代謝性疾患又は悪性腫瘍など）を有する患者<br>9） 妊娠または妊娠している可能性がある、妊娠を希望する患者<br>10）授乳婦である患者<br>11）漢方薬にアレルギーがある患者<br>12）同意取得前 3 ヶ月以内に治験、他の臨床研究に参加していた患者<br>13）同意取得前 4 週間以内に研究薬を含む漢方薬による治療を受けた患者<br>14）その他、医師により対象として不適当と判断された患者
目標症例数	50 例（研究薬投与群：25 例、経過観察群：25 例）
研究薬	TJ-24 ツムラ加味逍遥散エキス顆粒（医療用）
研究期間	2022 年 4 月（認定臨床研究審査委員会承認後）～2025 年 3 月

0.2.　略語及び用語の定義

略語	英語	日本語
ACOG	American College of Obstetricians and Gynecologists	米国産婦人科学会
MDQ	Menstrual Distress Questionnaire	月経随伴症状日本語版
PMS	Premenstrual syndrome	月経前症候群
WPAI	Work Productivity and Activity Impairment Questionnaire	労働生産性評価

目次

0. 研究概要 ...1
0.1. 研究実施計画の概要...1
0.2. 略語及び用語の定義...3
1. 研究の背景と根拠...7
1.1. 月経前症候群の病態及び疫学...7
1.2. 月経前症候群の治療法...7
1.3. 研究計画の根拠...7
1.4. 本研究の意義...9
2. 目的 ...10
3. 評価項目 ...10
3.1. 主要評価項目...10
3.2. 副次評価項目...10
4. 研究対象者 ...10
4.1. 選択基準 ...10
4.2. 除外基準 ...11
5. 研究対象者に説明して同意を得る方法...11
5.1. 説明文書・同意文書の作成及び改訂...11
5.2. 研究対象者への説明と同意取得...12
6. 症例登録／割付方法...13
6.1. 登録手順 ...13
6.2. 割付方法 ...13
7. 研究の方法 ...13
7.1. 研究デザイン...13
7.2. 研究実施手順...14
7.3. 研究薬の用法用量及び投与期間...16
7.4. 研究薬の用量変更及び休薬方法...16
7.5. 併用薬／療法及び飲食物に関する規定...16
7.6. 研究対象者の研究参加予定期間...16
8. 研究薬の管理 ...17
8.1. 研究薬 ...17
8.2. 研究薬の管理...17
9. 中止基準 ...18
10. 観察／検査項目及びスケジュール...19
10.1. 検査／観察項目...19

10.2. 実施スケジュール......22
11. 有害事象の評価／報告......24
11.1. 有害事象及び副作用の定義......24
11.2. 有害事象の報告......25
11.3. 定期疾病等報告......27
11.4. 医薬品等製造販売業者への情報提供......27
12. 研究の終了、中止、中断......27
12.1. 研究の終了......27
12.2. 研究の中止、中断......27
13. 目標症例数及び研究期間......28
13.1. 目標症例数......28
13.2. 研究実施期間......28
14. 統計学的考察......28
14.1. 解析対象集団......28
14.2. 解析方法......28
14.3. 症例数の設定根拠......29
15. 原資料等の直接閲覧......29
15.1. 原資料の範囲......29
15.2. 症例報告書中の原データとすべき項目の特定......30
15.3. 原資料等の直接閲覧の保証......30
16. 品質管理・品質保証......30
16.1. モニタリング......30
16.2. モニタリングの方法......30
17. 倫理的事項......32
17.1. 遵守すべき諸規則......32
17.2. 実施医療機関の管理者への定期報告......32
17.3. 個人情報とプライバシーの保護......32
17.4. 臨床研究に参加することによる利益......33
17.5. 臨床研究に参加することによる安全性及び不利益への配慮......33
17.6. 研究実施後の対応......33
18. 研究の費用負担......33
18.1. 資金源及び財政上の関係......33
18.2. 研究に係る費用と研究対象者負担......34
18.3. 健康被害に対する補償......34
19. 研究実施計画書の承認、改訂、逸脱......34
19.1. 研究実施計画書等の承認及び改訂......34

19.2. 研究実施計画書からの逸脱....................35
20. 症例報告書....................35
20.1. EDC（Electronic Data Capture）システム....................35
20.2. ePRO（患者報告アウトカム電子システム）及びアンケート調査....................36
20.3. 症例報告書データの固定と保管....................37
21. 情報の保管／管理....................37
21.1. 情報の保管／管理....................37
21.2. 情報の二次利用....................37
22. 研究の概要及び結果の登録....................37
23. 研究成果の帰属と研究結果の公表....................37
24. 研究組織....................38
24.1. 研究責任医師....................38
24.2. 研究事務局....................38
24.3. データマネジメント担当者....................38
24.4. モニタリング担当者....................38
24.5. 統計解析担当者....................38
24.6. 研究・開発計画支援担当者....................39
24.7. 共同研究者....................39
24.8. 外部委託機関....................39
25. 参考文献....................40

1. 研究の背景と根拠

1.1. 月経前症候群の病態及び疫学

月経前症候群（Premenstrual syndrome：PMS）は、「月経前 3〜10 日間の黄体期の間に続く精神的あるいは身体的症状で、月経発来とともに減退ないし消滅するもの[1)]」とされる。精神的症状として抑うつ、怒りの爆発、易刺激性・いらだち等、身体的症状として乳房緊満感・膨張、腹部膨満感、頭痛等がある[2)]。性成熟期女性において幅広い年齢で発症するといわれる[3)]が、日本では思春期女性にやや多い傾向が報告されている[4)]。2018 年に日本国内で実施されたインターネットでのアンケート調査では、フルタイムで働く女性 2,000 名のうち 66%が現在又は過去に PMS 症状があったことや、PMS や月経随伴症状によって元気な状態と比べてパフォーマンスが半分以下になると回答した人が 45%であったことを報告している[5)]。また、PMS の病因はいまだに解明されていないが、プロゲステロンに対する感受性との関連や、神経伝達物質セロトニンと γ-アミノ酪酸（GABA）の関与が報告されている[6)]。

1.2. 月経前症候群の治療法

産婦人科診療ガイドラインでは、PMS の治療法の第一選択としてカウンセリング・生活指導・運動療法など非薬物療法が挙げられ、症状日誌の記録による疾患の認識と理解を促す認知行動療法が推奨されている。また、薬物治療としては浮腫や乳房緊満感に対する利尿薬（スピロノラクトン）、鎮痛薬、漢方薬の処方の記載がある。低用量エストロゲン・プロゲスチン配合剤（OC/LEP）の選択肢もあるが、日本では PMS に対して未承認である[7)]。欧米では選択的セロトニン再取り込み阻害薬（SSRI）が PMS の第一選択薬であるが[7)]、日本では PMS に対しては保険適応外である。

1.3. 研究計画の根拠

1.3.1. 研究薬の概要

本研究で用いる一般名：加味逍遙散【製品名：ツムラ加味逍遙散エキス顆粒（医療用）】は以下の適応がある：体質虚弱な婦人で肩がこり、疲れやすく、精神不安などの精神神経症状、ときに便秘の傾向のある次の諸症：冷え症、虚弱体質、月経不順、月経困難、更年期障害、血の道症※。用法・用量は通常，成人 1 日 7.5g を 2〜3 回に分割し，食前又は食間に経口投与する。

※婦人にみられる特有の生理現象に関連して起こる精神神経症状を基調とするさまざまな症状を指す。月経、妊娠、出産、産後、更年期など女性のホルモンの変動に伴って現れる精神不安やいらだちなどの精神神経症状および身体症状のことであり[8)]、PMS も含まれる[9)]。

1.3.2. 非臨床試験成績の概要

加味逍遥散における PMS に対する非臨床での薬効薬理、有効性データはない。一方で、更年期障害に対する作用が確認されており、卵巣摘出マウスへのストレス負荷によるペントバルビタールナトリウム誘発睡眠時間短縮が抑制されたことなどが報告されている [10)]。また、正常マウスに対する抗不安作用も報告されており、その作用は GABAa/ベンゾジアゼピン受容体の活性化を介するものであることが推測されている [11)]。

非臨床における安全性に関しては以下の通りである。

- 単回投与毒性試験 [6)]

動物種	投与経路	性別	概略の致死量（g/kg）
SD ラット	経口	雄	＞8
		雌	＞8

- 反復投与毒性試験 [12)]

  SD 系ラットに 125、500、2000 mg/kg/日を 3 ヵ月間経口投与した結果、2000 mg/kg/日投与群の雌雄において軽度の赤血球数の減少が認められた。この変化は 4 週間の休薬により回復した。

- 生殖発生毒性試験

  該当資料なし

- 遺伝毒性 [13)]

  細菌を用いる復帰突然変異試験、哺乳類培養細胞を用いる染色体異常試験及びマウスを用いる小核試験において遺伝毒性は認められなかった。

1.3.3. 臨床試験成績の概要

加味逍遥散の PMS に対する有効性を示すエビデンスは未だない。しかしながら、月経前に日常生活に支障をきたす精神症状が主体となる月経前不快気分障害（Premenstrual dysphoric disorder : PMDD）において漢方薬の処方を希望した患者 26 例に対し、1 日 7.5g を月経周期 6 サイクル期間投与したところ、寛解率は 53.8%（26 例中 14 例）であった。[14)]。

また、加味逍遥散では 40 歳～60 歳の更年期症状を持つ女性に対する二重盲検プラセボ対照無作為化並行群間試験が実施されており、投与後 12 週間で閉経前女性の興奮イライラスコアがプラセボ群に比べて改善している [15)]。

加味逍遥散では使用成績調査等の副作用発現頻度の調査は実施されていないが、重大な副作用として偽アルドステロン症、ミオパチー、肝機能障害、黄疸、腸間膜静脈硬化症、その他の副作用として過敏症、消化器症状が現れる可能性が報告されている [16)]。

1.3.4. 研究計画

本研究は PMS 症状を持つ女性を無作為に研究薬投与群もしくは経過観察群の 2 群に割付けた平行群間比較試験である。評価は研究薬投与後 3 周期に月経前の症状について経過観察群と比較検討をする。対象患者は、ACOG（米国産婦人科学会）の PMS 診断基準への適合、プレ治療期の月経前の MDQ（月経随伴症状問診票）総スコアが 30 点以上、正常な性周期を有することを主な選択基準とする。なお、MDQ のスコア値は既報の PMS 患者データ [17] から妥当性を判断した。主要評価項目は研究薬投与群の 3 周期後の月経前 MDQ の総スコアの変化量を経過観察群と比較検討する。MDQ は、月経周期に伴う心身両面に渡る愁訴を測定する尺度として 1968 年に Moos[17] らにより開発され、日本語版は 1979 年に茅島らによって作成された。本邦では日本産科婦人科學會雜誌 [19] 等において、PMS 症状を評価する質問表として挙げられ数多くの研究で採用されている。また、スイスの Max Zeller Söhne AG 社により開発された月経前治療薬「プレフェミン」の有効性を検討する臨床試験 [20] などでも採用されている。国内外の多くの論文で採用されていることから本研究で主要評価項目と設定した。副次評価項目は 1）月経前、月経中、月経後 MDQ の総スコア、下位尺度の経時変化、2）労働生産性、冷え・肩こり、研究参加後の全体印象、3）スマートフォンアプリ（YaDoc）による睡眠、漢方医学、月経関連症状、4）Fitbit（スマートウォッチ）による睡眠・心拍について、研究薬投与群と経過観察群の群ごと、観察周期ごとに集計し、変化量に関して研究薬投与群と経過観察群の両群間の比較検定を行う。また、患者背景や加味逍遙散の使用目標による効果の違いを探索的に検討するため、患者特性と勤務体系を基準にサブグループを設定し解析を実施する。安全性評価は来院時に身体所見（浮腫の有無など）、血圧・脈拍数の測定を実施し、登録時と研究終了時に血液検査を行う。安全性評価項目は既知の情報より設定した [16]。研究スケジュールは研究対象者の登録後、次の月経が始まるまでをプレ治療期とし、性周期の起点を調整するための期間を設定する。その後、治療期として研究薬投与群は研究薬を月経開始日から服薬開始し、3 周期まで服薬する。研究対象者はプレ治療期に 1 回、服薬後 1 周期ごとに来院し検査・観察を行う。また、第 2、3 周期及び後観察期の第 4 周期の月経開始日に自宅にてオンラインアンケート調査に回答する。さらに、研究参加期間を通じて毎日、スマートフォンアプリ（YaDoc）による日誌の回答、Fitbit（スマートウォッチ）による睡眠評価・心拍評価のデータ取得を行う。評価期間及び評価時点の設定は既報を参考に設定した [15, 21, 22, 23]。

1.4. 本研究の意義

本邦においては、PMS に対して、標準的な治療法が確立しておらず、本研究により加味逍遙散の PMS に対する改善が期待される。本研究では PMS 患者に対する加味逍遙散の有効性と安全性について、アンケート調査、スマートフォンアプリによる日誌、Fitbit（スマートウォッチ）を用いて評価をする。PMS に対する加味逍遙散の効果が認められれば、将来の治療薬の選択肢を広め、多くの性成熟期女性の QOL 向上に寄与することができる。

2. 目的

月経前症候群（PMS）患者を対象に、加味逍遥散投与群と経過観察群を比較し、加味逍遥散の有効性及び安全性を評価する。

3. 評価項目

3.1. 主要評価項目

月経前の Menstrual Distress Questionnaire（MDQ）総スコアのベースライン時から最終評価時までの変化量

3.2. 副次評価項目

1） 月経前、月経中、月経後の MDQ 総スコア、各下位尺度スコアの経時変化
2） 睡眠評価
3） 漢方医学的評価
4） 月経関連症状評価
5） 労働生産性評価
6） 冷え、肩こりスコア
7） 研究参加後の全体印象
8） Fitbit（スマートウォッチ）による睡眠評価・心拍評価
9） 有害事象

4. 研究対象者

以下の選択基準をすべて満たし、除外基準のいずれにも該当しない者を登録する。

4.1. 選択基準

以下の基準をすべて満たす者を対象とする。

1） ACOG の PMS 診断基準に基づき PMS と診断された患者
2） プレ治療期の月経前の MDQ 総スコアが 30 点以上の患者
3） 同意取得時に 18 歳以上、45 歳以下の患者
4） 月経周期が 25 日から 38 日間の規則的な患者
5） 研究薬を経口服用できる患者
6） 受診区分：外来
7） 本研究への文書による参加同意が得られた患者

4.2.　除外基準

以下のいずれかに抵触する者は本臨床研究に組み入れないこととする。

1）　同意取得前 4 週間以内に経口避妊薬や性ホルモン製剤の治療歴のある患者
2）　同意取得前 4 週間以内に抗うつ薬の治療歴のある患者
3）　同意取得前 4 週間以内に抗不安薬の治療歴のある患者
4）　同意取得前 4 週間以内に睡眠薬の治療歴のある患者
5）　同意取得前 4 週間以内に利尿薬の治療歴のある患者
6）　同意取得前 4 週間以内に PMS に対して効果が報告されている薬剤（OTC : プレフェミンなど、サプリメント含む）による治療歴のある患者
7）　重篤な精神疾患を有する患者
8）　重篤な合併疾患（婦人科疾患、肝疾患、腎疾患、心疾患、血液疾患、代謝性疾患又は悪性腫瘍など）を有する患者
9）　妊娠または妊娠している可能性がある、妊娠を希望する患者
10）授乳婦である患者
11）漢方薬にアレルギーがある患者
12）同意取得前 3 ヶ月以内に治験、他の臨床研究に参加していた患者
13）同意取得前 4 週間以内に研究薬を含む漢方薬による治療を受けた患者
14）その他、医師により対象として不適当と判断された患者

5.　研究対象者に説明して同意を得る方法

5.1.　説明文書・同意文書の作成及び改訂

研究責任医師は、研究対象者から本臨床研究への参加の同意を得るために用いる説明文書・同意文書等を作成する。また、本研究期間中に、研究対象者の同意に影響を与え得る新たな重要な情報、あるいは本研究に継続して参加するかどうかについての研究対象者の意思に影響を与える可能性のある情報が得られた場合にはこれを改訂する。作成又は改訂した説明文書・同意文書は、あらかじめ認定臨床研究審査委員会で審査した上で使用する。なお、説明文書には、以下の項目を含む。

1）　実施する臨床研究の名称、当該臨床研究の実施について実施医療機関の管理者の承認を受けている旨及び厚生労働大臣に実施計画を提出している旨
2）　実施医療機関の名称並びに研究責任医師の氏名及び職名（特定臨床研究を多施設共同研究として実施する場合にあっては、研究代表医師の氏名及び職名並びに他の実施医療機関の名称並びに当該実施医療機関の研究責任医師の氏名及び職名を含む。）
3）　臨床研究の対象者として選定された理由
4）　臨床研究の実施により予期される利益及び不利益
5）　臨床研究への参加を拒否することは任意である旨

6) 同意の撤回に関する事項
7) 臨床研究への参加を拒否すること又は同意を撤回することにより不利益な取扱いを受けない旨
8) 臨床研究に関する情報公開の方法
9) 臨床研究の対象者又はその代諾者の求めに応じて、研究実施計画書その他の臨床研究の実施に関する資料を入手又は閲覧できる旨及びその入手又は閲覧の方法
10) 臨床研究の対象者の個人情報の保護に関する事項
11) 試料等の保管及び廃棄の方法
12) 医薬品等製造販売業者等の関与に関する状況
13) 苦情及び問合せへの対応に関する体制
14) 臨床研究の実施に係る費用に関する事項
15) 他の治療法の有無及び内容並びに他の治療法により予期される利益及び不利益との比較
16) 臨床研究の実施による健康被害に対する補償及び医療の提供に関する事項
17) 臨床研究の審査意見業務を行う認定臨床研究審査委員会における審査事項その他当該臨床研究に係る認定臨床研究審査委員会に関する事項
18) その他臨床研究の実施に関し必要な事項

5.2. 研究対象者への説明と同意取得

研究責任医師又は研究分担医師は、研究開始に先立ち、認定臨床研究審査委員会で承認された説明文書・同意文書を用いて候補となった研究対象者本人に十分に説明した上で、研究参加について自由意思による同意を研究対象者本人より文書で得る。同意を得る際には、研究対象者に研究に参加するか否かを判断するのに十分な時間と質問する機会を設け、質問に十分に答える。説明した研究責任医師又は研究分担医師、並びに研究対象者は、同意文書に署名し、各自日付を記入する。同意文書は実施医療機関で保管し、説明文書及び同意文書の写しは研究対象者に渡す。

本研究期間中に、研究対象者の同意に影響を与え得る新たな重要な情報、あるいは本研究に継続して参加するかどうかについての研究対象者の意思に影響を与える可能性のある情報が得られた場合は、研究責任医師は速やかに当該情報に基づき説明文書等を改訂し、認定臨床研究審査委員会の承認を得たのち、研究責任医師又は研究分担医師は当該情報を速やかに研究対象者に説明して本研究への継続参加について自由意思による同意を文書により得て、その同意文書の写しを研究対象者に渡す。また、同意後も随時同意の撤回ができ、撤回による不利益を受けない。

6. 症例登録／割付方法

6.1. 登録手順

研究責任医師又は研究分担医師は、以下の手順で研究対象者の登録を行う。

1） 研究対象者より文書による同意を取得し、研究対象者が選択基準を満たし、除外基準に抵触していないことを確認する。

2） 研究責任医師が保管する対応表に、同意取得日及び研究対象者と研究対象者識別コードを対応させるために必要な事項を記載して、その記述単体で特定の研究対象者を直ちに判別できる記述等を匿名化する。

3） 症例登録は、研究支援クラウドサービス（APS-Cloud）（以下、TUH-RSS）による Web 登録とする。研究責任医師又は研究分担医師は、Web サイト上で適格性の判断に必要な研究対象者背景について入力する。なお、情報の入力・送信については、研究責任医師の指導・監督のもと、研究協力者が行うことができる。

4） Web の適格性判定で適格と判定された場合は、登録番号が発行される。

- 研究責任医師又は研究分担医師は、研究対象者の登録がなされるまで研究を開始してはならない。
- 一度登録された研究対象者を登録取り消し（データベースから抹消）できない。重複登録の場合は、いかなる場合も初回の登録情報を採用する。
- 誤登録、重複登録が判明した際には速やかにデータセンターに連絡する。

データセンター

鳥取大学医学部附属病院　新規医療研究推進センター

担当者　砂田　寛司

住所：鳥取県米子市西町 36-1

TEL：0859-38-6946／FAX：0859-36-6947

6.2. 割付方法

研究対象者は、プレ治療期の評価で MDQ 80 点以上を層別因子として、研究薬投与群あるいは経過観察群のいずれかに 1：1 の割合でランダムに割り付ける。ランダム割付に際しては、ブロックランダム化法を用いる。

7. 研究の方法

7.1. 研究デザイン

本研究は、非盲検、無作為化、臨床研究である。なお、本研究には研究薬である加味逍遥散を投与する研究薬投与群と研究薬を投与しない経過観察群を設定する。

7.2.　研究実施手順

本研究は、プレ治療期、治療期及び後観察期からなる。研究薬投与開始の月経周期（第 1 周期）を起点にして、－1 周期（プレ治療期）に同意を取得し、選択基準を満たし、除外基準に抵触しないことを確認後、登録及び割付（研究薬投与群 : 経過観察群＝1 : 1）を行う。－1 周期（プレ治療期）は、当該月経周期の月経開始日（＋14 日以内）に来院する。その後、第 1 周期の月経開始日から治療期に移行し、研究薬投与群は研究薬の投与、及び経過観察群は経過観察を開始する。治療期は、第 3 周期までとする。各月経周期には、3 つの期間（月経中、月経後、月経前）を設定し、それぞれ月経中 : 月経周期の経血期間、月経前 : 来院日の月経開始日を 1 日目として－7～−1 日目、月経後 : 月経前と月経中を除いた期間と定義する。治療期の第 2、3 周期及び後観察期の第 4 周期の月経開始日（＋14 日以内）に来院し、検査、観察を行う。また、第 2、3 周期及び後観察期の第 4 周期の月経開始日に自宅にてオンラインアンケート調査を実施する。さらに、研究参加期間を通じて毎日（1 日 1 回、可能な限り同じ時間）スマートフォンアプリ（YaDoc）を用いて、睡眠評価、漢方医学的評価、月経関連症状評価を行う。治療期の就寝時には、毎日 Fitbit（スマートウォッチ）を装着し、睡眠並びに心拍を評価する。治療期の途中で中止する場合には、来院して中止検査を行う。

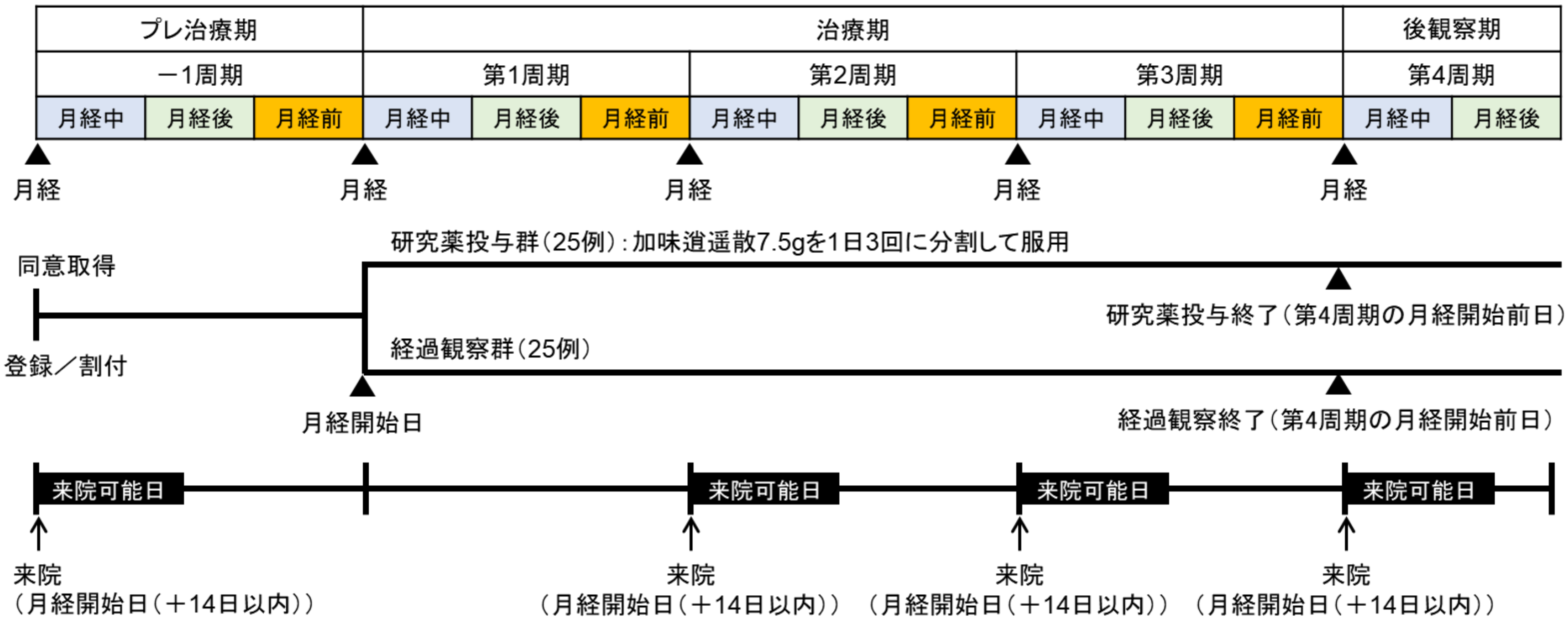
プレ治療期
治療期
後観察期
ー1周期
第1周期
第2周期
第3周期
第4周期
月経中
月経後
月経前
月経
研究薬投与群（25例）：加味逍遥散7.5gを1日3回に分割して服用
同意取得
登録／割付
研究薬投与終了（第4周期の月経開始前日）
経過観察群（25例）
月経開始日
経過観察終了（第4周期の月経開始前日）
来院可能日
来院
（月経開始日（＋14日以内））

図 7-1　研究計画

7.3. 研究薬の用法用量及び投与期間

研究薬は 1 日 7.5 g を 3 回に分割（1 回 2.5 g）し、食前又は食間に経口投与する。飲み忘れた場合、食後投与も可とする。投与は第 1 周期から第 3 周期まで行う。

7.4. 研究薬の用量変更及び休薬方法

本研究では、研究薬の用法用量の変更は禁止する。

7.5. 併用薬／療法及び飲食物に関する規定

7.5.1. 併用禁止薬

以下に示す薬剤・療法は、同意取得以降、治療期終了まで併用を禁止する。

1） 経口避妊薬や性ホルモン製剤
2） 睡眠薬、抗不安薬、抗うつ薬
3） 利尿薬
4） PMS に対して効果が報告されている薬剤（OTC：プレフェミン、サプリメント含む）
5） 日本で未承認の治験薬、他の研究薬又は治療法
6） 研究薬以外の漢方薬

7.5.2. 併用制限薬

同意取得 3 ヶ月以上前から服用している薬剤（サプリメント含む）及び治療法（運動療法、ヨガ含む）は、継続可能とするが、同意取得以降、治療期終了まで、用法・用量並びに使用方法・実施方法を変更してはならない。なお、鎮痛剤についても頓用を含めて併用可能であるが、同意取得以降、治療期終了まで、用法・用量を変更してはならない。やむを得ず変更した場合は、変更の内容を症例報告書に記載する。

添付文書の併用注意に該当する薬剤（カンゾウ含有製剤、グリチルチリン酸及びその塩類を含有する製剤）は併用可能であるが、加味逍遥散と相互作用を起こす可能性があるため、研究薬投与期間中は併用に注意する。

7.6. 研究対象者の研究参加予定期間

研究対象者の予定参加期間は、プレ治療期（－1 周期）、治療期（3 周期）及び後観察期となる。予定参加日数は、月経周期による異なる。

8. 研究薬の管理

8.1. 研究薬

一般名	：加味逍遙散
化学名	：なし
含量及び剤型	：本品 7.5g 中、下記の割合の混合生薬の乾燥エキス 4.0g を含有する。<br>日局サイコ（柴胡）…3.0g<br>日局シャクヤク（芍薬）…3.0g<br>日局ソウジュツ（蒼朮）…3.0g<br>日局トウキ（当帰）…3.0g<br>日局ブクリョウ（茯苓）…3.0g<br>日局サンシシ（山梔子）…2.0g<br>日局ボタンピ（牡丹皮）…2.0g<br>日局カンゾウ（甘草）…1.5g<br>日局ショウキョウ（生姜）…1.0g<br>日局ハッカ（薄荷）…1.0g<br>添加物　日局ステアリン酸マグネシウム、日局乳糖水和物
剤型	：顆粒剤
貯法	：薬の品質を保つため、できるだけ湿気をさけ、直射日光のあたらない涼しい所に保管すること。
製造番号	：製造時に記載

8.2. 研究薬の管理

8.2.1. 研究薬の納入手順

研究薬は、認定臨床研究審査委員会での承認後に、研究薬提供者との契約書で定める研究開始日以降に以下の手順で提供される。

1） 研究薬提供者は、納入希望日に実施医療機関へ「研究薬提供書」を添えて研究薬を納入する。
2） 研究薬管理者から指示を受けた者（以下、「研究薬管理者等」という）は、研究薬納入時までに「研究薬保管・管理表」を作成する。
3） 研究薬管理者等は、納入された研究薬の数量と汚損、破損の有無と「研究薬提供書」を確認する。
4） 研究薬管理者等は、押印又は署名した「研究薬受領書」を作成し、研究薬提供者に提出する。また、研究責任医師は、「研究薬提供書」を保存する。
5） 研究薬管理者等は、研究薬の受領に問題ないことを確認した後、必要項目を「研究薬保管・管理表」に記載し、研究薬の出納を管理する。

8.2.2. 研究薬の保管

研究薬管理者等は、貯蔵方法に従い研究薬を適切に保管する。

8.2.3. 研究薬の管理

研究薬管理者等は、「未使用研究薬*」と「使用済研究薬**」を区別して管理する。また、研究薬は、本研究以外の目的（他の臨床研究、動物実験、基礎実験等）に使用してはならない。

*未使用研究薬：出庫されていない薬剤（処方されていない薬剤）をいう。
**使用済研究薬：出庫された薬剤（未服用により返却された薬剤を含む）をいう。

8.2.3.1. 研究薬の返却手順

「未使用研究薬」「使用済研究薬」は、以下の手順で研究薬提供者へ返却する。

1) 研究薬管理者等は、未使用研究薬を原則、研究が終了するまでの期間、他の研究薬と区分して保管する。使用済研究薬は原則、研究が終了するまでの期間保管し、廃棄は行わない。使用済研究薬の外箱は順次廃棄可能とする。
2) 研究薬管理者等は、研究薬提供者へ記名、押印又は署名した未使用研究薬返却書、未使用研究薬、使用済研究薬返却書、使用済研究薬と「研究薬保管・管理表（写し）」を提供する（未使用研究薬・使用済研究薬が 0 包の場合も薬剤数、箱数を 0 と記入した未使用研究薬返却書・使用済研究薬返却書を作成する）。

8.2.4. 研究薬の出庫及び指導

1) 研究薬管理者等は、研究責任医師等の処方指示に従って研究薬を出庫する。その際、「研究薬保管・管理表」に必要事項を記載する。
2) 研究薬管理者等は、研究薬の出庫にあたり、研究実施計画書からの逸脱や疑義が判明した場合は、速やかに研究責任医師等にその旨を連絡し、解決された後に研究薬を出庫する。
3) 研究薬管理者等は、研究対象者に、服用の方法、服用を忘れたときの対処法、研究薬の保管方法、研究薬の返却（紛失・廃棄時の報告含む）等について、適切に指導する。
4) 研究薬管理者等は、研究対象者に空のアルミのヒートシールは順次適切に廃棄してよいことを説明する。

9. 中止基準

治療期開始後に以下の事例が発生した場合は、研究責任医師又は研究分担医師は研究を中止し、中止時の検査を実施する。なお、有害事象の発現などの安全性に問題が生じて中止

した場合、研究責任医師又は研究分担医師は、直ちに適切な処置を行い、可能なかぎり回復まで追跡調査を行う。

1） 研究対象者から研究参加の辞退の申し出や同意の撤回があった場合
2） 登録後に適格性を満たさないことが判明した場合
3） 原疾患の悪化のため、研究の継続が好ましくないと判断された場合
4） 合併症の増悪により研究の継続が困難な場合
5） 有害事象により研究の継続が困難な場合
6） 転居など研究対象者の都合で研究の継続が不可能な場合
7） 妊娠が判明した場合
8） 研究全体が中止された場合
9） その他の理由により、研究責任医師又は研究分担医師が研究を中止することが適当と判断した場合

10. 観察／検査項目及びスケジュール

10.1. 検査／観察項目

10.1.1. 研究対象者背景、原疾患情報、人口統計学的及びその他の特性

プレ治療期に以下のデータを収集する。

1） 研究対象者背景：同意取得日、同意取得時の年齢、アレルギー歴、初経年齢、勤務体系
2） 原疾患情報：診断名、診断日、月経周期
3） 患者特性（肩こり、頭痛、めまい、灼熱感、発作性発汗、冷え、便秘、肌荒れ、疲労感、食生活、運動、飲酒、睡眠）
4） 既往歴、合併症

既往歴は、同意取得前に消失又は終了した臨床的に問題となる疾患又は症状で、安全性評価に関連のあるものを調査する。合併症とは、同意取得時に有する症状、又は疾患のことをいう。登録前の観察／検査で認められた臨床的に問題となる異常所見は、研究責任医師又は研究分担医師の判断により合併症とする。

5） 前治療薬／療法

治療期開始までの間に投与又は実施されるすべての薬剤又は治療のことである。治療期開始時までの間に医師が処方した薬剤又は研究対象者が購入した市販薬を含む薬剤／療法を確認し、記録する。なお、原疾患に対する治療薬／療法は、すべて収集する。

10.1.2. 身体所見

身体所見は、プレ治療期、第 2、3、4 周期の月経開始日（＋14 日以内）又は中止時に行

う。身体所見では、浮腫の有無の評価も行う。治療期開始後の診察結果については、治療期開始前と比較して臨床的に有意な異常の有無を評価する。臨床的に有意な異常の場合は、有害事象として報告する。

10.1.3. 身長、体重、バイタルサイン、BMI

身長、体重及び BMI は、プレ治療期に行い、小数点第 1 位までを記録する。また、バイタルサインは、プレ治療期、第 2、3、4 周期の月経開始日（＋14 日以内）又は中止時に行い、血圧、脈拍数を測定する。血圧、脈拍数は、5 分間以上安静にした後に座位で測定する。治療期開始後の結果については、治療期開始前と比較して臨床的に有意な異常の有無を評価する。臨床的に有意な異常の場合は、有害事象として報告する。

10.1.4. 血液検査

血液検査（AST、ALT、γ-GTP、ナトリウム、カリウム）は、プレ治療期、第 4 周期の月経開始日（＋14 日以内）又は中止時に行う。治療期開始後の結果については、治療期開始前と比較して臨床的に有意な異常の有無を評価する。臨床的に有意な異常の場合は、有害事象として報告する。本研究から得られた試料（血液）は、測定後、実施医療機関の手順に従って速やかに廃棄する。

10.1.5. Menstrual Distress Questionnaire（MDQ）

Menstrual Distress Questionnaire（MDQ）は、月経周期に伴う心身両面に渡る愁訴（月経随伴症状）を測定する尺度で、「痛み」、「集中力の低下」、「行動の変化」、「自律神経失調」、「水分貯留」、「否定的情緒」、「気分の高揚」、「コントロール」の 8 つの尺度から構成される。評価は、月経周期を「月経中」、「月経後」、「月経前」の 3 つの期間に分けて行う。プレ治療期は、来院時に直前の月経周期の「月経中」、「月経後」、「月経前」の状態を思い起こして、それぞれの期間についてアンケートで評価する。第 2、3、4 周期は、月経開始日に、直前の月経周期の「月経中」、「月経後」、「月経前」の状態を思い起こして、それぞれの期間について、自宅にてオンラインアンケートで評価する（表 10-1）。中止時については、直近の該当する期間の状態を評価する。

表 10-1　評価月経周期

評価実施日	評価対象の月経周期	評価方法
−1 周期の来院時	−2 周期	紙媒体（病院）
第 2 周期の月経開始日	第 1 周期	オンライン（自宅）
第 3 周期の月経開始日	第 2 周期	オンライン（自宅）
第 4 周期の月経開始日	第 3 周期	オンライン（自宅）

来院日の直前の月経周期の経血期間を「月経中」、来院日の月経開始日を 1 日目として、−7～−1 日目を「月経前」、及び直前の月経周期の月経前と月経中を除いた期間を「月経後」とする（図 10−1）。

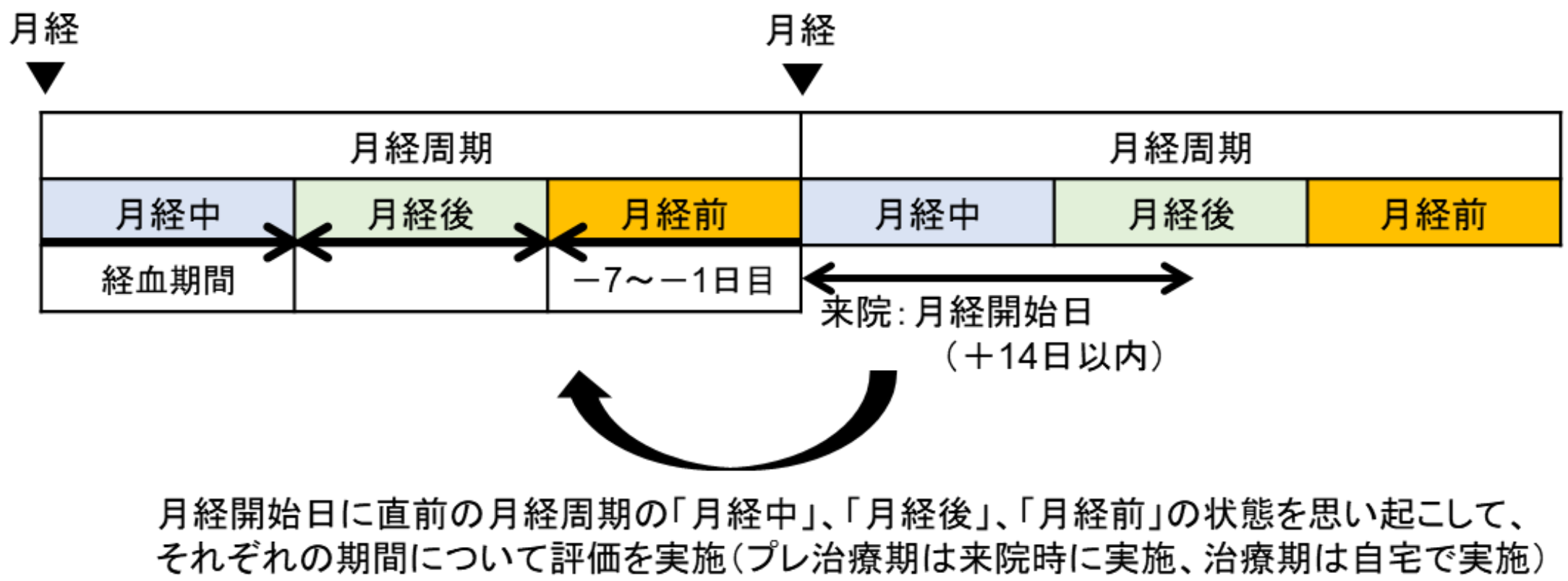

図 10-1　MDQ の評価方法

10.1.6. 労働生産性評価

Work Productivity and Activity Impairment Questionnaire（WPAI）を用いて、健康問題で仕事を休んだ時間、健康問題以外で仕事を休んだ時間、働いた時間、健康問題で労働生産性に影響した程度、健康問題で仕事以外の日常活動に影響した程度を評価する。プレ治療期は、来院時に来院日前 7 日間の状態を評価する。治療期及び後観察期は、第 2、3、4 周期の月経開始日又は中止時に、評価日前 7 日間の状態を自宅にてオンラインアンケートで評価する。

10.1.7. 冷えスコア

プレ治療期は来院時に評価する。第 2、3、4 周期は、月経開始日に自宅にてオンラインアンケートで、冷えに関する自覚症状に関して、直前の月経周期の「月経前」の状態を思い起こして 0～10 の 11 段階で評価する。なお、中止時については、直近の該当する期間の状態を評価する。

10.1.8. 肩こりスコア

プレ治療期は来院時に評価する。第 2、3、4 周期の月経開始日に自宅にてオンラインアンケートで、肩こりに関する自覚症状に関して、直前の月経周期の「月経前」の状態を思い起こして 0～10 の 11 段階で評価する。なお、中止時については、直近の該当する期間の状態を評価する。

10.1.9. 研究参加後の全体印象

第 4 周期の月経開始日又は中止時に、自宅にてオンラインアンケートで、PMS 症状に対する研究参加後の全体的な印象を以下のリッカートスケールによる 7 段階で評価する。

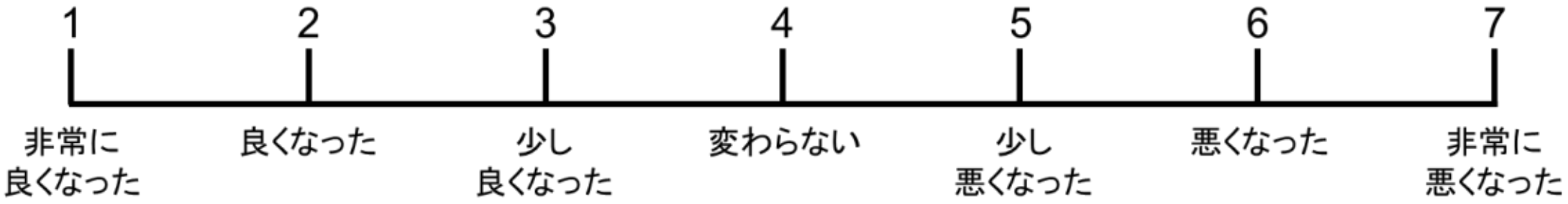

10.1.10. スマートフォンアプリ（YaDoc）による症状評価と月経日の記録

スマートフォンアプリ（YaDoc）を用いて、漢方医学的評価（イライラ、情緒不安定など）、月経関連評価（お腹の張り、疲れやすさ）、睡眠の状態評価（睡眠に対する満足度、日中の眠気などの状態）を 0～10 の 11 段階で実施する。また、月経日及び月経中か否かの記録も行なう。評価は、登録日から開始し、第 3 周期の終了時（第 4 周期の月経 1 日目の前日）まで、毎日（1 日 1 回、可能な限り同じ時間）行う。なお、登録日から開始できない場合は、治療期開始前、できるだけ速やかにデータ収集・評価を開始する。

10.1.11. Fitbit（スマートウォッチ）による評価

Fitbit（スマートウォッチ）を用いて、睡眠時の心拍変動、心拍数、睡眠時間、睡眠状態に関する情報を収集する。評価は、登録時から開始し、第 3 周期の終了時（第 4 周期の月経 1 日目の前日）まで、毎日就寝時に Fitbit（スマートウォッチ）を装着して評価を実施する。なお、登録日から開始できない場合は、治療期開始前、できるだけ速やかにデータ収集・評価を開始する。なお、本システムでは、睡眠時皮膚温、ストレススコア、血中酸素データも同時に取得されるが、本研究には使用しない。当該データについては、将来の貴重なデータとなるため、他のデータとともに保管をし、必要に応じて、別の目的の研究で二次利用することがある。

10.1.12. 併用薬・併用療法

併用薬・併用療法とは、有害事象に関わるもので、治療期及び後観察期に実施される治療に関する情報を収集する。なお、治療期開始後に発生した因果関係の否定できない有害事象のための治療は、併用薬・併用療法として収集する。

10.2. 実施スケジュール

本研究は、プレ治療期、治療期及び後観察期の 3 つの期間からなる。観察・検査・評価の実施スケジュールを表 10-2 に示す。

表 10-2　実施スケジュール

●：必須　▲：可能な限り実施

	プレ治療期	治療期			後観察期	中止時
月経周期	－1周期[1]	第1周期[2]	第2周期	第3周期	第4周期[3]（終了時）	
来院タイミング	月経開始日	―	月経開始日	月経開始日	月経開始日	中止時
許容範囲	＋14日	―	＋14日	＋14日	＋14日	速やかに
来院	●		●[4]	●[4]	●[4]	●[5]
同意取得／登録	●					
研究対象者背景	●					
原疾患情報	●					
身長、体重、BMI	●					
前・併用薬／療法	←	―	―	―	―	→
研究薬投与[6]		←	―	→		
安全性評価						
身体所見	●		●	●	●	●
血圧、脈拍数	●		●	●	●	●
血液検査	●				●	●
有害事象		←	―	―	―	→
アンケートによる評価[7]						
MDQ	●		●	●	●	●[8]
労働生産性評価	●		●	●	●	●
冷えスコア	●		●	●	●	●[8]
肩こりスコア	●		●	●	●	●[8]
研究参加後の全体印象					●	●
スマートフォンアプリ（YaDoc）による評価[9]						
睡眠評価・漢方医学的評価・月経関連症状評価	▲[10]	←	―	→		
Fitbit（スマートウォッチ）による評価[11]						
睡眠評価・心拍評価	▲[10]	←	―	→		

[1] 研究薬投与開始の月経周期（第 1 周期）を起点にして、－1 周期（プレ治療期）に同意を取得して適格確認を行う。

[2] 第 1 周期の月経開始 1 日目から治療期を開始する。

[3] 第 4 周期の月経開始日（＋14 日以内）に来院し、終了時検査を実施する。

[4] 各周期の月経開始日（＋14 日以内）に来院する。
[5] 中止時に来院し、規定の検査・観察を行う。
[6] 研究薬投与群のみ。研究薬は、第 1 周期の 1 日目から開始し、第 3 周期の終了時（第 4 周期の月経 1 日目の前日）まで継続する。
[7] 治療期の第 2、3、4 周期は、月経開始日に自宅にてオンラインアンケートで評価する。
[8] 中止時については、直近の該当する期間の状態を評価する。
[9] 第 3 周期の終了時（第 4 周期の月経 1 日目の前日）まで、毎日（1 日 1 回、可能な限り同じ時間）スマートフォンアプリ（YaDoc）で評価を実施する。
[10] 可能な限り登録日からデータの収集・評価を開始する。登録日から開始できない場合は、治療期開始前、できるだけ速やかにデータ収集・評価を開始する。
[11] 第 3 周期の終了時（第 4 周期の月経 1 日目の前日）まで、毎日就寝時に Fitbit（スマートウォッチ）を装着して評価を実施する。

11. 有害事象の評価／報告

11.1. 有害事象及び副作用の定義

11.1.1. 有害事象及び副作用

有害事象とは、医薬品（研究薬を含む）が投与された患者又は研究対象者に生じたあらゆる好ましくない医療上のできごとで、必ずしも当該医薬品（研究薬を含む）の投与との因果関係が明らかなもののみを示すものではない。つまり有害事象とは、医薬品（研究薬を含む）が投与された際に起こる、あらゆる好ましくない、あるいは意図しない徴候（臨床検査値の異常変動を含む）、症状又は病気のことであり、当該医薬品（研究薬を含む）との因果関係の有無は問わない。有害事象のうち、研究薬との因果関係が否定できない場合（不明を含む）を副作用として取り扱う。

なお、有害事象のうち、研究の実施に起因するものと疑われる場合を疾病等として取り扱い、認定臨床研究審査委員会への報告対象とする。

11.1.2. 重篤な有害事象

重篤な有害事象とは、「11.1.1　有害事象及び副作用」で規定した有害事象のうち、以下に該当するものをいう。

1. 死に至るもの
2. 生命を脅かすもの
3. 治療のため入院又は入院期間の延長が必要となるもの
4. 永続的又は顕著な障害・機能不全に陥るもの
5. 先天異常を来すもの
6. その他、即座に生命を脅かしたり、入院には至らなくとも、研究対象者を危機

にさらしたり、上記 1〜5 のような結果に至らぬように処置を必要とするような重大な事象

11.2. 有害事象の報告

11.2.1. 有害事象の報告

有害事象の収集は、研究対象者への治療期開始時点より開始し、最終来院時まで継続して行う。

研究責任医師又は研究分担医師は、研究対象者の来院の際、自覚症状の発現の有無、臨床検査値等の異常変動の有無を確認する。

有害事象については、症例報告書に有害事象の名称、発現日、重症度、重篤性、研究薬との関連性と判定理由、研究薬に対する処置、転帰及び転帰日などを記録する。

11.2.2. 疾病等の報告

11.2.2.1. 疾病等の報告手順

1) 研究責任医師は、本研究に起因すると疑われる「表 11-1　認定臨床研究審査委員会への報告対象となる疾病等」に示す疾病等の発現を知った時は、「特定臨床研究における医薬品疾病等報告書の提出に関する手順書」に従い、法令の定める報告期限内に「医薬品の疾病等報告書（統一書式 8）及び「詳細記載様式」の第一報を作成し、認定臨床研究審査委員会及び実施医療機関の管理者に報告すると共に、株式会社ツムラに報告する。

表 11-1　認定臨床研究審査委員会への報告対象となる疾病等

予測性	重篤性	報告期限（※1）
未知・既知	死亡	15 日報告
未知	死亡につながるおそれのある疾病等（※2）	
	下記①から⑤までの疾病等（感染症を除く）の発生（※2）<br>① 治療のために医療機関への入院又は入院期間の延長が必要とされる疾病等<br>② 障害<br>③ 障害につながるおそれのある疾病等<br>④ ①から③まで並びに死亡及び死亡につながる恐れのある疾病等に準じて重篤である疾病等<br>⑤ 後世代における先天性の疾病又は異常	
	感染症による疾病等の発生	
既知	感染症による死亡	

	感染症による上記①から⑤までの疾病等の発生	
既知	上記①から⑤までの疾病等の発生	30 日報告

（※1）認定臨床研究審査委員会及び研究代表医師、実施医療機関の管理者あるいは厚生労働大臣への報告期限

（※2）当該特定臨床研究に用いた医薬品等の添付文書又は容器若しくは被包に記載された使用上の注意（以下「使用上の注意等」という。）から予測することができないもの又は当該医薬品等の使用上の注意等から予測することができるものであって、その発生傾向を予測することができないもの若しくはその発生傾向の変化が保健衛生上の危害の発生若しくは拡大のおそれを示すもの。

2) 研究責任医師は、本研究の実施によるものと疑われるものであって、予測できない死亡、及び死亡につながるおそれがある疾病等又は重篤と判断する事項を知った時は、速やかに実施医療機関の管理者へ報告する。その際の報告方法は問わない。

3) 研究責任医師は、「表 11-1　認定臨床研究審査委員会への報告対象となる疾病等」に示す疾病等の新たな情報を得た場合又は疾病等の転帰が変更となった場合には、「医薬品の疾病等報告書（統一書式 8）」及び「詳細記載様式」の追加報を作成し、認定臨床研究審査委員会及び実施医療機関の管理者に報告する。

＜研究責任医師連絡先＞

鳥取大学医学部附属病院　女性診療科　谷口　文紀

〒683-8504　鳥取県米子市西町36-1

電話：0859-38-6647／FAX：0859-38-6649

＜休日・夜間連絡先＞

鳥取大学医学部附属病院　女性診療科病棟

電話：0859-38-6913

11.2.2.2. 厚生労働省への報告手順

1) 研究責任医師は、本研究に起因すると疑われる「表 11-1　認定臨床研究審査委員会への報告対象となる疾病等」に示す疾病等のうち、「未知」に該当する疾病等の発現を知った時は、「11.2.2.1.　疾病等の報告手順」の手続きを行うとともに、jRCT（臨床研究実施計画・研究概要公開システム）の疾病等報告にて第一報を作成する。

2) 研究責任医師は、厚生労働大臣に報告の対象となる事項について、新たな情報を得た場合又は疾病等の転帰が変更となった場合には、jRCT にて追加報を作成する。

3）　研究責任医師は、「表 11-1　認定臨床研究審査委員会への報告対象となる疾病等」で示す法令の定める報告期限内に jRCT にて報告する。

11.3. 定期疾病等報告

1）　「表 11-1　認定臨床研究審査委員会への報告対象となる疾病等」に示す疾病等以外の本研究の実施に起因するものと疑われる疾病等については、「定期報告書（統一書式 5）」による定期報告の対象とする。

2）　研究責任医師は、原則として実施計画を厚生労働大臣に提出した日から起算して 1 年ごとに、また、当該期間満了後 2 ヶ月以内に定期報告書を作成し、認定臨床研究審査委員会及び実施医療機関の管理者に報告する。

11.4. 医薬品等製造販売業者への情報提供

研究責任医師は、医薬品等疾病等報告の対象となる研究薬等の製造販売をし、又はしようとする医薬品等製造販売業者から情報を求められた場合には情報提供を行う。

12. 研究の終了、中止、中断

12.1. 研究の終了

研究期間の終了及びすべての症例報告書の確定をもって、研究を終了する。研究責任医師は、総括報告書及びその概要を「終了通知書（統一書式 12）」に添えて認定臨床研究審査委員会に提出し、意見を聴く。研究責任医師は、総括報告書及びその概要について、認定臨床研究審査委員会の意見を聴いた後、遅滞なく実施医療機関の管理者に提出する。

12.2. 研究の中止、中断

研究責任医師は、本研究開始後に新たに得られた情報により、研究薬の安全性又は有効性に関して研究継続に重大な問題があることが明らかになった場合、認定臨床研究審査委員会により、研究計画等の変更の指示があり、これを受け入れることが困難と判断された場合、あるいは認定臨床研究審査委員会により、中止の勧告あるいは指示があった場合、研究の中止、又は中断の決定を行う。

研究責任医師が研究の中止の決定を行った場合は、その理由及び以後の対応を直ちに本研究の関係者に連絡し、中止後の処理にあたる。

研究責任医師又は研究分担医師は、研究が中止又は中断された場合、理由の如何を問わず、本研究に参加した研究対象者に速やかにその旨を通知し、適切な措置を講じるとともに、研究対象者の安全を確保するための検査等を実施する。また、研究責任医師は、研究を中止したときは、「中止通知書（統一書式 11）」を実施医療機関の管理者及び認定臨床研究審査委員会に提出する。

13. 目標症例数及び研究期間

13.1. 目標症例数

本研究では、50 例（研究薬投与群：25 例、経過観察群：25 例）を組み入れる。

13.2. 研究実施期間

2022 年 4 月（認定臨床研究審査委員会承認後）～2025 年 3 月（登録締切　2024 年 3 月）

14. 統計学的考察

14.1. 解析対象集団

14.1.1. 最大の解析対象集団（Full Analysis Set：FAS）

本研究に登録され、治療期が開始された症例を「最大の解析対象集団（FAS)」とする。ただし、登録後に重大な研究実施計画書違反があると判明した症例、登録後に不適格であると判明した症例については除外する。

14.1.2. 安全性解析対象集団

本研究に登録され、治療期を開始した症例のうち、安全性に関して解析すべきデータが存在しない症例、割り付けられた研究薬が未投与であることが明らかな症例を除いた集団を「安全性解析対象集団」とする。

14.2. 解析方法

14.2.1. 研究対象者背景及びベースライン値の解析方法

研究対象者背景及びベースライン値について、頻度、割合又は中央値など適切な要約統計量を用いて記述する。

14.2.2. 主要評価項目の解析方法

月経前の Menstrual Distress Questionnaire（MDQ）総スコアのベースライン時から最終評価時までの各時点の変化量を繰り返し測定アウトカムとした線形混合効果モデルによって解析を行う。MMRM（Mixed effect Models for Repeated Measurements）によるモデル化を行い、時点間のアウトカムの相関構造は、無構造（unstructured）とする。固定効果として、比較群（研究薬投与群／経過観察群)、評価時点、比較群と評価時点の交互作用、ベースラインの MDQ 総スコアをモデル化する。無構造モデルによって回帰パラメータが推定不可能な場合には、Toeplitz、Heterogeneous Compound Symmetry、Autoregressive(1)、Compound Symmetry、Variance Components の順に構造を変更する。主要な評価は、MMRM によって推定される、最終時点における MDQ 総スコアの変化値の群間の平均値の差によ

って行う。いずれのモデルのもとでも回帰パラメータが推定不可能な場合には、対応のある 2 標本 t 検定によって、これを評価する。有意水準は両側 5%とする。

副次的に、各周期における、研究薬投与群と経過観察群の MDQ 総スコアの変化値の平均値を最小二乗平均によって推定する。また、各周期における研究薬投与群と経過観察群の MDQ 総スコアの変化値の平均値の差の評価を行う。

14.2.3. 副次評価項目の解析方法

MDQ（月経前総スコア、月経中総スコア、月経後総スコア、各下位尺度スコア）についても、14.2.2 節と同様の MMRM によって分析を行う。いずれのモデルのもとでも回帰パラメータが推定不可能な場合には、対応のある 2 標本 t 検定によって、これを評価する。有意水準は両側 5%とする。各周期における、研究薬投与群と経過観察群ごとのアウトカムの平均値を最小二乗平均によって推定する。また、各周期における研究薬投与群と経過観察群のアウトカムの平均値の差の評価を行う。

睡眠評価、漢方医学的評価、月経関連症状評価、労働生産性評価、研究参加後の全体印象については、研究薬投与群と経過観察群の群ごと、観察周期ごとに基本統計量を集計する。また、各時点の評価値又はベースラインから各時点の変化量に関して、研究薬投与群と経過観察群の群間の比較を行う。Fitbit（スマートウォッチ）による睡眠評価・心拍評価は、研究薬投与群と経過観察群の群ごと、観察周期ごとに要約統計量をまとめ、群間比較をする。有害事象については、発生頻度及び発生割合を事象別、重症度別、重篤度別に算出する。

14.2.4. サブグループによる解析方法

患者背景や加味逍遙散の使用目標による効果の違いを探索的に検討するため、主要評価項目と副次評価項目について、以下の基準にてサブグループを設定し解析を実施する。

1） 患者背景（勤務体系）：夜勤の有無
2） 患者特性（10.1.1 参照）：各項目の有無

14.3. 症例数の設定根拠

同疾患領域の臨床的有効性を示すことが予想される見積もりから [17, 20]、主要評価項目の群間の平均値の差を 6.0、標準偏差を 6.73 とし、有意水準両側 5%で、検出力 80%を達成するサンプルサイズを求めたところ、1 群あたり 21 例の症例が必要となった。中止例等を考慮して、本研究における目標症例数は、1 群あたり 25 例（全体 50 例）とした。

15. 原資料等の直接閲覧

15.1. 原資料の範囲

原資料とは、症例報告書などの元となる文書、データ及び記録をいう。

本研究においては、同意文書、診療録、看護記録、検査データ、研究薬の投与記録、調査票等、研究の事実経過の再現と評価に必要な記録をさす。

15.2. 症例報告書中の原データとすべき項目の特定

以下の項目については、症例報告書に直接記載されたものを原データとして取り扱う。

1） ePRO、スマートフォンアプリ（YaDoc）、Fitbit（スマートウォッチ）で収集したデータ
2） 有害事象の研究薬との因果関係及びコメント
3） 中止理由及びコメント
4） 研究責任医師又は分担医師のコメント

15.3. 原資料等の直接閲覧の保証

研究責任医師は、モニタリング又は監査が実施される場合あるいは認定臨床研究審査委員による調査の際に、原資料等すべての研究関連記録を直接閲覧に供し、これに協力する。

16. 品質管理・品質保証

研究責任医師は、研究に関連するすべてのデータの信頼性とその適正な処理を保証するために、データ取扱いの各段階において品質管理を適用する。なお、本研究では監査は実施しない。

研究責任医師は、本研究について関連法規又は研究実施計画書に適合していない状態（不適合）であると知った時は、速やかに実施医療機関の管理者に報告する。また、不適合であって特に重大なものが判明した場合においては、速やかに認定臨床研究審査委員会の意見を聴き、必要な対応を行う。

16.1. モニタリング

モニタリングとは、臨床研究に対する信頼性の確保及び臨床研究の対象者の保護の観点から臨床研究が適正に行われていることを確保するため、当該臨床研究の進捗状況並びに当該臨床研究が関連法規及び研究実施計画書に従って行われているかどうかについて、研究責任医師が特定の者を指定して行わせる調査をいう。

16.2. モニタリングの方法

本研究では、中央モニタリングで行う。なお、認定臨床研究審査委員会への報告対象となる疾病等の発生あるいは法令、研究実施計画書からの重大な不適合が発生した場合は、必要に応じて、On-site モニタリング及び Off-site モニタリングを組み合わせて行う。なお、モニタリング担当者は、研究責任医師にモニタリング実施後 2 週間以内にモニタリング報告書

を提出する。

16.2.1. モニタリングの準備

モニタリング担当者は、モニタリングの実施に先立ち、下記の確認等を行う。

1) モニタリング実施手続きの確認

　モニタリング担当者は、モニタリング計画に従って研究責任医師等と、当該研究のモニタリング業務に関する協議を行い、モニタリング実施の手続きについて確認する。

2) 研究手順の確認

　症例登録から終了までの手順、研究関係者の役割を確認する。

3) モニタリングの対象とする項目に関する協議

　研究責任医師等とモニタリングの対象とする項目についての協議を行う。

4) モニタリングに必要な資料の入手

　モニタリング担当者は、モニタリングを実施する上で必要な資料を研究責任医師等より入手する。

16.2.2. 研究開始時のモニタリング

モニタリング担当者は、1 例目の登録時点で下記 1）～4）のモニタリングを実施する。

1) 認定臨床研究審査委員会で当該特定臨床研究の実施が承認されていることの確認
2) 実施医療機関の管理者より当該特定臨床研究の実施が承認されていることの確認
3) 登録された研究対象者の同意取得日が登録日の同日又は前の日付であることの確認
4) 登録された研究対象者が研究実施計画書に記載されている選択基準に合致し、除外基準に抵触していないことの確認

16.2.3. 研究実施中のモニタリング

モニタリング担当者は、研究実施中に最初の研究対象者が組み入れられてから原則、半年おきにモニタリングを行う。モニタリングは、全例を対象予定とする。なお、認定臨床研究審査委員会への報告対象となる疾病等及び重大な不適合発生時には、随時モニタリングを実施する。

1) 登録進捗状況及び集積ペースの確認
2) 症例報告書の提出状況の確認
3) 症例報告書の記載漏れ（空欄）及び、報告内容の確認
4) 登録された研究対象者の参加状況の確認
5) 有害事象の発生状況の確認
6) 逸脱及び不適合の確認
7) スマートフォンアプリ（YaDoc）、Fitbit（スマートウォッチ）による評価状況

16.2.4. 研究終了時のモニタリング

モニタリング担当者は、研究終了時に下記のモニタリングを実施する。

1） 問い合わせ事項に未回答がないことの確認

2） すべてのデータが固定されたことの確認

17. 倫理的事項

17.1. 遵守すべき諸規則

本研究に関係するすべての研究者は、ヘルシンキ宣言（世界医師会 1964 年ヘルシンキ総会採択、その後の改正を含む）及び「臨床研究法」（平成 29 年法律第 16 号）及び臨床研究法施行規則（平成 30 年厚生労働省令第 17 号）及びそれらに関連する法令等に従って本研究を実施する。

17.2. 実施医療機関の管理者への定期報告

研究責任医師は、年 1 回以上、本研究の進捗、有害事象、疾病等の発生状況、規則、研究実施計画書に対する不適合の発生状況、安全性・科学的妥当性、医薬品等製造販売業者等の関与に関する事項及び研究実施に際しての問題点等を、実施医療機関の管理者及び認定臨床研究審査委員会に提出し、認定臨床研究審査委員会による研究継続の適否についての審査を受ける。

17.3. 個人情報とプライバシーの保護

研究に関わる関係者は、研究対象者の個人情報保護について、適用される法令、条例を遵守する。また、関係者は、研究対象者の個人情報及びプライバシー保護に最大限の努力を払い、本研究を行う上で知り得た個人情報を正当な理由なく漏らしてはならない。関係者がその職を退いた後も同様とする。研究責任医師又は研究分担医師は、その記述単体で特定の研究対象者を直ちに判別できる記述等（氏名、イニシャル、住所、電話番号、カルテ番号等）を全部取り除き、匿名化を行う。症例登録及び症例報告書等の作成の際には、研究対象者識別コード又は登録番号を用いる。なお、匿名化する際の対応表は、研究責任医師が「21.1.　情報の保管／管理」に従い保管・管理する。

YaDoc 及び Microsoft Forms は、3 省 2 ガイドライン（医療情報を取り扱う事業者が準拠すべき医療情報の保護に関するガイドライン。厚生労働省、経済産業省、総務省の 3 省が発行する 2 つのガイドラインから成る）に準拠しており、アプリを通じて取得した情報のセキュリティが担保されている。また、YaDoc は TLS 1.2 による通信の暗号化を実装している。本研究の YaDoc の開発元である株式会社インテグリティ・ヘルスケアは、外部ベンダーとして、適切なデータマネジメントの下で、これらの各データベースに格納される情報の統合および提供を行う。なお、各データベースには、個人が特定されない形で処理された情報の

みが登録されており、データ統合やデータ提供の作業プロセス、および最終的な提供データには、研究対象者個人を特定できる情報は含まれない。
研究責任医師等が研究で得られた情報を公表する際には、研究対象者が特定できないよう十分に配慮する。

17.4. 臨床研究に参加することによる利益

本研究に参加することにより、研究薬投与により、月経に伴う月経前症状による苦痛を和らげることができる可能性がある。また、PMS に対する加味逍遥散の有効性及び安全性を確認することは、将来の治療薬の選択肢を広め、多くの性成熟期女性の QOL 向上並びに今後の PMS の有効な治療法確立につながる。経過観察群は、直接的な治療効果の利益はないが、研究薬投与群と同様、カウンセリング・生活指導など非薬物療法は受けることができ、また、詳細な診察を行うため、研究対象者の病態の詳細な把握と今後の治療に反映することができる。

17.5. 臨床研究に参加することによる安全性及び不利益への配慮

本研究の研究薬投与群で投与する加味逍遥散は薬事承認薬であり、かつ本研究では承認の範囲内の用法用量で投与を行うが、対象疾患に対しての使用経験は少ない。本研究の実施に伴い、「1.3.　研究計画の根拠」のとおり、副作用等の発現の可能性がある。
本研究では、加味逍遥散投与に伴う有害事象発生のリスクを最小化するために「患者適格基準」、「併用禁止薬・療法」、「検査・観察」、「モニタリング」などを設定している。研究開始後は定期モニタリングが義務付けられており、有害事象が予期された範囲内かどうかをモニターするとともに、重篤な有害事象や予期しない有害事象が生じた場合には慎重に検討・審査され、必要な対策が講じられる体制がとられている。また、採血、各種評価、観察があるが、研究対象者の状態を観察しながら実施する。
経過観察群は、研究薬投与による健康被害の発生リスクはない。しかしながら、同意取得前 4 週間以内に経口避妊薬や性ホルモン製剤、抗うつ薬、抗不安薬、睡眠薬及び漢方薬の治療歴のある患者は、評価への影響を考慮して除外しているが、研究期間中に PMS に対する治療が制限される不利益がある。なお、本研究参加にあたっては、本研究に参加しない場合の他の治療法を説明し、研究対象者の自由意思で研究に参加できるよう配慮する。

17.6. 研究実施後の対応

研究対象者においては、研究終了時の状態を観察し、最善の治療を提供する。

18. 研究の費用負担

18.1. 資金源及び財政上の関係

本研究は、株式会社ツムラから資金提供、研究薬及び評価に使用するアンケートシステ

ム、Fitbit（スマートウォッチ）の提供を受けて実施する。株式会社ツムラは、研究薬に関する情報を提供するが、研究の実施、解析に係わることはない。本研究の実施及び成果に関して利益相反が生じる可能性は、利益相反マネジメントポリシーに基づき、適切にこれを管理するとともに、研究開始前までに鳥取大学医学部附属病院臨床研究審査委員会で審議を行い、適切にこれを管理する。

なお、株式会社ツムラには、本研究の成果報告書、安全性情報、及び研究実施に際しての問題点等を報告する。

18.2. 研究に係る費用と研究対象者負担

本研究は、通常診療の範囲内で行うため、本研究期間中に研究対象者に対して行われる治療に関する医療費には、研究対象者が加入する健康保険を適用し、その自己負担分は研究対象者の負担とする。ただし、臨床検査については、研究費で賄う。また、研究薬は、株式会社ツムラから提供を受ける。スマートフォンを用いたデータ通信費は、研究対象者が負担をする。研究対象者の負担軽減費として、初回は 6,000 円、以降 1 回の規定来院につき 2,000 円を支払う。また、Fitbit（スマートウォッチ）は、研究終了後に研究対象者に提供する（自己都合で中止した場合を除く）。

18.3. 健康被害に対する補償

研究責任医師は、本研究の実施に起因して健康被害が発生し、研究対象者に健康被害が生じた時、研究対象者がただちに適切な診断、治療及び必要な措置を受けることができるように対応する。

研究責任医師は、本研究に関連して研究対象者に生じた健康被害に対する補償措置として、保険に加入（死亡、後遺障害に対する補償金、医療費、医療手当）する。なお、医療費、医療手当については、研究薬投与に起因する入院治療を必要とする程度の未知／既知の副作用のみ補償を行う。

19. 研究実施計画書の承認、改訂、逸脱

19.1. 研究実施計画書等の承認及び改訂

研究責任医師は、研究開始前に研究実施計画書を認定臨床研究審査委員会へ提出し、研究の実施に関して認定臨床研究審査委員会及び実施医療機関の管理者の承認を得る。

研究実施計画書の改訂を行う場合は、研究責任医師は、改訂・変更の決定を研究関係者に速やかに伝達する。研究責任医師は、認定臨床研究審査委員会に改訂版を提出し承認を得なければならない。重大な変更の場合は、研究対象者保護の観点から症例登録の一時中止を検討する。

19.2. 研究実施計画書からの逸脱

研究実施計画書及び法令等からの逸脱について、以下のとおり規定する。

1） 研究責任医師又は研究分担医師は、認定臨床研究審査委員会及び実施医療機関の管理者の承認を得る前に、研究実施計画書からの逸脱あるいは変更を行ってはならない。
2） 研究責任医師又は研究分担医師は、緊急回避等のやむを得ない理由により、認定臨床研究審査委員会の事前の承認を得る前に、研究実施計画書からの逸脱あるいは変更を行うことができる。その際には、研究責任医師又は研究分担医師は、逸脱又は変更の内容、理由及び研究実施計画書等の改訂が必要であればその案を速やかに認定臨床研究審査委員会に提出し、認定臨床研究審査委員会及び実施医療機関の管理者の承認を得る。
3） 研究責任医師又は研究分担医師は、研究実施計画書からの逸脱があった場合は、逸脱事項とその理由を全て記録しなければならない。
4） 研究責任医師は、当該臨床研究について、法令等に適合していないことを知った場合には、速やかに実施医療機関の管理者に報告し、必要な対応をとる。また、不適合の程度が重大である場合は、研究責任医師は、速やかに認定臨床研究審査委員会の意見を聴き、必要な対応を行うとともに、認定臨床研究審査委員会の意見に従って再発防止策を講じる等、必要な措置をとり、臨床研究に従事する者に周知し、当該措置の徹底を図る。

20. 症例報告書

本研究では、EDC（Electronic Data Capture）システム及び ePRO（患者報告アウトカム電子システム）を用いて、データの収集並びに症例報告書の作成及び提出を行う。

20.1. EDC（Electronic Data Capture）システム

20.1.1. EDC システムの運用

本研究では、EDC システム（TUH-RSS）を採用し、症例報告書の作成及び提出を電子的に行う。EDC システムの使用に先立ち、認定臨床研究審査委員会承認後、研究責任医師は実施医療機関の管理者からの承認結果通知書（写）を研究事務局に送付した後、データセンターから研究責任医師、研究分担医師及び研究協力者個人の ID とパスワードを入手し、症例報告書の作成を行う。

本研究では、症例報告書の作成、提出、修正及び閲覧はすべて EDC システムを介して行う。これらの作業に際して、ID 及びパスワードで個人認証が行われ、各作業内容と作成者が電子的に記録される。

20.1.2. 症例報告書の作成

本研究での症例報告書データは、EDCデータベースに電子データとして保管される。

1） 研究責任医師又は研究分担医師は、研究実施計画書に規定されている収集データ項目をEDC入力画面で入力する。
2） 研究責任医師又は研究分担医師は、対象となるすべての研究対象者の症例報告書を作成する。
3） データマネジメント担当者は、入力データのデータレビューを行い、研究責任医師又は研究分担医師への問い合わせが必要な場合には、EDC 上にて問い合わせる。研究責任医師又は研究分担医師は、問い合わせ内容を確認し、必要に応じて入力データの修正を行う。

20.2. ePRO（患者報告アウトカム電子システム）及びアンケート調査

20.2.1. ePRO及びアンケート調査の運用

本研究では、ePRO、スマートフォンアプリ（YaDoc）、Fitbit（スマートウォッチ）及び紙媒体でのアンケート調査を利用してデータを収集し、症例報告書の作成及び提出する。これらのシステムは、株式会社インテグリティ・ヘルスケアのシステムを利用する。ePROについては、株式会社インテグリティ・ヘルスケアで要件を設定後、提供される。スマートフォンアプリ（YaDoc）については、研究対象者のスマートフォンに株式会社インテグリティ・ヘルスケアが提供するアプリをダウンロードして利用する。また、Fitbit（スマートウォッチ）については、株式会社ツムラから提供され、研究対象者のスマートフォンと Bluetooth 接続を行い、データを収集する。

20.2.2. 症例報告書の作成

1） 研究対象者が、研究実施計画書に規定されている収集データ項目をePRO、スマートフォンアプリ（YaDoc）に直接入力する。Fitbit（スマートウォッチ）については、毎日就寝時に装着する。紙媒体アンケート調査は、研究対象者が自己記入する。
2） ePRO、紙媒体アンケート調査で収集されたデータは、Microsoft Formsで構築されるシステムを介して、マイクロソフトのデータベースサーバーに格納される。スマートフォンアプリ（YaDoc）で取得されるデータは、AWS（アマゾンウェブサービス）上に構築されているYaDocデータベースサーバーに格納される。Fitbit（スマートウォッチ）で収集したデータは、Bluetooth接続したスマートフォンを介して、Fitbitデータベースに格納される。
3） データマネジメント担当者は、適宜データレビューを行い、研究責任医師又は研究分担医師への問い合わせが必要な場合には、問い合わせる。研究責任医師又は研究分担医師は、問い合わせ内容を確認する。
4） 株式会社インテグリティ・ヘルスケアのデータベースに格納されたデータは、外部デ

ータとして研究責任医師が研究終了時に受領する。

20.3. 症例報告書データの固定と保管

最終的なデータ入力・修正が完了後、症例報告書データ（修正記録等を含む）をデータセットとして固定する。データセットは、研究責任医師の責任の下、本研究で規定する保管期間まで適切に保管する。

21. 情報の保管／管理

21.1. 情報の保管／管理

研究責任医師は、研究等の実施に係わる文書（申請書類の控え、実施医療機関の管理者からの通知文書、各種申請書・報告書の控、同意文書、症例報告書、対応表、その他データの信頼性を担保するのに必要な書類又は記録等）を鳥取大学医学部附属病院女性診療科医局の鍵のかかるロッカーに保管する。また、電子データについては、本研究に関与する関係者のみアクセスできるパソコンを用い、パスワードで管理する。保管期間は、当該研究の終了日から 5 年を経過した日までの期間とする。保管期間終了後は、紙媒体に関してはシュレッダーで裁断し廃棄する。その他媒体に関しては、匿名化のうえ適切な方法で廃棄する。

21.2. 情報の二次利用

本臨床研究のために集められた情報を、現時点では、計画・予測されていないものの、将来非常に重要な検討が必要となる場合に本研究とは別の目的の研究で利用することがある。こうした情報の二次利用に関しては、倫理審査委員会又は認定臨床研究審査委員会で審査・承認された後、可能な限り研究対象者に改めて説明の上、同意を取得するが、説明できない場合には、該当する指針に基づき研究の情報の公開等を行う。この際も、情報に個人を特定できる情報を含むことはない。

22. 研究の概要及び結果の登録

本臨床研究は、研究実施に先立ち事前に厚生労働省が整備するデータベース（Japan Registry of Clinical Trial：jRCT）に当該臨床研究に関する情報を登録し、公表する。データベースに登録した情報は、研究実施計画書の変更及び研究の進捗に応じて適宜更新し、また、研究を終了したときは、遅滞なく、当該研究の結果を登録する。

23. 研究成果の帰属と研究結果の公表

本研究の実施に伴い特許権が発生する可能性は低いが、本研究で得られた成果による知的財産権が発生した場合は、鳥取大学及び株式会社ツムラに帰属する。研究の結果は、研究

を終了した 2024 年以降に学会における発表及び学術論文として公表する。なお、研究責任医師以外の研究者が、研究で得られた成果を論文又は学会等にて発表する場合は、研究責任医師が協議のうえ取り決める。

24. 研究組織

24.1. 研究責任医師

研究責任医師は、本研究に係る各実施医療機関の業務を統括するとともに、研究分担医師、研究協力者によるチームの責任者として管理・指導する。

谷口　文紀　鳥取大学医学部附属病院　女性診療科　教授
〒683-8504　鳥取県米子市西町 36-1
TEL：0859-38-6647

24.2. 研究事務局

研究代表医師の支援及び事務局業務を行う。

担当者　遠藤　佑輔
鳥取大学医学部附属病院　新規医療研究推進センター　臨床研究支援部門
〒683-8504　鳥取県米子市西町 36-1
TEL：0859-38-6946／FAX：0859-38-6947

24.3. データマネジメント担当者

本研究における症例登録、データ管理、データセンター業務を行う。

担当者　砂田　寛司
鳥取大学医学部附属病院　新規医療研究推進センター　臨床研究支援部門
〒683-8504　鳥取県米子市西町 36-1
TEL：0859-38-6946／FAX：0859-38-6947

24.4. モニタリング担当者

本研究におけるモニタリング業務を行う。

担当者　砂田　寛司
鳥取大学医学部附属病院　新規医療研究推進センター　臨床研究支援部門
〒683-8504　鳥取県米子市西町 36-1
TEL：0859-38-6946／FAX：0859-38-6947

24.5. 統計解析担当者

本研究における統計解析業務を行う。

担当者　野間　久史

大学共同利用機関法人情報・システム研究機構統計数理研究所　データ科学研究系
総合研究大学院大学　複合科学研究科　統計科学専攻
鳥取大学医学部附属病院　教育・研究顧問（生物統計）
〒190-8562　東京都立川市緑町 10-3
TEL：050-5533-8440／FAX：042-526-4339

24.6. 研究・開発計画支援担当者

本研究における研究薬の特徴を踏まえ各段階での意思決定基準を提示する業務、並びに研究実施計画書の基本骨格を作成する業務の支援を行う。

担当者　遠藤　佑輔
鳥取大学医学部附属病院　新規医療研究推進センター　臨床研究支援部門
〒683-8504　鳥取県米子市西町 36-1
TEL：0859-38-6946／FAX：0859-38-6947

24.7. 共同研究者

研究計画の検討、研究費の提供、研究薬にかかわる情報、安全性情報等の提供を行う。なお、株式会社ツムラは、研究の実施、解析に係わることはない。

株式会社ツムラ　研究開発本部
〒107-8521　東京都港区赤坂 2-17-11
TEL：03-6361-7188／FAX：03-5574-6664
責任者：執行役員 研究開発本部長　今田　明人

24.8. 外部委託機関

ePRO（患者報告アウトカム電子システム）の設計、データベース管理を行う。

株式会社インテグリティ・ヘルスケア
〒103-0014　東京都中央区日本橋蛎殻町 1-27-5 YAMATO B.L.D
TEL：03-6661-0858
責任者：代表取締役社長　園田　愛

【委託先の監督方法】

本研究に関する業務を外部委託する場合、委託先は個人情報、秘密情報の保管、取り扱いについて個人情報保護法等に基づき適切な安全管理措置を講じるものとする。また、委託元は当該措置を確認する必要がある場合には監査を行い、十分に措置が講じられていない場合には措置を講じるように勧告を行う。勧告にもかかわらず改善されない場合は、委託元は委託契約の解除も含めた対応策を検討する。

25. 参考文献

1. 日本産科婦人科学会 (編),産科婦人科用語集・用語解説集改訂第 4 版, 2018.
2. American College of Obstetricians and Gynecologists, Premenstrual syndrome. Guideline for Women’s Health Care. A Resource Manual, Fourth Edition,2014,607-613.
3. Steiner M et al. The premenstrual symptoms screening tool (PSST) for clinicians.*Arch Women’s Ment Health* 2003,6:203-209.
4. Takeda T et al. Prevalence of premenstrual syndrome and premenstrual dysphoric disorder in Japanese high school students.*Arch Women’s Ment Health* 2010,13: 535-537.
5. 日本医療政策機構 働く女性の健康増進に関する調査, 2018.
6. RCOG Green-top Guideline BJOG 2016, No48.
7. 日本産科婦人科学会/日本産科婦人科医会 (編・監)産婦人科診療ガイドライン婦人科外来編 2020, 2020, 174-176.
8. 厚生労働省医薬食品局, 一般用漢方製剤承認基準の制定について. 平成 20 年 9 月 30 日, 薬食審査発第 0930001 号.
9. 厚生労働省 試験問題作成に関する手引き（平成 30 年 3 月）.
10. 飯塚進・他 産婦人科漢方研究のあゆみ No.16. 診断と治療社, 1999, p.37.
11. Mizowaki, M et al. Anxiolytic effect of Kami-Shoyo-San (TJ-24) in mice: possible mediation of neurosteroid synthesis. *Life Sci*.2001,69 (18), p.2167-2177.
12. 飯島治・他. 薬理と治療. 1995, 23 (suppl.7), p.1813.
13. 窪庭晴男・他. 薬理と治療. 1999, 27 (suppl.6), p.1413.
14. Yamada, K. et al. Effectiveness of kamishoyosan for premenstrual dysphoric disorder: open-labeled pilot study. *Neurosci.* 2007, 61 (3), p.323.
15. Kiyoshi Takamatsu et al. A Multicenter, Randomized, Double-Blind, Placebo-Controlled Trial to Investigate the Effects of Kamishoyosan, a Traditional Japanese Medicine, on Menopausal Symptoms: The KOSMOS Study. Evid Based Complement Alternat Med.2021 26; 2021: 8856149.
16. ツムラ加味逍遙散エキス顆粒 (医療用) 医薬品インタビューフォーム 2020 年 11 月 (改訂第 9 版).
17. Moos et al. The development of a menstrual dis- tress questionnaire. Psychosom Med 30: 853-. 868.1968.
18. 堀 洋道・他 心理測定尺度集 III,サイエンス社 2011 p.272.
19. 長塚正晃. 11)月経前症候群.日本産科婦人科學會雜誌. 61(12).2009.
20. D. Berger at el. Efficacy of Vitex agnus castus L. extract Ze 440 in patients with pre-menstrual syndrome (PMS). Arch Gynecol Obstet 264:150–153.2000.
21. Hae Won Kim et al. Intake of dietary soy isoflavones in relation to perimenstrual symptoms of

Korean women living in the USA. *Nurs Health Sci* 2006,8 (2) p108-13.

22. Momoeda M. et al. Efficacy and safety of Vitex agnus-castus extract for treatment of premenstrual syndrome in Japanese patients: a prospective, open-label study. *Adv Ther.* 2014; 362-73.
23. Taniguchi F et al. Efficacy of Tokishakuyakusan add-on therapy with low-dose oral contraceptive pills on endometriosis patients with dysmenorrhea. *J Obstet Gynaecol Res.* 2020 Nov;46(11): 2280-2286.
