# Supplementary material for: A Kampo medicine, kamishoyosan, ameliorates menstruation-associated symptoms in patients with premenstrual syndrome: An exploratory open-label, randomized controlled trial
Source: PLoS One. 2026 Nov 6;21(11):e0362303. doi: 10.1371/journal.pone.0362303 (PMC13742958; doi:10.1371/journal.pone.0362303)
Supplement: Supplementary file 2 [file pone.0362303.s004.pdf]

ENGLISH TRANSLATION

Clinical Study Protocol

An Open-Label, Randomized Clinical Study to Evaluate the Efficacy and Safety of Kamishoyosan for Premenstrual Syndrome

Study No.: 21C004

Translation of the Japanese protocol, Version 1.3 dated April 15, 2024

Note: This document is an English translation of the Japanese protocol. The Japanese protocol remains the source document.

Principal Investigator: Fuminori Taniguchi, Professor, Department of Women's Medicine, Tottori University Hospital
Address: 36-1 Nishi-cho, Yonago, Tottori, Japan   TEL: 0859-38-6647 / FAX: 0859-38-6649

Study Secretariat: Yusuke Endo, Advanced Medicine, Innovation and Clinical Research Center, Tottori University Hospital
Address: 36-1 Nishi-cho, Yonago, Tottori, Japan   TEL: 0859-38-6946 / FAX: 0859-38-6947

Emergency contact: 0859-38-6913 (Department of Women's Medicine, Tottori University Hospital)
Planned study period: April 2022 (after approval by the Certified Review Board) to March 2025
Date prepared: April 15, 2024 (Version 1.3)

0. Study Overview

0.1. Summary of the Study Plan

Objective	To evaluate the efficacy and safety of kamishoyosan by comparing a kamishoyosan treatment group with an observation group in patients with premenstrual syndrome (PMS).
Endpoints	Primary endpoint:<br>Change in the total premenstrual Menstrual Distress Questionnaire (MDQ) score from baseline to the final assessment.<br><br>Secondary endpoints:<br>(1) longitudinal changes in MDQ total and subscale scores before, during, and after menstruation<br>(2) sleep assessment<br>(3) Kampo medicine assessment<br>(4) menstruation-associated symptom assessment<br>(5) work productivity assessment; (6) coldness and stiff-shoulder scores<br>(7) overall impression after study participation<br>(8) sleep and heart-rate assessment using Fitbit (smartwatch)<br>(9) adverse events.
Study design	Open-label, randomized clinical study.
Inclusion Criteria	1) Patients diagnosed with PMS based on ACOG PMS diagnostic criteria<br>2) Patients who meet a total score of 30 or higher on premenstrual MDQ at pretreatment period<br>3) Patients aged 18 to 45 at the time of consent<br>4) Patients with regular menstrual cycle of 25 to 38 days<br>5) Patients who can take study drugs orally<br>6) Outpatient
Exclusion Criteria	1) Patients who have been treated with oral contraceptives or sex hormones within 4 weeks before obtaining consent<br>2) Patients who have been treated with antidepressants within 4 weeks before obtaining consent<br>3) Patients who have been treated with anxiolytics within 4 weeks before obtaining consent<br>4) Patients who have been treated with sleeping pills within 4 weeks before obtaining consent<br>5) Patients who have been treated with diuretics within 4 weeks before obtaining consent<br>6) Patients who have been treated with effective drug against PMS within 4 weeks before obtaining consent<br>7) Patients with serious mental disease<br>8) Patients with serious disease<br>9) Patients who are pregnant or may be pregnant and wish to become pregnant<br>10) Patients who are lactating women<br>11) Patients with allergies to Kampo medicines<br>12) Patients who participated in clinical trials or other clinical studies within 3 months before obtaining consent

	13) Patients treated with study drug within 4 weeks prior to consent
Target sample size	50 participants (study-drug group: 25; observation group: 25).
Study drug	TJ-24 TSUMURA Kamishoyosan Extract Granules for Ethical Use.
Study period	April 2022 (after approval by the Certified Review Board) to March 2025.

0.2. Abbreviations and Definitions

Abbreviation	Name
ACOG	American College of Obstetricians and Gynecologists
MDQ	Menstrual Distress Questionnaire
PMS	Premenstrual syndrome
WPAI	Work Productivity and Activity Impairment Questionnaire

Contents

0. Research Overview 1

0.1. Overview of the Research Implementation Plan 1

0.2. Definitions of Abbreviations and Terms 3

1. Background and Rationale 7

1.1. Pathophysiology and Epidemiology of Premenstrual Syndrome 7

1.2. Treatment of Premenstrual Syndrome 7

1.3. Rationale for the Study 7

1.4. Significance of the Study 9

2. Objectives 9

3. Evaluation Criteria 9

3.1. Primary Endpoint 9

3.2. Secondary Endpoints 9

4. Study Participants 9

4.1. Inclusion Criteria 9

4.2. Exclusion Criteria 9

5. Explanation to Participants and Informed Consent 10

5.1. Preparation and Revision of the Participant Information and Consent Forms 10

5.2. Explaining the Study to Research Participants and Obtaining Consent 11

6. Participant Registration and Randomization 11

6.1. Registration Procedures 11

6.2. Randomization 11

7. Study Methods 11

7.1. Study Design 11

7.2. Study Procedures 12

7.3. Dosage and Administration Period 12

7.4. Dosage Modification and Temporary Interruption 13

7.5. Concomitant Medications, Therapies, and Foods 13

7.6. Planned Duration of Participation 13

8. Study Drug Management 13
8.1. Study Drug 13
8.2. Management of Study Drug 13
9. Discontinuation Criteria 14
10. Observation/Test Items and Schedule 15
10.1. Testing/Observation Items 15
10.2. Schedule of Assessments 17
11. Adverse Event Assessment/Reporting 19
11.1. Definitions of Adverse Events and Adverse Drug Reactions 19
11.2. Adverse Events Reporting 19
11.3. Periodic Reporting of Diseases or Other Events 21
11.4. Provision of Information to Marketing Authorization Holders 21
12. Completion, Discontinuation, and Suspension of Study 21
12.1. Study Completion 21
12.2. Study Discontinuation/Suspension 21
13. Target Sample Size and Study Period 21
13.1. Target Sample size 21
13.2. Study Period 21
14. Statistical Considerations 21
14.1. Analysis Populations 21
14.2. Analysis Methods 21
14.3. Rationale for Sample Size 22
15. Direct Access to Source Documents 22
15.1. Scope of Source Documents 22
15.2. Items Treated as Source Data in the Case Report Form 22
15.3. Assurance of Direct Access 23
16. Quality Control / Quality Assurance 23
16.1. Monitoring 23
16.2. Monitoring Methods 23

17. Ethical Considerations 24

17.1. Applicable Rules 24

17.2. Periodic Reports to the Administrator of the Study Institution 24

17.3. Protection of Personal Information and Privacy 24

17.4. Benefits of Participation 25

17.5. Safety and Disadvantages of Participation 25

17.6. Post-study Care 25

18. Study Costs 25

18.1. Funding Sources and Financial Relationships 25

18.2. Study Costs and Participant Expenses 25

18.3. Compensation for Health Injury 25

19. Protocol Approval, Revision, and Deviations 25

19.1. Approval and Revision 25

19.2. Deviations from the Protocol 26

20. Case Report Forms 26

20.1. EDC System 26

20.2. ePRO and Questionnaire Surveys 26

20.3. Case Report Form Data Lock and Storage 27

21. Information Storage and Management 27

21.1. Storage and Management 27

21.2. Secondary Use of Information 27

22. Registration of Study Summary and Results 27

23. Ownership and Publication of Study Results 27

24. Research Organization 27

24.1. Principal Investigator 27

24.2. Study Secretariat 27

24.3. Data Manager 28

24.4. Monitor 28

24.5. Statistical Analyst 28

24.6. Research and Development Planning Support 28

24.7. Collaborating Research Organization 28

24.8. External Contractor 28

25. References 29

1. Background and Rationale

1.1. Pathophysiology and Epidemiology of Premenstrual Syndrome

Premenstrual syndrome (PMS) is defined as mental or physical symptoms that persist during the luteal phase, 3–10 days before menstruation, and decrease or disappear with the onset of menstruation.[1)] Mental symptoms include depression, outbursts of anger, irritability, and related symptoms; physical symptoms include breast tenderness or swelling, abdominal bloating, headache, and related symptoms.[2)] PMS occurs across a wide age range among women of reproductive age[3)], although a somewhat higher prevalence among adolescent women has been reported in Japan.[4)] A 2018 internet-based survey conducted in Japan reported that 66% of 2,000 full-time working women had current or previous PMS symptoms and that 45% reported their performance was reduced to half or less of their usual level because of PMS or menstruation-associated symptoms.[5)] The etiology of PMS remains unclear; associations with sensitivity to progesterone and involvement of the neurotransmitters serotonin and gamma-aminobutyric acid (GABA) have been reported.[6)]

1.2. Treatment of Premenstrual Syndrome

Japanese obstetrics and gynecology clinical guidelines list non-pharmacological approaches such as counseling, lifestyle guidance, and exercise as first-line management of PMS, and recommend cognitive-behavioral approaches that promote recognition and understanding of the condition through symptom diaries. Pharmacological treatments described include diuretics such as spironolactone for edema and breast tenderness, analgesics, and Kampo medicines. Low-dose estrogen-progestin combinations (OC/LEP) are another option, but are not approved for PMS in Japan[7)]. Selective serotonin reuptake inhibitors (SSRIs) are considered first-line drugs for PMS in Western countries, but are not covered by insurance for PMS in Japan. [7)]

1.3. Rationale for the Study

1.3.1. Overview of the Study Drug

The study drug is kamishoyosan [product name: TSUMURA Kamishoyosan Extract Granules for Ethical Use]. Its approved indications include the following symptoms in women of delicate constitution who are easily fatigued and are apt to have shoulder stiffness, psychoneurotic symptoms including anxiety, and sometimes tendency to constipation: Oversensitivity to cold, delicate constitution, menstrual irregularity, dysmenorrhea, climacteric disturbance and Chi-no-michi-sho* peculiar to women resembling climacteric disturbance. The usual adult dosage is 7.5 g/day orally in two or three divided doses before or between meals.
*Chi-no-michi-sho refers to various symptoms, mainly neuropsychiatric symptoms, associated with physiological phenomena specific to women and accompanying hormonal fluctuations related to menstruation, pregnancy, childbirth, the postpartum period, menopause, and similar states[8)]; PMS is included in this concept.[9)]

1.3.2. Summary of Nonclinical Findings

No nonclinical pharmacological efficacy data specific to PMS are available for kamishoyosan. Effects in menopausal symptom models have been reported, including suppression of stress-induced shortening of pentobarbital sodium-induced sleep time in ovariectomized mice. An anxiolytic effect in normal mice has also been reported, and activation of GABA-A/benzodiazepine receptors has been proposed as a possible mechanism.

Nonclinical safety information:

- Single-dose toxicity

Animal	Route of administration	Sex	Approximate lethal dose (g/kg)
SD rat	Oral	Male	>8
		Female	>8

- Repeated-dose toxicity[12)]: after oral administration to SD rats at 125, 500, or 2,000 mg/kg/day for 3 months, a mild decrease in red blood cell count was observed in both sexes at 2,000 mg/kg/day; this change recovered after a 4-week withdrawal period.[12]
- Reproductive and developmental toxicity: no applicable data.
- Genotoxicity[13)]: no genotoxicity was observed in bacterial reverse-mutation, chromosomal-aberration in cultured mammalian cells, or mouse micronucleus tests.[13]

1.3.3. Summary of Clinical Findings

The efficacy of kamishoyosan for PMS has not yet been demonstrated by any evidence. However, among 26 patients with premenstrual dysphoric disorder (PMDD) whose predominant symptoms were mental symptoms severe enough to interfere with daily life and who wished to receive Kampo medicine, administration of 7.5 g/day for six menstrual cycles resulted in remission in 14 of 26 patients (53.8%).[14)] A double-blind, placebo-controlled, randomized parallel-group study in women aged 40–60 years with menopausal symptoms also reported improvement in an agitation/irritability score after 12 weeks among premenopausal women compared with placebo. For kamishoyosan, adverse-drug-reaction frequencies have not been evaluated in a drug-use-results survey.[15)]

Although no formal surveys on the incidence of adverse reactions—such as post-marketing surveillance—have been conducted for Kamishoyosan, it has been reported that the following serious adverse reactions may occur: pseudoaldosteronism, myopathy, liver dysfunction, jaundice, and mesenteric phlebosclerosis. Other potential adverse reactions include hypersensitivity and gastrointestinal symptoms.[16)]

1.3.4. Study Plan

This study is a parallel-group comparative trial in which women with PMS symptoms will be randomly assigned to either the study drug group or the observation group. Evaluation will involve comparing premenstrual symptoms over three menstrual cycles following administration of the study drug with those in the observation group. The primary inclusion criteria for patients were compliance with the ACOG (American College of Obstetricians and Gynecologists) diagnostic criteria for PMS, a premenstrual Menstrual Distress Questionnaire (MDQ) total score of 30 or higher during the pretreatment period, and a normal menstrual cycle. The validity of the MDQ score was assessed based on previously reported data from PMS patients[17]. The primary endpoint is to compare the change in the premenstrual MDQ total score in the study drug group after three cycles with that of the observation group. The MDQ was developed in 1968 by Moos et al.[17] as a scale to measure both physical and psychological complaints associated with the menstrual cycle; the Japanese version was created by Kayashima et al. in 1979. In Japan, it has been cited as a questionnaire for evaluating PMS symptoms in publications such as the Journal of the Japan Society of Obstetrics and Gynecology[19]) and has been adopted in numerous studies. It has also been used in clinical trials[20]), such as those evaluating the efficacy of “Prefemin,” a premenstrual treatment drug developed by Max Zeller Söhne AG in Switzerland. Given its widespread use in numerous domestic and international publications, it was designated as the primary endpoint in this study. The secondary endpoints were: 1) total MDQ scores and changes over time in the subscales during the premenstrual, menstrual, and postmenstrual periods; 2) work productivity, sensitivity to cold and stiff shoulders, and overall impression after study participation; 3) sleep, Kampo medicine, and menstrual-related symptoms as recorded via the smartphone app (YaDoc); and 4) sleep and heart rate data from Fitbit (smartwatch). Data will be aggregated by group (study drug group vs. observation group) and by observation period, and t-tests will be performed to compare changes between the study drug group and the observation group. Additionally, to exploratively examine differences in efficacy based on patient background and the intended use of Kamishoyosan, subgroups will be defined based on patient characteristics and work schedules, and analyses will be conducted. Safety assessments will include physical examinations (e.g., presence of edema) and measurements of blood pressure and pulse rate at each clinic visit, as well as blood tests at enrollment and at the end of the study. Safety assessment items were established based on existing information[16]). The study schedule defines the period from study subject enrollment until the onset of the next menstrual period as the pre-treatment phase, which is intended to standardize the start of the menstrual cycle. Subsequently, during the treatment phase, participants in the study drug group will begin taking the study drug on the first day of their

menstrual period and continue for up to three cycles. Study subjects will visit the clinic once during the pre-treatment phase and once per cycle after starting the medication for testing and observation. In addition, participants will complete an online questionnaire at home on the first day of menstruation during the second and third cycles, as well as during the fourth cycle of the post-observation period. Furthermore, throughout the study period, participants will complete daily diary entries via a smartphone app (YaDoc) and provide data on sleep and heart rate assessments via a Fitbit (smartwatch). The evaluation period and time points were established based on previously published reports[15, 21, 22, 23].

1.4. Significance of the Study
In Japan, a standard treatment for PMS has not been established. This study evaluates the efficacy and safety of kamishoyosan for PMS using questionnaires, smartphone-based diaries, and Fitbit smartwatches. If an effect of kamishoyosan on PMS is demonstrated, the findings may broaden future treatment options and contribute to improved quality of life for women of reproductive age.

2. Objective
To evaluate the efficacy and safety of kamishoyosan by comparing a kamishoyosan treatment group with an observation group in patients with PMS.

3. Endpoints
3.1. Primary Endpoint
Change in the total premenstrual MDQ score from baseline to the final assessment.

3.2. Secondary Endpoints
1. Longitudinal changes in MDQ total and subscale scores before, during, and after menstruation.
2. Sleep assessment.
3. Kampo medicine assessment.
4. Assessment of menstruation-associated symptoms.
5. Work productivity assessment.
6. Coldness and stiff-shoulder scores.
7. Overall impression after study participation.
8. Sleep and heart-rate assessment using Fitbit (smartwatch).
9. Adverse events.

4. Study Participants
Individuals who meet all inclusion criteria and none of the exclusion criteria will be enrolled.

4.1. Inclusion Criteria
1. Patients diagnosed with PMS according to ACOG diagnostic criteria.
2. Patients with a total premenstrual MDQ score of at least 30 during the pretreatment period.
3. Patients aged 18 to 45 years at the time informed consent is obtained.
4. Patients with regular menstrual cycles of 25 to 38 days.
5. Patients able to take the study drug orally.
6. Visit type: outpatient.
7. Patients who provide written informed consent to participate in the study.

4.2. Exclusion Criteria
1. Treatment with oral contraceptives or sex-hormone preparations within 4 weeks before informed consent.
2. Treatment with antidepressants within 4 weeks before informed consent.
3. Treatment with anxiolytics within 4 weeks before informed consent.
4. Treatment with hypnotics within 4 weeks before informed consent.

5. Treatment with diuretics within 4 weeks before informed consent.
6. Treatment within 4 weeks before informed consent with drugs, OTC products such as Prefemin, or supplements reported to be effective for PMS.
7. Serious psychiatric illness.
8. Serious comorbidity, including gynecological, hepatic, renal, cardiac, hematological, metabolic disease, or malignancy.
9. Pregnant, possibly pregnant, or wishing to become pregnant.
10. Breastfeeding.
11. Allergy to Kampo medicine.
12. Participation in a clinical trial or another clinical study within 3 months before informed consent.
13. Treatment with Kampo medicine, including the study drug, within 4 weeks before informed consent.
14. Any other condition judged by a physician to make the individual unsuitable for participation.

5. Explanation to Participants and Informed Consent

5.1. Preparation and Revision of the Participant Information and Consent Forms

The Principal Investigator will prepare the informed consent form and other documents used to obtain consent from research participants to participate in this clinical study. Furthermore, if, during the course of this study, new and important information becomes available that could influence a research participant's consent, or information that could influence a research participant's decision regarding continued participation in this study, the Principal Investigator will revise these documents accordingly. Any prepared or revised informed consent forms and other documents must be reviewed in advance by a Certified Review Board before use. The informed consent document shall include the following items:

1) The title of the clinical research to be conducted; a statement indicating that approval for conducting said clinical research has been obtained from the administrator of the participating medical institution; and a statement indicating that the research plan has been submitted to the Minister of Health, Labor and Welfare
2) The name of the participating medical institution, as well as the name and title of the Principal Investigator (in the case of a specified clinical study conducted as a multicenter collaborative study, this shall include the name and title of the lead investigator, as well as the names of the other participating medical institutions and the names and titles of the Principal Investigators at those institutions).
3) The reasons for selecting the participant as a subject of the clinical study
4) The expected benefits and risks of participating in the clinical study
5) A statement that refusal to participate in the clinical research is voluntary
6) Matters concerning the withdrawal of consent
7) A statement that the subject will not be subject to any disadvantageous treatment for refusing to participate in the clinical research or for withdrawing consent
8) Methods for disclosing information regarding the clinical research
9) A statement that, upon request by the subject or their authorized representative, the research protocol and other materials related to the conduct of the clinical research may be obtained or inspected, along with the methods for obtaining or inspecting them
10) Regarding the protection of participants' personal information
11) Methods for the storage and disposal of specimens, etc.
12) Status regarding the involvement of manufacturers and distributors of pharmaceuticals, etc.
13) System for handling complaints and inquiries
14) Information regarding costs associated with conducting the clinical research
15) The availability and details of other treatment options, as well as a comparison with the anticipated benefits and risks of those other treatment options
16) Matters concerning compensation for health damage resulting from the conduct of the clinical research and the provision of medical care
17) Matters subject to review by the Certified Review Board responsible for reviewing the clinical research, as well as other matters concerning the Certified Review Board related to the clinical research in question
18) Other matters necessary for the conduct of the clinical research

5.2. Explanation to Participants and Obtaining Consent

Prior to the start of the study, the Principal Investigator or Sub-Investigator will provide a thorough explanation to the prospective research subject using the informed consent document and consent form approved by the Certified Review Board, and will obtain the research subject’s free and informed consent to participate in the study in writing. When obtaining consent, the Principal Investigator or Sub Investigator will allow the research subject sufficient time to decide whether to participate in the study, provide an opportunity to ask questions, and answer those questions thoroughly. The Principal Investigator or Sub-Investigator who provided the explanation, as well as the research subject, shall sign the consent form and each shall enter the date. The consent form shall be kept at the participating medical institution, and copies of the informed consent document and the consent form shall be provided to the research subject.

If, during the course of this study, new important information that could influence the research subject’s consent, or information that could influence the research subject’s decision regarding continued participation in this study, the Principal Investigator shall promptly revise the informed consent document and other materials based on such information. After obtaining approval from the Certified Review Board (CRB), the Principal Investigator or Sub-Investigator will promptly explain the relevant information to the research subject, obtain written consent for continued participation in this study based on the subject’s free will, and provide the research subject with a copy of the consent form. Furthermore, participants may withdraw their consent at any time after giving it and will not suffer any disadvantage as a result of such withdrawal.

6. Participant Registration and Randomization

6.1. Registration Procedure

1. Obtain written informed consent and confirm that all inclusion criteria are met and no exclusion criterion applies.
2. Record the date of informed consent and information necessary to link the participant to the participant identification code in the correspondence table retained by the Principal Investigator, while removing descriptions that by themselves would immediately identify a specific participant.
3. Register the participant via the web-based research support cloud service APS-Cloud (TUH-RSS). Under the direction and supervision of the Principal Investigator, study collaborators may enter and transmit information.
4. If the web-based eligibility assessment confirms eligibility, a registration number is issued.

- The study must not begin for an individual until registration is complete.
- Once registered, a participant cannot be deleted from the database; in the event of duplicate registration, the first registration is retained.
- Erroneous or duplicate registrations must be reported promptly to the data center.

Data Center: Advanced Medicine, Innovation and Clinical Research Center, Tottori University Hospital,
Contact: Hiroshi Sunada
Address: 36-1 Nishi-cho, Yonago, Tottori, Japan.
TEL: 0859-38-6946 / FAX: 0859-36-6947.

6.2. Randomization

Participants will be randomly assigned in a 1:1 ratio to the study-drug treatment group or observation group, using a pretreatment MDQ score of 80 or higher as a stratification factor. Block randomization will be used.

7. Study Methods

7.1. Study Design

This is an open-label, randomized clinical study with a study-drug treatment group receiving kamishoyosan and an observation group receiving no study drug.

7.2. Study Procedures

This study consists of a pre-treatment period, a treatment period, and a follow up period. Starting from the menstrual cycle in which administration of the study drug begins (Cycle 1), informed consent is obtained during Cycle –1 (pre-treatment period). After confirming that participants meet the inclusion criteria and do not violate the exclusion criteria, they are enrolled and randomized (study drug group : observation group = 1:1).During Cycle –1 (pre-treatment period), participants must visit the hospital on the first day of menstruation for that menstrual cycle (within +14 days). Subsequently, the study transitions to the treatment phase starting on the first day of menstruation in Cycle 1, at which point the study drug group begins receiving the study drug and the observation group begins observation. The treatment phase shall last through Cycle 3. For each menstrual cycle, three periods are defined: menstrual, postmenstrual, and premenstrual. Specifically, the menstrual period is defined as the duration of menstrual bleeding during the cycle; the premenstrual period is defined as the days from the first day of the menstrual cycle as day 1—days –7 to –1; and “postmenstrual”: the period excluding the premenstrual and menstrual phases. Participants will visit the hospital on the first day of menstruation (or within 14 days thereof) during the second and third cycles of the treatment phase and the fourth cycle of the post-observation phase to undergo testing and observation. In addition, an online questionnaire survey is conducted at home on the first day of menstruation during the second and third cycles of the treatment phase and the fourth cycle of the post-observation phase. Furthermore, throughout the study participation period, participants use a smartphone app (YaDoc) daily (once a day, at the same time whenever possible) to assess sleep, evaluate their condition according to traditional Chinese medicine principles, and evaluate menstrual-related symptoms.At bedtime during the treatment period, participants will wear a Fitbit (smartwatch) daily to monitor sleep and heart rate. If a participant withdraws from the study during the treatment period, they must visit the hospital to undergo a withdrawal examination.

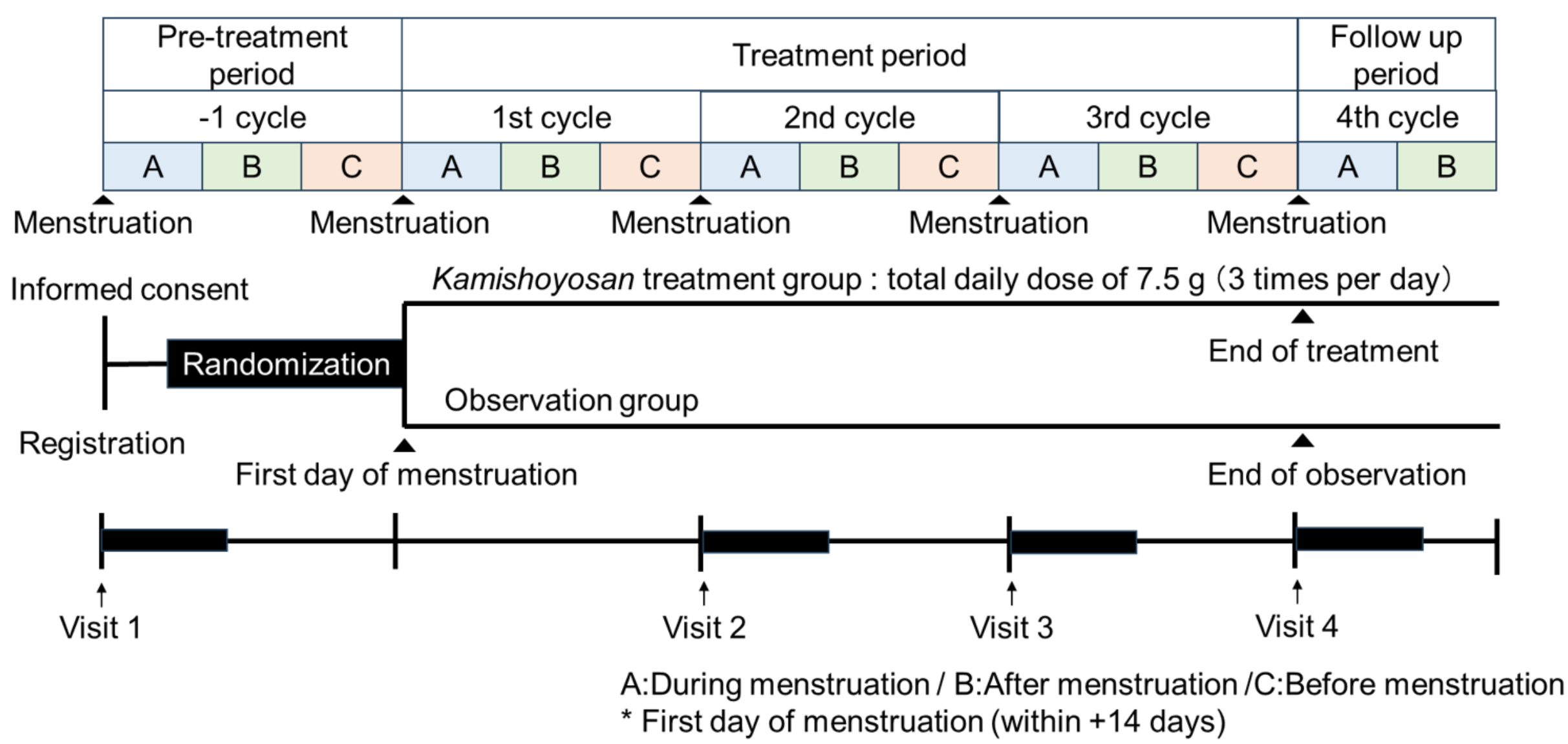

Figure 7-1

7.3. Dosage and Administration Period

The study drug is administered orally at 7.5 g/day in three divided doses of 2.5 g before or between meals. If a dose is missed before or between meals, administration after meals is permitted. Treatment continues from cycle 1 through cycle 3.

7.4. Dose Modification and Temporary Interruption

Changes to the dosage or administration regimen of the study drug are prohibited.

7.5. Concomitant Medications, Therapies, and Foods

7.5.1. Prohibited Concomitant Medications

1. Oral contraceptives and sex-hormone preparations.
2. Hypnotics, anxiolytics, and antidepressants.
3. Diuretics.
4. Drugs reported to be effective for PMS, including OTC Prefemin and supplements.
5. Investigational drugs not approved in Japan, other study drugs, or investigational therapies.
6. Kampo medicines other than the study drug.

7.5.2. Restricted Concomitant Medications

Medications, including supplements, and therapies, including exercise therapy and yoga, that had been used continuously from at least 3 months before informed consent may be continued, but their dosage, method of use, or implementation must not be changed from informed consent through the end of the treatment period. Analgesics, including as-needed use, are permitted under the same condition. Any unavoidable change must be recorded in the case report form. Drugs identified in the package insert as requiring caution in concomitant use, including products containing Glycyrrhiza or glycyrrhizic acid or its salts, may be used concomitantly; however, caution is required during study-drug treatment because of possible interactions with kamishoyosan.

7.6. Planned Duration of Participation

Each participant's planned participation comprises cycle -1 as the pretreatment period, three treatment cycles, and the follow up period. The number of participation days varies according to the menstrual cycle.

8. Study Drug Management

8.1. Study Drug

Generic name: Kamishoyosan.
Chemical Name : None
Each 7.5 g contains 4.0 g of dried extract from the following mixed crude drugs: JP Bupleurum Root 3.0 g; JP Peony Root 3.0 g; JP Atractylodes Lancea Rhizome 3.0 g; JP Japanese Angelica Root 3.0 g; JP Poria Sclerotium 3.0 g; JP Gardenia Fruit 2.0 g; JP Moutan Bark 2.0 g; JP Glycyrrhiza 1.5 g; JP Ginger 1.0 g; and JP Mentha Herb 1.0 g.
Excipients: JP magnesium stearate and JP lactose hydrate.
Dosage form: granules.
Storage: to preserve product quality, protect from moisture and direct sunlight and store in a cool place.
Lot number: recorded at manufacture.

8.2. Management of Study Drug

8.2.1. Procedures for Receiving Investigational Medicinal Products

After approval by the Certified Review Board and on or after the study start date specified in the agreement with the study-drug provider, the study drug will be supplied to the study institution.

1）The investigational drug supplier shall deliver the investigational drug to the participating medical institution on the desired delivery date, accompanied by an "Investigational Drug Delivery Form."

2）A person designated by the Investigational Drug Manager (hereinafter referred to as the "Investigational Drug Manager, etc.") will prepare an "Investigational Drug Storage and Management Sheet" prior to the delivery of the investigational drug.

3）The Investigational Drug Manager, etc., will verify the quantity of the delivered investigational drug, check for any contamination or damage, and confirm the "Investigational Drug Delivery Note."

4）The Investigational Drug Manager, etc., will prepare a "Receipt for Investigational Drug" bearing a seal or signature and submit it to the Investigational Drug Supplier. In addition, the Principal Investigator shall

retain the "Investigational Drug Supply Form."
5）After confirming that there are no issues with the receipt of the investigational drug, the Investigational Drug Administrator or equivalent will enter the required information into the "Investigational Drug Storage and Management Form" and manage the receipt and issuance of the investigational drug.

8.2.2. Storage of Investigational Drugs
The Investigational Drug Manager or other designated personnel shall store the investigational drug appropriately in accordance with the storage instructions.

8.2.3. Management of Investigational Drugs
The Investigational Drug Manager and others will manage "unused investigational drugs*" and "used investigational drugs**" separately. Furthermore, investigational drugs must not be used for purposes other than this study (such as other clinical studies, animal experiments, or basic research).

*Unused Investigational Drug: Refers to drugs that have not been issued (drugs that have not been prescribed).
**Used Investigational Drug: Refers to drugs that have been issued (including those returned because they were not taken).

8.2.3.1. Procedure for Returning Investigational Drugs
"Unused Investigational Drugs" and "Used Investigational Drugs" will be returned to the investigational drug supplier according to the following procedures.
1）As a general rule, the investigational drug administrator or designated personnel will store unused investigational drugs separately from other investigational drugs until the study is completed. As a general rule, used investigational drugs will be stored until the study is completed and will not be discarded. The outer packaging of used investigational drugs may be discarded as needed.
2）The research drug administrator or designated personnel will provide the research drug supplier with a signed and stamped or signed "Unused Investigational Drug Return Form," the unused investigational drugs, the "Used Investigational Drug Return Form," and the "Investigational Drug Storage and Management Sheet (Copy)" to the investigational drug supplier (even if there are 0 packages of unused or used investigational drugs, prepare the Unused Investigational Drug Return Form and Used Investigational Drug Return Form, entering 0 for the number of drugs and boxes).

8.2.4. Issuance of Investigational Drugs and Supervision
1）The Investigational Drug Administrator or other designated personnel will issue investigational drugs in accordance with the prescription instructions of the Principal Investigator or Sub-Investigator. At that time, the necessary details must be recorded on the "Investigational Drug Storage and Management Sheet."
2）If, upon dispensing the investigational drug, the Investigational Drug Manager, etc discover any deviations from the study protocol or any ambiguities, they shall promptly notify the Principal Investigator or Sub-Investigator of this fact and dispense the investigational drug only after the issue has been resolved.
3）The Investigational Drug Manager, etc will provide appropriate guidance to study participants regarding the method of administration, what to do if a dose is missed, how to store the investigational drug, and the return of the investigational drug (including reporting in the event of loss or disposal).
4）The Investigational Drug Manager, etc will explain to study participants that empty aluminum heat-sealed pouches may be disposed of appropriately as they are used.

9. Discontinuation Criteria
If any of the following occurs after the start of the treatment period, the Principal Investigator or Sub-Investigator will discontinue study participation and perform the specified discontinuation examinations. When discontinuation is due to a safety issue such as an adverse event, appropriate treatment will be provided immediately and follow-up will continue, where possible, until recovery.

1. The participant requests withdrawal from the study or withdraws consent.
2. It becomes apparent after registration that eligibility criteria were not met.
3. Continuation is judged undesirable because the underlying condition has worsened.
4. Continuation becomes difficult because of worsening comorbidity.
5. Continuation becomes difficult because of an adverse event.
6. Continuation becomes impossible because of participant circumstances such as relocation.
7. Pregnancy is confirmed.
8. The study as a whole is discontinued.
9. The Principal Investigator or Sub-Investigator considers discontinuation appropriate for another reason.

10. Observation/Test Items and Schedule

10.1. Test/Observation Items

10.1.1. Study Participant Background, information on the underlying disease, demographic and other characteristics

The following data will be collected during the pre-treatment period.

1） Subject Background: Date of informed consent, age at the time of informed consent, history of allergies, age at menarche, work schedule

2） Information on the underlying condition: Diagnosis, date of diagnosis, menstrual cycle

3） Patient Characteristics (stiff shoulders, headaches, dizziness, burning sensation, paroxysmal sweating, sensitivity to cold, constipation, skin problems, fatigue, diet, exercise, alcohol consumption, sleep)

4） Medical History and Comorbidities: The medical history will be investigated for clinically significant diseases or symptoms that had resolved or ceased prior to obtaining informed consent and are relevant to the safety evaluation. Complications refer to symptoms or diseases present at the time of obtaining informed consent. Clinically significant abnormal findings identified during pre-enrollment observations or examinations will be classified as complications at the discretion of the Principal Investigator or a Sub-Investigator.

5） Prior Medications/Therapies: This refers to all medications or treatments administered or performed prior to the start of the treatment period. Medications and therapies—including those prescribed by a physician or over-the-counter medications purchased by the study participant—administered prior to the start of the treatment period must be identified and recorded. Note that all medications and therapies for the underlying disease must be collected.

10.1.2. Physical Findings

Physical examinations are performed during pretreatment, on the first day of menstruation within +14 days in cycles 2, 3, and 4, or discontinuation. Physical examinations should also include an assessment of the presence or absence of edema. After treatment begins, findings are compared with pretreatment findings; clinically significant abnormalities are reported as adverse events.

10.1.3. Height, Weight, Vital Signs, and BMI

Height, weight, and BMI are measured during pretreatment and recorded to one decimal place. Blood pressure and pulse are measured during pretreatment; on the first day of menstruation within +14 days in cycles 2, 3, and 4; or discontinuation, after at least 5 minutes of rest in the sitting position. Clinically significant abnormalities after treatment initiation are reported as adverse events.

10.1.4. Blood Tests

AST, ALT, gamma-GTP, sodium, and potassium are measured during pretreatment, on the first day of menstruation within +14 days in cycle 4, or at discontinuation. Clinically significant abnormalities after treatment initiation are reported as adverse events. Blood samples are disposed of promptly according to institutional procedures after measurement.

10.1.5. Menstrual Distress Questionnaire (MDQ)

The MDQ measures physical and psychological complaints associated with the menstrual cycle and consists of eight subscales: pain, concentration, behavioral change, autonomic nervous system, fluid accumulation, negative feeling, hyperthymia, and control. Assessments are made for three periods: menstruation, post-menstruation, and premenstruation. During pretreatment, participants retrospectively rate the preceding menstrual cycle using a paper questionnaire at the hospital. In cycles 2, 3, and 4, participants retrospectively rate the preceding cycle using an online questionnaire at home on the first day of menstruation (Table10-1). At discontinuation, the most recent applicable period is evaluated.

Table 10-1 Menstrual Cycle Under Evaluation

Date of Evaluation	Menstrual Cycle Being Evaluated	Evaluation Method
－1: At the time of the visit during the cycle	– Cycle 2	Paper Form (Hospital)
Start date of menstruation in the second cycle	First Cycle	Online
Start date of menstruation in Cycle 3	Second cycle	Online
Start date of menstruation in Cycle 4	Third cycle	Online

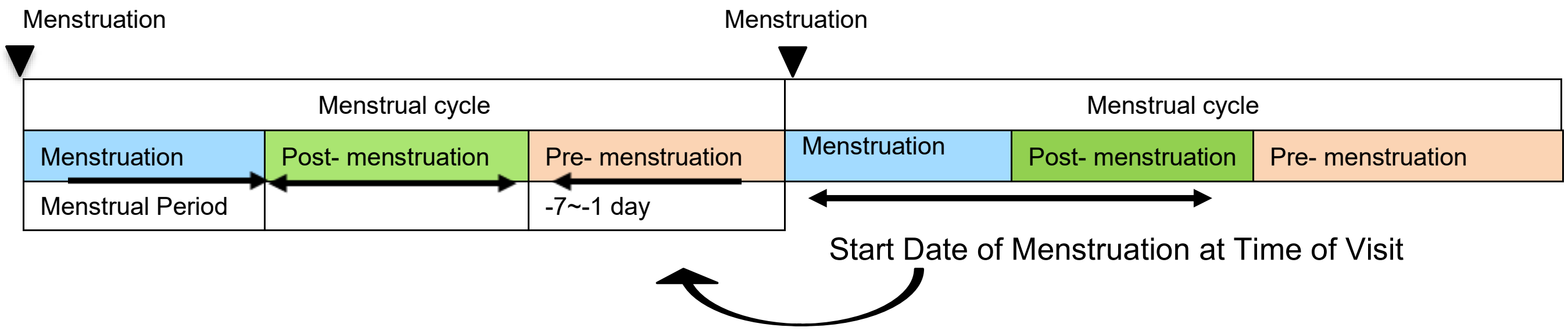

On the first day of menstruation, recall the “during-menstruation,” “post-menstruation,” and “pre-menstruation” phases of the previous menstrual cycle.
The pre-treatment period is conducted at the clinic, and the treatment period is conducted at home.

10.1.6. Work Productivity

The Work Productivity and Activity Impairment Questionnaire (WPAI) assesses time missed from work because of health problems, time missed for other reasons, time worked, the degree to which health problems affected work productivity, and the degree to which health problems affected regular non-work activities. During pretreatment, the preceding 7 days are assessed at the hospital visit. During treatment and post-treatment observation, the preceding 7 days are assessed online at home on the first day of menstruation in cycles 2, 3, and 4, or at discontinuation.

10.1.7. Coldness Score

During pretreatment, coldness is assessed at the visit. In cycles 2, 3, and 4, participants use an online questionnaire at home on the first day of menstruation to retrospectively rate subjective coldness during the premenstrual period of the preceding cycle on an 11-point scale from 0 to 10. At discontinuation, the most recent applicable period is assessed.

10.1.8. Stiff-Shoulder Score

Stiff shoulders are assessed using the same timing and 0–10, 11-point scale as the coldness score.

10.1.9. Overall Impression After Study Participation

On the first day of menstruation in cycle 4, or at discontinuation, participants assess their overall impression of changes in PMS symptoms after study participation at home using an online questionnaire and a 7-point Likert scale.

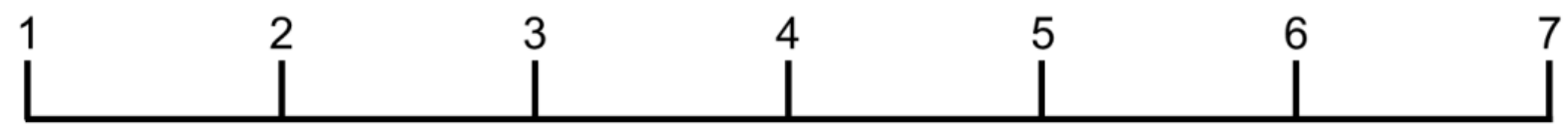

1 Very much improved, 2 Much improved, 3 Minimally improved, 4 No change, 5 Minimally worse, 6 Much worse, 7 Very much worse

10.1.10. Symptom Assessment and Menstrual-Day Recording Using YaDoc

Using the YaDoc smartphone application, participants rate Kampo-related symptoms such as irritability and emotional instability, menstruation-associated symptoms such as abdominal bloating and fatigue, and sleep status such as sleep satisfaction and daytime sleepiness on an 11-point scale from 0 to 10. Menstrual dates and whether menstruation is ongoing are also recorded. Assessments begin at registration and continue daily, once per day and preferably at approximately the same time, through the end of cycle 3. If assessment cannot begin on the day of registration, it begins as promptly as possible before the treatment period.

10.1.11. Assessment Using Fitbit

Fitbit is used to collect information on heart-rate variability during sleep, heart rate, sleep duration, and sleep status. Participants wear Fitbit at bedtime every day from registration through the end of cycle 3. If collection cannot begin on the registration day, it begins as promptly as possible before treatment. Skin temperature during sleep, stress score, and blood oxygen data are also collected by the system but are not used in this study. These data are retained with the other data because they may be valuable in the future and may be secondarily used in another study as needed.

10.1.12. Concomitant Medications and Therapies

Information is collected on concomitant medications and therapies associated with adverse events and administered during treatment and post-treatment observation. Treatment for adverse events for which a causal relationship to the study drug cannot be ruled out is collected as concomitant medication or therapy.

10.2. Schedule of Assessments

The study consists of a pre-treatment period, treatment period, and follow up period. The detailed schedule in the Japanese source protocol specifies visits, informed consent/registration, participant background, disease information, anthropometrics, prior/concomitant therapy, study-drug administration, safety assessments, questionnaires, YaDoc assessments, and Fitbit assessments across cycle -1, cycles 1–3, cycle 4, and discontinuation. The timing windows and footnotes are retained in the Japanese source protocol.

	Pre-treatment	Treatment			Follow up	Discontinuation
Menstrual cycle	−1 cycle[1)]	1st cycle[2)]	2nd cycle	3rd cycle	4th cycle[3)] (End)	
Visit	Menstruation − First day	–	Menstruation − First day	Menstruation − First day	Menstruation − First day	Discontinuation
Allowance	＋14	–	＋14	＋14	＋14	Promptly
Visit the hospital	●		●[4)]	●[4)]	●[4)]	●[5)]
Obtaining Consent	●					

Registration						
Subject Background	●					
Information on Underlying Conditions	●					
Height, Weight, BMI	●					
Prior and Concomitant Medications/Therapies	←	—	—	—	—	→
Study Drug Administration [6]		←	—	→		
**Safety Assessment**						
Physical Findings	●		●	●	●	●
Blood Pressure, Pulse Rate	●		●	●	●	●
Blood Tests	●				●	●
Adverse Events		←	—	—	—	→
**Assessment via Questionnaire [7]**						
MDQ	●		●	●	●	●[8]
WPAI	●		●	●	●	●
Coldness Score	●		●	●	●	●[8]
Shoulder Stiffness Score	●		●	●	●	●[8]
Overall impression					●	●
**Evaluation via smartphone app (YaDoc)[9]**						
Sleep Assessment<br>Kampo medicine assessment<br>Evaluation of Menstrual-Related Symptoms	▲[10]	←	—	→		
**Evaluation via Fitbit (smartwatch) [11]**						
Sleep Assessment,<br>Heart Rate Assessment	▲[10]	←	—	→		

1) Starting from the menstrual cycle in which administration of the study drug begins (Cycle 1), informed consent will be obtained and eligibility confirmed during Cycle

2) The treatment period begins on the first day of menstruation in Cycle 1.

3) Participants must visit the clinic on the first day of menstruation in Cycle 4 (within +14 days) to undergo the end-of-study examination.

4) The patient must visit the clinic on the first day of menstruation in each cycle (within +14 days).

5) Visit the clinic upon discontinuation to undergo the prescribed examinations and observations.

[6] Study drug administration group only. Administration of the study drug begins on Day 1 of Cycle 1 and continues until the end of Cycle 3 (the day before Day 1 of Cycle 4).<br>[7] During cycles 2, 3, and 4 of the treatment phase, patients will be assessed via an online questionnaire at home on the first day of menstruation.<br>[8] At the time of discontinuation, the status during the most recent applicable period will be assessed.<br>[9] Until the end of the third cycle (the day before the first day of menstruation in the fourth cycle), evaluations will be conducted daily (once a day, at the same time whenever possible) using the smartphone app (YaDoc).<br>[10] Begin data collection and assessment as soon as possible after the enrollment date. If it is not possible to begin on the enrollment date, begin data collection and assessment as soon as possible before the start of the treatment period.<br>[11] Until the end of the 3 cycle (the day before the first day of menstruation in the 4 cycle), wear the Fitbit (smartwatch) every night at bedtime to conduct the assessment.

11. Adverse Event Assessment / Reporting

11.1. Definitions of Adverse Events and Adverse Drug Reactions

11.1.1. Adverse Events and Adverse Drug Reactions

An adverse event is any unfavorable medical occurrence in a patient or study participant administered a medicinal product, including the study drug, whether or not causally related to that medicinal product. It includes any unfavorable or unintended sign (including an abnormal laboratory change), symptom or disease occurring with administration, regardless of causal relationship. An adverse event for which a causal relationship to the study drug cannot be ruled out, including cases in which the relationship is unknown, is handled as an adverse drug reaction. An adverse event suspected to be caused by conduct of the study is handled as a disease or other event subject to reporting to the Certified Review Board.

11.1.2. Serious Adverse Events

- Results in death.
- Is life-threatening.
- Requires hospitalization for treatment or prolongation of hospitalization.
- Results in persistent or significant disability/incapacity.
- Results in a congenital anomaly.
- Other important event that, even if not immediately life-threatening or requiring hospitalization, jeopardizes the participant or requires intervention to prevent one of the outcomes listed above.

11.2. Adverse Event Reporting

11.2.1. Recording Adverse Events

Collection begins at the start of the treatment period and continues through the final visit. At visits, the Principal Investigator or Sub-Investigator confirms subjective symptoms and abnormal laboratory changes. The case report form records event name, onset date, severity, seriousness, relationship to study drug and rationale, action taken with study drug, outcome and outcome date.

11.2.2.1 Reporting Diseases and Other Events

1) When the Principal Investigator becomes aware of the onset of a disease or condition listed in “Table 11-1: Diseases and Conditions Subject to Reporting to the Certified Clinical Research Review Committee” that is suspected to be attributable to this study, the Principal Investigator will, in accordance with the “Procedures for Submitting Reports on Drug-Related Diseases and Conditions in Specified Clinical Research,” prepare the initial report consisting of the “Report on Drug-Related Diseases and Conditions (Unified Form 8)” and the “Detailed Description Form” within the reporting deadline stipulated by law, and report them to the Certified Review Board and the administrator of the participating medical institution, as well as to Tsumura & Co.

Table 11-1 Diseases or other events Subject to Reporting to the Certified

Predictability	Seriousness	Reporting deadline*1
Unknown / known	Death*2	15 days
Unknown	Disease/event that may lead to death*2	15 days
Unknown	Specified serious diseases/events including hospitalization/prolonged hospitalization, disability, risk of disability, comparably serious event, or congenital disease/abnormality in offspring	15 days
Unknown	Disease/event due to infection	15 days
Known	Death due to infection	15 days
Known	Specified serious disease/event due to infection	15 days
Known	Specified serious disease/event	30 days

(*1) Deadline for reporting to the Certified Review Board, the Principal Investigator, the administrator of the participating in medical institution, or the Minister of Health, Labor and Welfare.
(* 2) Cases that cannot be predicted based on the precautions for use (hereinafter referred to as "precautions for use, etc.") stated in the package insert, on the container, or on the packaging of the pharmaceuticals, etc., used in the specified clinical research, or cases that, although predictable based on the precautions for use, etc., of said pharmaceuticals, etc., for which the trend of occurrence cannot be predicted, or for which a change in the trend of occurrence indicates a risk of the occurrence or spread of harm to public health and hygiene.
2) The Principal Investigator shall promptly report to the administrator of the participating medical institution any unforeseeable deaths, disease or other events, that may lead to death, or other matters deemed serious, suspected to be attributable to the conduct of this study. The method of reporting in such cases is not specified.
3) If the Principal Investigator obtains new information regarding the disease or other events, listed in "Table 11-1: disease or other events, Subject to Reporting to the Certified Review Board," or if the outcome of a disease, etc., changes, the Principal Investigator shall prepare a supplementary report using the "Pharmaceutical Disease, etc., Report (Unified Form 8)" and the "Detailed Description Form," and report it to the Certified Review Board and the administrator of the participating medical institution.

<Principal Investigator Contact Information>
Tottori University Hospital, Department of Women's Medicine, Fuminori Taniguchi
Postal Code 683-8504 36-1 Nishimachi, Yonago City, Tottori
Phone: 0859-38-6647 / Fax: 0859-38-6649
<Holiday and Nighttime Contact Information>
Tottori University Hospital, Department of Women's Medicine
Tel: 0859-38-6913

11.2.2.2. Procedures for Reporting to the Ministry of Health, Labor and Welfare

1) When the Principal Investigator becomes aware of the onset of a disease or condition listed in "Table 11-1: Diseases and Conditions Subject to Reporting to the Certified Review Board" that is suspected to be attributable to this study and falls under the category of "Unknown," the investigator must follow the procedures outlined in "11.2.2.1. Procedures for Reporting Diseases and Conditions" and create an initial report via the "Disease Reporting" feature of the jRCT (System for Public Disclosure of Clinical Research Implementation Plans and Research Summaries) to create an initial report.

2) If the Principal Investigator obtains new information regarding matters subject to reporting to the Minister of Health, Labor and Welfare, or if the outcome of a disease or condition changes, the Principal Investigator

shall create a supplementary report via jRCT.

3) The Principal Investigator shall report via jRCT within the reporting deadlines stipulated by the laws and regulations listed in “Table 11-1: Diseases, etc., Subject to Reporting to the Certified Review Board.”

11.3. Periodic Reporting of Diseases or Other Events
1) Any diseases or conditions other than those listed in “Table 11-1: Diseases and Conditions Subject to Reporting to the Certified Clinical Research Review Committee” that are suspected to be attributable to the conduct of this study shall be subject to periodic reporting via the “Periodic Report (Standard Form 5).”

2) As a general rule, the Principal Investigator shall prepare a periodic report annually, starting from the date the research plan was submitted to the Minister of Health, Labor and Welfare, and within two months after the expiration of that period, prepare a periodic report and submit it to the accredited clinical research review committee and the administrator of the participating medical institution.

11.4. Provision of Information to Marketing Authorization Holders
The Principal Investigator shall provide information to any manufacturer or distributor of pharmaceuticals, etc., that manufactures or intends to manufacture and market investigational drugs subject to reporting requirements for adverse drug reactions, when requested to do so by such manufacturer or distributor.

12. Completion, Discontinuation, and Suspension of the Study
12.1. Study Completion
The study is completed when the study period has ended and all case report forms have been finalized. The Principal Investigator submits the final study report and summary with the Completion Notification (Unified Form 12) to the Certified Review Board for its opinion, and thereafter submits them without delay to the administrator of the study institution.

12.2. Study Discontinuation / Suspension
The Principal Investigator may discontinue or suspend the study if newly obtained information reveals a serious issue concerning study-drug safety or efficacy; if the Certified Review Board instructs a change to the study plan that is judged difficult to accept; or if the Board recommends or instructs discontinuation. If discontinuation is decided, the reason and subsequent actions are immediately communicated to study personnel. Participants are promptly notified regardless of reason and appropriate measures, and safety examinations are implemented. If the study is discontinued, the Principal Investigator submits a Discontinuation Notification (Unified Form 11) to the institution administrator and Certified Review Board.

13. Target Sample Size and Study Period
13.1. Target Sample Size
50 participants: 25 in the study-drug group and 25 in the observation group.

13.2. Study Period
April 2022 (after Certified Review Board approval) to March 2025. Registration deadline: March 2024.

14. Statistical Considerations
14.1. Analysis Populations
Full Analysis Set (FAS): participants registered in the study for whom the treatment period has begun, excluding participants found after registration to have a major protocol violation or to have been ineligible.
Safety analysis set: registered participants who entered the treatment period, excluding those with no safety data to analyze and those clearly not administered the allocated study drug.

14.2. Analysis Methods

14.2.1. Participant Background and Baseline

Participant background and baseline values will be described using appropriate summary statistics such as frequencies, proportions, and medians.

14.2.2. Primary Endpoint

Changes at each time point from baseline to the final assessment in total premenstrual MDQ score will be analyzed using a linear mixed-effects model for repeated-measures outcomes. Mixed effect Models for Repeated Measurements (MMRM) will be used with an unstructured correlation structure. Fixed effects will include comparison group (study-drug / observation), assessment time point, group-by-time interaction, and baseline MDQ total score. If regression parameters cannot be estimated under the unstructured model, the covariance structure will be changed in the following order: Toeplitz, Heterogeneous Compound Symmetry, Autoregressive(1), Compound Symmetry, and Variance Components. The principal assessment is the between-group difference in mean change in total MDQ score at the final time point estimated by MMRM. If regression parameters cannot be estimated under any model, the endpoint will be evaluated using two-sample t-test as stated in the Japanese source protocol. The significance level is two-sided 5%. Secondarily, least-squares mean changes in MDQ total score by group at each cycle and the between-group difference in those mean changes will be estimated.

14.2.3. Secondary Endpoints

MDQ total scores for premenstruation, menstruation and post-menstruation and each subscale score will be analyzed using the same MMRM approach as Section 14.2.2. If regression parameters cannot be estimated under any model, evaluation will use two-sample t-test as stated in the Japanese source protocol, with a two-sided 5% significance level. Least-squares mean outcomes by group at each cycle and between-group differences will be estimated. For sleep, Kampo medicine, menstruation-associated symptoms, work productivity and overall impression after study participation, basic statistics will be summarized by group and observation cycle, and between-group comparisons will be made for values at each time point or change from baseline. Fitbit sleep and heart-rate outcomes will be summarized by group and cycle and compared between groups. Adverse events will be summarized by event, severity and seriousness using frequencies and proportions.

14.2.4. Subgroup Analyses

To explore differences in effect associated with patient background or characteristics related to use of kamishoyosan, subgroup analyses of primary and secondary endpoints will be performed by work schedule (night shift: yes/no) and by presence/absence of each patient characteristic specified in Section 10.1.1.

14.3. Rationale for Sample Size

Based on expected clinical efficacy estimates in the same disease area, the between-group mean difference for the primary endpoint was assumed to be 6.0 and the standard deviation 6.73. A sample size of 21 participants per group was required to achieve 80% power at a two-sided 5% significance level. Allowing for discontinuations and other losses, the target was set at 25 per group, 50 overall.

15. Direct Access to Source Documents

15.1. Scope of Source Documents

Source documents are documents, data and records underlying case report forms and include consent documents, medical records, nursing records, laboratory data, study-drug administration records, questionnaires and other records needed to reconstruct and evaluate study conduct.

15.2. Items Treated as Source Data in the Case Report Form

1) Data collected using ePRO, YaDoc and Fitbit.
2) Causal relationship of adverse events to study drug and comments.
3) Reasons for discontinuation and comments.
4) Comments of the Principal Investigator or Sub-Investigator.

15.3. Assurance of Direct Access

The Principal Investigator will make all study-related records, including source documents, available for direct access and cooperate when monitoring or audit is performed or when an investigation is conducted by the Certified Review Board.

16. Quality Control / Quality Assurance

The Principal Investigator will apply quality control at each stage of data handling to assure the reliability and proper processing of all study-related data. No audit will be conducted in this study. If noncompliance with applicable laws or the protocol becomes known, the Principal Investigator will promptly report it to the administrator of the study institution. Particularly serious noncompliance will be referred promptly to the Certified Review Board for its opinion and necessary action.

16.1. Monitoring

Monitoring is an investigation performed by a person designated by the Principal Investigator to ensure appropriate conduct of the clinical study, from the perspectives of data reliability and protection of study participants, by reviewing study progress and compliance with applicable laws and the protocol.

16.2. Monitoring Methods

In this study, monitoring will be conducted via central monitoring. Furthermore, in the event of the occurrence of a disease or other condition subject to reporting to the Certified Review Board, or in the event of a serious noncompliance with laws and regulations or the research protocol, on-site and off-site monitoring will be conducted in combination as necessary. Additionally, the monitor will submit a monitoring report to the Principal Investigator within two weeks of completing the monitoring.

16.2.1. Preparation for Monitoring

Prior to conducting monitoring, the monitor will perform the following checks and other necessary procedures.

1) Confirmation of Monitoring Procedures

The monitor will consult with the Principal Investigator and others regarding the monitoring activities for the study in question in accordance with the monitoring plan, and confirm the procedures for conducting the monitoring.

2) Confirmation of Research Procedures

Confirm the procedures from case registration through completion, as well as the roles of those involved in the study.

3) Consultation on Items to Be Monitored

The monitor will consult with the Principal Investigator and others regarding the items to be monitored.

4) Obtaining Materials Necessary for Monitoring

The monitor will obtain the materials necessary for conducting monitoring from the Principal Investigator and others.

16.2.2. Monitoring at the Start of the Study

The monitor will conduct the monitoring described in items 1) through 4) below at the time of the first subject’s enrollment.

1) Confirmation that the Certified Review Board has approved the conduct of the specified clinical research in question.

2) Confirmation that the administrator of the participating medical institution has approved the conduct of the specific clinical research in question.

3) Confirmation that the date on which consent was obtained from the registered research subject is the same as or prior to the registration date.

4) Confirmation that the registered research subject meets the inclusion criteria specified in the research protocol and does not violate the exclusion criteria.

16.2.3. Monitoring During the Study

The monitor will conduct monitoring, in principle, every six months starting from the enrollment of the first subject during the study.
Monitoring will cover all cases. Additionally, monitor will be conducted as needed in the adverse event or other conditions subject to reporting to the Certified Review Board, or in the event of serious noncompliance.

1） Verification of Enrollment Progress and Data Collection Pace
2） Verification of the Submission Status of Case Report Forms
3） Verification of omissions (blank fields) in case report forms and review of reported content
4） Verification of participation status for enrolled study subjects
5） Verification of the occurrence of adverse events
6） Verification of deviations and non-conformities
7） Evaluation status via the smartphone app (YaDoc) and Fitbit (smartwatch)

16.2.4. Monitoring at the End of the Study

The monitor will perform the following monitoring activities upon study completion:

1） Confirmation that all inquiries have been answered
2） Confirmation that all data has been locked

17. Ethical Considerations

17.1. Applicable Rules

All investigators will conduct the study in accordance with the Declaration of Helsinki (World Medical Association, adopted in 1964 and subsequent amendments), the Clinical Trials Act (Act No. 16 of 2017), the Enforcement Regulation of the Clinical Trials Act (Ministry of Health, Labour and Welfare Ordinance No. 17 of 2018), and related laws and regulations.

17.2. Periodic Reporting to the Administrator of the Study Institution

At least annually, the Principal Investigator submits information on progress, adverse events, disease or other medical events, noncompliance with rules or protocol, safety and scientific validity, involvement of pharmaceutical manufacturers and other problems to the administrator and Certified Review Board, and obtains review of whether the study may continue.

17.3. Protection of Personal Information and Privacy

Study personnel comply with applicable laws and ordinances on personal information protection and make every effort to protect personal information and privacy. Information learned through the study must not be disclosed without legitimate reason, including after leaving the position. This requirement will remain in effect even after study personnel have left their positions. The Principal Investigator or Sub-Investigator removes descriptions that alone would immediately identify a participant, such as name, initials, address, telephone number and medical-record number, and performs anonymization. Participant identification codes or registration numbers are used for registration and case report forms. The correspondence table is stored and managed by the Principal Investigator according to Section 21.1.
YaDoc and Microsoft Forms comply with the “three ministries/two guidelines” framework for protection of medical information. YaDoc implements TLS 1.2 encryption. Integrity Healthcare Co., Ltd., developer of YaDoc for this study, integrates and provides information stored in the databases under appropriate data management as an external vendor. Only information processed so that individuals are not identified is registered in each database, and the integration/provision process and final provided data contain no information that can identify an individual participant. When publishing study information, investigators take sufficient care so participants cannot be identified.

17.4. Benefits of Participation
Participation may relieve distress caused by premenstrual symptoms through study-drug administration. Confirmation of efficacy and safety may broaden future treatment options and contribute to improved quality of life and establishment of effective PMS treatment. The observation group receives no direct therapeutic benefit from study-drug administration, but can receive non-pharmacological care such as counseling and lifestyle guidance. Detailed examinations may also help characterize the participant's condition and inform subsequent care.

17.5. Safety and Disadvantages of Participation
Kamishoyosan is an approved pharmaceutical product and is administered within the approved dosage and administration range, but experience in the target disease is limited and adverse reactions may occur as described in Section 1.3. To minimize adverse-event risk, eligibility criteria, prohibited concomitant medications/therapies, examinations/observations and monitoring are specified. Regular monitoring is required after study initiation. Blood collection and assessments are performed while observing participant condition. The observation group has no risk of study-drug-related injury, but treatment options for PMS are restricted during the study because certain prior/current therapies (oral contraceptives, sex hormone preparations, antidepressants, anxiolytics, sleeping pills, or Kampo medicine) are excluded or prohibited. Alternative treatments are explained so that participation remains voluntary.

17.6. Post-study Care
At study completion, the participant's condition will be observed and the best available treatment will be provided.

18. Study Costs
18.1. Funding Sources and Financial Relationships
The study receives funding, study drug, the questionnaire system used for assessment, and Fitbit smartwatches from Tsumura & Co. Tsumura provides information concerning the study drug but is not involved in conduct of the study or analysis. Potential conflicts of interest concerning conduct and results are managed in accordance with the conflict-of-interest management policy and reviewed by the Tottori University Hospital Certified Review Board before study initiation. Study reports, safety information and problems associated with study conduct are reported to Tsumura & Co.

18.2. Study Costs and Participant Expenses
Since this study will be conducted within the scope of routine medical care, the health insurance to which the research participants are enrolled will cover the medical expenses for treatments provided to them during the study period, and the participants will be responsible for their copayments. However, the costs of clinical laboratory tests will be covered by research funds. In addition, the investigational drug will be provided by Tsumura Co. Data communication fees for smartphone use will be borne by the research participants. To help alleviate the financial burden on research participants, 6,000 yen will be paid for the first visit, and 2,000 for each subsequent scheduled visit. Furthermore, the Fitbit (smartwatch) will be provided to the research participant upon completion of the study (except in cases of voluntary withdrawal).

18.3. Compensation for Health Injury
If health injury attributable to study conduct occurs, the Principal Investigator will ensure prompt access to appropriate diagnosis, treatment and necessary measures. Insurance is maintained as compensation for study-related health injury, covering compensation for death/residual disability, medical expenses and medical allowance. Medical expenses/allowance are compensated only for known or unknown adverse drug reactions attributable to study-drug administration and severe enough to require inpatient treatment.

19. Protocol Approval, Revision, and Deviations
19.1. Approval and Revision

Before study initiation, the Principal Investigator submits the protocol to the Certified Review Board and obtains approval from the Board and the administrator of the study institution. When the protocol is revised, the decision is promptly communicated to study personnel, and the revised protocol must be submitted to and approved by the Board. For a major change, temporary suspension of enrollment is considered from the perspective of participant protection.

19.2. Deviations from the Protocol

The following provisions apply to deviations from the research protocol and applicable laws and regulations.

1) The Principal Investigator or Sub-Investigator must not deviate from or change the protocol before obtaining approval from the Certified Review Board and institutional administrator.

2) The Principal Investigator or Sub-Investigator may deviate from or modify the research protocol prior to obtaining prior approval from the Certified Review Board for unavoidable reasons, such as emergency avoidance. In such cases, the Principal Investigator or Sub-Investigator must promptly submit the details of the deviation or the details and reasons for the deviation or change, and, if a revision to the research protocol or other documents is necessary, promptly submit a draft of such revision to the Certified Review Board to obtain approval from both the Certified Review Board and the institutional administrator.

3) In the event of a deviation from the research protocol, the Principal Investigator or Sub-Investigator must record all details of the deviation and the reasons for it. All protocol deviations and reasons are recorded.

4) If noncompliance with laws or regulations is identified, the Principal Investigator promptly reports it to the institutional administrator and takes necessary action. Serious noncompliance is promptly referred to the Certified Review Board, and necessary preventive measures are implemented and communicated to study personnel.

20. Case Report Forms

Data are collected and case report forms are prepared and submitted using an Electronic Data Capture (EDC) system and an electronic patient-reported outcome (ePRO) system.

20.1. EDC System

TUH-RSS is used as the EDC system. After Certified Review Board approval and submission of a copy of the institutional approval notification to the Study Secretariat, the Data Center provides individual IDs and passwords to the Principal Investigator, Sub-Investigators and study collaborators. Creation, submission, correction and viewing of case report forms are performed through EDC; users are authenticated by ID/password and operations and the operator are electronically recorded. Case-report-form data are stored electronically in the EDC database. The Principal Investigator or Sub-Investigator enters protocol-specified data for all participants. Data managers review entered data and issue EDC queries as needed; investigators review queries and correct data where necessary.

20.2. ePRO and Questionnaire Surveys

20.2.1. Operation of ePRO and Questionnaires

Data are collected using ePRO, YaDoc, Fitbit and paper questionnaires. Systems of Integrity Healthcare Co., Ltd. are used. ePRO requirements are configured by Integrity Healthcare. Participants download YaDoc to their smartphones. Fitbit is supplied by Tsumura & Co. and connected to the participant smartphone by Bluetooth.

20.2.2. Preparation of Case Reports

1) Participants enter the data items specified in the study protocol directly into the ePRO or the smartphone app (YaDoc). As for the Fitbit (smartwatch), participants wear it every night when they go to sleep. Paper-based questionnaires are self-administered by participants.

2) ePRO and paper-questionnaire data are stored on Microsoft database servers through a system built with Microsoft Forms; YaDoc data are stored on its database server built on AWS (Amazon Web Services); Fitbit data are stored in the Fitbit database through the Bluetooth-connected smartphone.

3) Data managers will review the data as appropriate and, if necessary, contact the Principal Investigator or Sub-Investigator. The Principal Investigator or Sub-Investigator will review the inquiry.
4) Data stored in Integrity Healthcare databases are received by the Principal Investigator as external data at study completion.

20.3. Case Report Form Data Lock and Storage
After final data entry and corrections are complete, case-report-form data, including correction records, are locked as a dataset and appropriately retained under the responsibility of the Principal Investigator for the protocol-specified retention period.

21. Information Storage and Management
21.1. Storage and Management
The Principal Investigator will store documents related to the conduct of the study (copies of application documents, notification letters from the administrators of participating medical institutions, copies of various application forms and reports, consent forms, case reports, correspondence logs, and other documents or records necessary to ensure the reliability of the data) in a lockable locker at the Office of the Department of Women’s Medicine, Tottori University Hospital. Furthermore, electronic data will be managed with passwords using computers accessible only to personnel involved in this study. The retention period will be from the date of commencement of the study until five years have elapsed. After the retention period ends, paper documents will be shredded and disposed of. Other media will be anonymized and disposed of using appropriate methods.

21.2. Secondary Use of Information
Information collected for this study may in the future be used for a separate research purpose when an important investigation not currently planned or anticipated becomes necessary. Such secondary use will be reviewed and approved by an Ethics Review Committee or Certified Review Board and, where possible, explained again to participants with consent obtained. If explanation is not possible, study information will be disclosed in accordance with applicable guidelines. Information used in this way will not include information that can identify individuals.

22. Registration of Study Summary and Results
Before study initiation, information on this clinical study will be registered and disclosed in the Japan Registry of Clinical Trials (jRCT), the database established by the Ministry of Health, Labour and Welfare. Registered information will be updated as appropriate according to protocol changes and study progress, and study results will be registered without delay after study completion.

23. Ownership and Publication of Study Results
Although patent rights are unlikely to arise from this study, any intellectual property arising from study results will belong to Tottori University and Tsumura & Co. Results will be presented at scientific meetings and published in academic journals from 2024 after study completion. If a researcher other than the Principal Investigator presents or publishes results, arrangements will be determined through consultation with the Principal Investigator.

24. Study Organization
24.1. Principal Investigator
The Principal Investigator supervises the operations of the study institution and manages/directs the team of Sub-Investigators and study collaborators.
Fuminori Taniguchi, Professor, Department of Women’s Medicine, Tottori University Hospital
36-1 Nishi-cho, Yonago, Tottori 683-8504
TEL 0859-38-6647.

24.2. Study Secretariat

Provides support to the lead investigator and performs secretariat duties.
Contact: Yusuke Endo
Advanced Medicine, Innovation and Clinical Research Center, Tottori University Hospital
36-1 Nishimachi, Yonago City, Tottori 683-8504
TEL 0859-38-6946 / FAX 0859-38-6947.

24.3. Data Manager
Performs case registration, data management and Data Center operations.
Contact: Hiroshi Sunada
Advanced Medicine, Innovation and Clinical Research Center, Tottori University Hospital
36-1 Nishimachi, Yonago City, Tottori 683-8504
TEL 0859-38-6946 / FAX 0859-38-6947.

24.4. Monitor
Performs monitoring for this study.
Contact: Hiroshi Sunada
Advanced Medicine, Innovation and Clinical Research Center, Tottori University Hospital
36-1 Nishimachi, Yonago City, Tottori 683-8504
TEL 0859-38-6946 / FAX 0859-38-6947.

24.5. Statistical Analyst
Perform statistical analysis for this study.
Contact: Hisashi Noma, Department of Data Science, The Institute of Statistical Mathematics, The Graduate Institute for Advanced Studies, The Graduate University for Advanced Studies (SOKENDAI) and Research Advisor (Biostatistics), Tottori University Hospital
10-3 Midori-cho, Tachikawa, Tokyo 190-8562
TEL 050-5533-8440 / FAX 042-526-4339.

24.6. Research and Development Planning Support
Supports development of decision criteria at each stage based on study-drug characteristics and preparation of the basic framework of the protocol.
Contact: Yusuke Endo
Advanced Medicine, Innovation and Clinical Research Center, Tottori University Hospital
36-1 Nishimachi, Yonago City, Tottori 683-8504
TEL 0859-38-6946 / FAX 0859-38-6947.

24.7. Collaborating Research Organization
Participates in study-plan discussion and provides study funding, information related to the study drug and safety information. Tsumura & Co. is not involved in study conduct or analysis.
Tsumura & Co., Kampo Research and Development Division,
2-17-11 Akasaka, Minato-ku, Tokyo 107-8521
TEL 03-6361-7188 / FAX 03-5574-6664.
Person responsible: Akihito Konda, Executive Officer and Head of Kampo Research and Development Division.

24.8. External Contractor
Integrity Healthcare Co., Ltd. designs the ePRO system and manages the database.
Address: YAMATO B.L.D, 1-27-5 Nihonbashi-Kakigaracho, Chuo-ku, Tokyo 103-0014
TEL 03-6661-0858.
Person responsible: Ai Sonoda, President and Representative Director.

[Methods for Supervising Contractors]

When study work is outsourced, the contractor must implement appropriate safeguards for personal and confidential information in accordance with the Act on the Protection of Personal Information and other applicable requirements. The commissioning party may conduct audits when necessary; if safeguards are insufficient, corrective action is recommended and, if not improved, measures including termination of the outsourcing agreement may be considered.

25. References

1. Japanese Society of Obstetrics and Gynecology (ed.). Glossary of Obstetrics and Gynecology, revised 4th ed. 2018.
2. American College of Obstetricians and Gynecologists. Premenstrual syndrome. Guideline for Women's Health Care. A Resource Manual, Fourth Edition. 2014:607-613.
3. Steiner M et al. The premenstrual symptoms screening tool (PSST) for clinicians. Arch Women's Ment Health. 2003;6:203-209.
4. Takeda T et al. Prevalence of premenstrual syndrome and premenstrual dysphoric disorder in Japanese high school students. Arch Women's Ment Health. 2010;13:535-537.
5. Health and Global Policy Institute. Survey on Promotion of Working Women's Health. 2018.
6. RCOG Green-top Guideline. BJOG. 2016;No.48.
7. Japan Society of Obstetrics and Gynecology/Japan Association of Obstetricians and Gynecologists (eds.). Guidelines for Gynecological Practice, Outpatient Edition 2020. 2020:174-176.
8. Ministry of Health, Labour and Welfare, Pharmaceutical and Food Safety Bureau. Standards for Approval of OTC Kampo Products. September 30, 2008.
9. Ministry of Health, Labour and Welfare. Guidance for Preparation of Examination Questions. March 2018.
10. Iizuka S et al. Progress in Obstetrics and Gynecology Kampo Research No.16. Shindan to Chiryo Sha. 1999:37.
11. Mizowaki M et al. Anxiolytic effect of Kami-Shoyo-San (TJ-24) in mice: possible mediation of neurosteroid synthesis. Life Sci. 2001;69(18):2167-2177.
12. Iijima O et al. Jpn Pharmacol Ther. 1995;23(suppl.7):1813.
13. Kuboniwa H et al. Jpn Pharmacol Ther. 1999;27(suppl.6):1413.
14. Yamada K et al. Effectiveness of kamishoyosan for premenstrual dysphoric disorder: open-labeled pilot study. Neurosci. 2007;61(3):323.
15. Takamatsu K et al. A Multicenter, Randomized, Double-Blind, Placebo-Controlled Trial to Investigate the Effects of Kamishoyosan, a Traditional Japanese Medicine, on Menopausal Symptoms: The KOSMOS Study. Evid Based Complement Alternat Med. 2021;2021:8856149.
16. TSUMURA Kamishoyosan Extract Granules for Ethical Use, Interview Form. November 2020, 9th revision.
17. Moos et al. The development of a menstrual distress questionnaire. Psychosom Med. 1968;30:853-868.
18. Hori H et al. Psychological Measurement Scale Collection III. Science-sha. 2011:272.
19. Nagatsuka M. Premenstrual syndrome. Acta Obstet Gynaecol Jpn. 2009;61(12).
20. Berger D et al. Efficacy of Vitex agnus castus L. extract Ze 440 in patients with pre-menstrual syndrome (PMS). Arch Gynecol Obstet. 2000;264:150-153.
21. Kim HW et al. Intake of dietary soy isoflavones in relation to perimenstrual symptoms of Korean women living in the USA. Nurs Health Sci. 2006;8(2):108-113.
22. Momoeda M et al. Efficacy and safety of Vitex agnus-castus extract for treatment of premenstrual syndrome in Japanese patients: a prospective, open-label study. Adv Ther. 2014:362-373.
23. Taniguchi F et al. Efficacy of Tokishakuyakusan add-on therapy with low-dose oral contraceptive pills on endometriosis patients with dysmenorrhea. J Obstet Gynaecol Res. 2020;46(11):2280-2286.
