# Supplementary material for: A Kampo medicine, kamishoyosan, ameliorates menstruation-associated symptoms in patients with premenstrual syndrome: An exploratory open-label, randomized controlled trial
Source: PLoS One. 2026 Nov 6;21(11):e0362303. doi: 10.1371/journal.pone.0362303 (PMC13742958; doi:10.1371/journal.pone.0362303)
Supplement: Supplementary file 4 [file pone.0362303.s006.docx]

**Response to the Journal Requirements**

1. Please ensure that your manuscript meets PLOS ONE's style requirements, including those for file naming.

**Response#1**

Thank you for your careful review. We confirm that the manuscript has been carefully checked and revised to comply with all PLOS One style requirements, including the guidelines for file naming.

2. We note that the original protocol that you have uploaded as a Supporting Information file contains an institutional logo. As this logo is likely copyrighted, we ask that you please remove it from this file and upload an updated version upon resubmission.

**Response#2**

Thank you for your careful review and for pointing this out. Since there is no institutional logo, we have removed the Fitbit photograph from the original protocol file in Supporting Information. The revised version without the Fitbit photograph has been uploaded with the resubmission.

3. We note that the grant information you provided in the ‘Funding Information’ and ‘Financial Disclosure’ sections do not match.

When you resubmit, please ensure that you provide the correct grant numbers for the awards you received for your study in the ‘Funding Information’ section.

**Response#3**

We have matched the Financial Disclosure with the funding information in the manuscript.

We have corrected the Financial Disclosure section to match the manuscript as follows:

“This study was supported by a research grant from TSUMURA & CO (Tokyo, Japan). KSS extract granules for ethical use and smartwatch (Fitbit Versa 3) were provided by TSUMURA & CO.”

Details of the Funded study

•Initials of the authors who received each award→The corresponding author (FT) received research funding from TSUMURA & CO.

• Grant numbers awarded to each author→There is no grant number

• The full name of each funder→TSUMURA & CO.

• URL of each funder website→https://www.tsumura.co.jp/

Did the sponsors or funders play any role in the study design, data collection and analysis, decision to publish, or preparation of the manuscript?
→Yes. Conceptualization; Writing–original draft; Writing–review and editing

4. Thank you for stating the following financial disclosure: [Research grant was given by TSUMURA & CO (Tokyo, Japan), a pharmaceutical company.

This sponsor supported the study conceptualization, writing-review, and editing].

Please state what role the funders took in the study. If the funders had no role, please state: ""The funders had no role in study design, data collection and analysis, decision to publish, or preparation of the manuscript.""

If this statement is not correct you must amend it as needed.

Please include this amended Role of Funder statement in your cover letter; we will change the online submission form on your behalf.

**Response#4**

We confirmed role of the funder, role of funder statement was described in cover letter.

Role of Funder:

The funder (TSUMURA & CO., Tokyo, Japan) contributed to the Conceptualization and to the Writing–original draft; Writing–review and editing of the manuscript. The decision to publish was made by the authors in agreement with the funder. The funder had no role in data collection or data analysis.

5. Thank you for stating the following in the Competing Interests section: [YT, MF, and MY are employees of Tsumura & Co (Tokyo, Japan). The other authors have declared that no competing interests exist.].

Please confirm that this does not alter your adherence to all PLOS ONE policies on sharing data and materials, by including the following statement: ""This does not alter our adherence to PLOS ONE policies on sharing data and materials.” (as detailed online in our guide for authors http://journals.plos.org/plosone/s/competing-interests). If there are restrictions on sharing of data and/or materials, please state these. Please note that we cannot proceed with consideration of your article until this information has been declared.

Please respond by return email with your amended Competing Interests Statement and we will change the online submission form on your behalf.

**Response#5**

We confirm that the declared competing interests do not alter our adherence to all PLOS ONE policies on sharing data and materials. We have revised the Competing Interests Statement as follows.

Competing Interests:

This does not alter our adherence to PLOS ONE policies on sharing data and materials. (P29, L461)

A Data Availability Statement:

The data underlying this study are not publicly available because they contain potentially identifiable patient information. Data are available from the Advanced Medicine, Innovation and Clinical Research Center, Tottori University Hospital (contact via crdm@ml.med.tottori-u.ac.jp) for researchers who meet the criteria for access to confidential data. (P30, L470)

6. If the reviewer comments include a recommendation to cite specific previously published works, please review and evaluate these publications to determine whether they are relevant and should be cited. There is no requirement to cite these works unless the editor has indicated otherwise.

**Response#6**

The reviewers had no recommendations for citations from specific articles.

**List of Point-by-Point Response to the Reviewer’s Comments**

**Response to the comments of reviewer #1:**

**Comment #1**

1- Multiplicity – Numerous secondary and subscale analyses were conducted without adjustment for multiple comparisons, increasing the risk of false-positive findings.

**Response#1**

We agree that multiple secondary and subscale analyses increase the risk of false-positive findings. However, these analyses were prespecified as exploratory and were not intended to provide confirmatory evidence. Consistent with regulatory guidance, multiplicity adjustment is primarily required when multiple endpoints are used to support confirmatory conclusions or treatment claims, whereas exploratory endpoints may be reported without multiplicity adjustment if interpreted appropriately. Therefore, we have clarified that no multiplicity adjustment was performed for secondary, subscale, or time-specific analyses, and that the corresponding P-values should be interpreted as nominal and exploratory.

We described in Result sections in revised manuscript following

“No adjustment for multiplicity was performed for secondary, subscale, or time-specific analyses; therefore, the corresponding P-values should be interpreted as nominal and exploratory.” (P11, L167).

**Comment #2**

2- Exploratory Outcomes – Secondary endpoints are likely underpowered and should be explicitly described as exploratory rather than confirmatory.

**Response#2**

We agree that the secondary outcomes should be interpreted as exploratory rather than confirmatory. The study was powered only for the primary endpoint, and the secondary analyses were intended to generate supportive and hypothesis-generating evidence. We have therefore clarified throughout the manuscript that secondary efficacy and safety outcomes were analyzed and interpreted in an exploratory manner.

We described in Result sections following sentence:

“The secondary endpoints for efficacy and safety were analyzed in an exploratory manner.” (P11, L164)

**Comment #3**
3-Interaction Effects and Reporting Transparency — The manuscript includes treatment × time interaction terms in the mixed-effects models, which is appropriate for a longitudinal randomized design. However, the statistical decision process surrounding these interactions should be made explicit and align more closely with standard practice. Specifically, the authors should clarify whether the global interaction effects were formally tested and whether they were statistically significant. If interactions were significant, the results should be interpreted explicitly in terms of differential treatment effects over time, and main effects should not be separately tested or discussed. Conversely, if interaction effects were non-significant, the interaction terms should be removed and main effects reported instead. Clearly stating and consistently applying this stepwise interaction-testing framework would improve transparency, interpretability, and statistical rigor, particularly given the reliance on time-specific contrasts across multiple outcomes.

**Response#3**

Thank you for this helpful comment. In the present study, we adopted a standard mixed-effects model for repeated measures (MMRM) including fixed effects for treatment group, time (visit), and the treatment-by-time interaction, together with baseline adjustment and an unstructured covariance matrix. This specification is widely recommended for the analysis of longitudinal continuous outcomes in randomized clinical trials because it allows treatment effects to vary flexibly across visits and provides valid estimates under the missing-at-random assumption (Mallinckrodt et al., 2001; 2008).

Our primary objective was to estimate treatment effects at each post-baseline visit rather than to select a final model based on significance testing of interaction terms. Therefore, the treatment-by-time interaction was retained in the primary MMRM specification regardless of its statistical significance. Time-specific treatment effects were estimated directly from this prespecified model.

To improve transparency, we have revised the Methods section to describe the MMRM specification and interpretation strategy more explicitly.

Mallinckrodt CH, Clark WS, David SR.
*Accounting for dropout bias using mixed-effects models.*
J Biopharm Stat. 2001.

Mallinckrodt CH et al.
*Recommendations for the Primary Analysis of Continuous Endpoints in Longitudinal Clinical Trials.*
Drug Information Journal. 2008.

**Comment #4**
1- Bias and Subjectivity – The open-label design combined with reliance on subjective, self-reported measures may inflate observed treatment effects.

**Response#4**

We agree with the reviewer's comments. The therapeutic effects have the potential to be inflated. We considered this a limitation and added the following sentence “Furthermore, the use of self-report questionnaires in this open-label study may have led to an overestimation of the therapeutic effect” (P28, L434).

**Comment #5**
2- Missing Data / Wearables – Substantial missingness and baseline imbalances in wearable-derived sleep data limit interpretability and should be emphasized as exploratory.

**Response#5**

We agree. We have revised the manuscript to include following; “The Fitbit measurements were obtained as exploratory assessments, and interpretation requires caution due to missing data” (P28, L437).

**Comment #6**

3- Sample Size Justification — Indicate the statistical testing method which achieves 80% power.

**Response#6**

We agree that the statistical method used for the sample size calculation should be stated explicitly. The sample size was calculated based on a two-sample t-test for the between-group difference in the primary endpoint, assuming a mean difference of 6.0, a standard deviation of 6.73, a two-sided significance level of 0.05, and 80% power. We have clarified this in the Statistical Analysis section.

We added the following sentence:

“The sample size was designed based on a two-sample t-test to detect a 6.0 mean difference with a 6.73 standard deviation for the primary outcome, with a two-sided α level of 0.05 and 80% power between group.” (P11, L153)

**Response to the comments of reviewer #2**

**Comment #1**

1- The authors acknowledge that the trial was open-label and not blinded, which introduces a significant risk of placebo effect, especially for subjective psychological symptoms. Please expand on how this lack of blinding might specifically impact the interpretation of the secondary endpoints, such as "overall impressions," where the KSS group reported much higher satisfaction.

**Response#**1

We agree with the reviewer's comments. These results must be interpreted with due regard for the risks associated with open-label trials.

We have revised the manuscript:

“It is suggested that the amelioration of overall impression survey was due to the alleviation of menstrual distress symptoms from the premenstrual phase through during-menstruation, in addition to improvements in daily activity impairment levels. As an important note, these results were obtained as an exploratory open-label clinical study and should be interpreted with caution” (P27, L418).

**Comment #2**

2- This study enrolled 50 patients, which was the minimum calculated for 80% power. However, the efficacy analysis population dropped to 48 by the second cycle and 47 by the third. Please clarify if the MMRM analysis sufficiently accounts for these missing data points to maintain the intended statistical power, or if the study should be explicitly labeled as "underpowered" for secondary outcomes like WPAI, where the sample of workers was noted as being too small.

**Response#**2

Thank you for this comment. The sample size calculation incorporated an anticipated dropout rate of 20%, and the observed loss to follow-up was substantially smaller than expected (2 participants in total). Therefore, the number of participants available for the primary efficacy analysis remained within the assumptions used for the original sample size calculation.

The primary efficacy analysis was conducted using a mixed-effects model for repeated measures (MMRM), which uses all available repeated measurements without ad hoc imputation under a missing-at-random assumption. Given the small amount of missing data, we do not consider the primary endpoint analysis to have been materially affected.

Regarding WPAI, we agree that the effective sample size was limited because absenteeism, presenteeism, and work productivity loss can only be evaluated among employed participants. In this study, a total of 26 workers were enrolled, consisting of an observation group (n=12) and a KSS group (n=14) . The dropout rate is 20% over. Therefore, these analyses should be regarded as exploratory and interpreted with caution. We have clarified this point in the revised manuscript (P21, L322).

**Comment #3**

3- Authors mention that significant effects were not observed for absenteeism or work productivity loss because the number of workers was small. Please provide the exact number of employed participants in each group to provide context for these specific secondary outcomes.

**Response#**3

Thank you for this comment. We agree.

We have revised the manuscript:

“A total of 26 workers were enrolled in this study, consisting of an observation group (n=12) and a KSS group (n=14)” (P21, L322).

Others

We have corrected the typographical errors following.

#1 Tsumura & Co. → TSUMURA & CO

#2 MDQ^b^ → ^b^MDQ

#3 evaluation-related mental status and menstruation-related symptoms → daily symptom questionnaires, coldness and stiff shoulder

Fuminori Taniguchi MD., PhD.

Obstetrics and Gynecology, Tottori University Faculty of Medicine, Tottori, Japan

E-mail addresses: tani4327@tottori-u.ac.jp
