# Supplementary material for: A Kampo medicine, kamishoyosan, ameliorates menstruation-associated symptoms in patients with premenstrual syndrome: An exploratory open-label, randomized controlled trial
Source: PLoS One. 2026 Nov 6;21(11):e0362303. doi: 10.1371/journal.pone.0362303 (PMC13742958; doi:10.1371/journal.pone.0362303)
Supplement: Supplementary file 5 [file pone.0362303.s007.docx]

List of Point-by-Point Response to the Reviewer’s Comments

**1- The study remains open-label without a placebo control, which leaves the results highly susceptible to expectation and reporting bias, particularly for subjective outcomes.**

**Response**

We thank the reviewer for this important comment. We agree that the open-label design without placebo control may have introduced expectation and reporting bias, particularly for subjective outcomes. This issue has been acknowledged in the Limitations section. To further clarify this point, we revised the text as follows:

“First, the trial design was open-label and not blinded; thus, the potential for a placebo effect cannot be excluded. Futhermore, as several outcomes were assessed using patient-reported outcome measures, including symptom questionnaires and global impression ratings, the therapeutic effects observed in this open-label study may have been overestimated due to expectation and reporting bias.” (P29, L459-463)

**2- No adjustment for multiple comparisons has been implemented despite extensive testing across numerous endpoints, time points, and subscales, increasing the likelihood of false positive findings.**

**Response**

We thank the reviewer for this important comment. We agree that the large number of secondary, subscale, and time-specific analyses increases the risk of false-positive findings when multiplicity is not formally controlled.

Because this study was designed as an exploratory trial, no adjustment for multiplicity was prespecified for secondary, subscale, or time-specific analyses. To address the reviewer’s concern, we have revised the manuscript to clearly distinguish the primary endpoint analysis from exploratory analyses. Specifically, we clarified in the Statistical Analysis section that no multiplicity adjustment was performed for secondary, subscale, or time-specific analyses and that the corresponding p-values should be interpreted as nominal and exploratory.

In addition, we revised the Results, figure legends, Discussion, and Limitations sections to consistently report secondary findings as nominal and exploratory findings and to avoid confirmatory interpretations.

The modified descriptions are listed below;

1. Abstract (P3 L34, L35, L38)

2. The Results (P16 L233, P17 L237, L243, L250, P18, L258, P19, L264, P20 L270, 277, P20, L291, L299, L306, P21, L310, L316, L324, P22 L325, L332, L333, P23 L345)

3. Discussion (P25, L382, L385, L392, P25 L399, L405, L411, P26 L418, P27 L438, L440, L444, L450, P29 L457)

4. Limitation (P29 L461)

5. Conclusion (P30 L475)

**3- Baseline imbalances are acknowledged for some outcomes, but there is no consistent statistical adjustment applied to account for these differences.**

**Response**

We thank the reviewer for this important comment. We would like to clarify that all MMRM analyses for the primary endpoint and MDQ-related secondary endpoints included the corresponding baseline score as a covariate. Therefore, baseline differences were statistically adjusted for in these longitudinal analyses (P11, L160-162).

We acknowledge that baseline differences were observed for some exploratory outcomes that were analyzed using simple between-group comparisons rather than MMRM. Because these analyses were exploratory in nature, their results should be interpreted cautiously. To improve transparency, we revised the Results, figure legends, and Discussion sections to more clearly acknowledge baseline differences where relevant and to avoid overinterpretation of these exploratory findings.

Please also refer to our response to Comment #2 regarding the interpretation of exploratory analyses.

**4- The sample size is small relative to the complexity of the models used, raising concerns about model stability and reliability of estimates.**

**Response**

We thank the reviewer for this important comment. We agree that the sample size was relatively small, particularly in relation to the number of secondary and exploratory analyses performed. However, the sample size was prospectively determined based on the primary endpoint and was not intended to provide definitive evidence for all secondary, subscale, or time-specific analyses. Consistent with the exploratory nature of this study, we have revised the manuscript to clearly distinguish the primary endpoint analysis from exploratory analyses and to clarify that p-values from secondary analyses should be interpreted as nominal. In addition, we have strengthened the Limitations section to emphasize that the relatively small sample size may limit the precision of some exploratory findings and that these results should be interpreted with caution.

To further clarify this point, we revised the text as follows:

Second, the sample size was relatively small, which may have limited the precision and stability of some estimates, particularly for secondary and exploratory analyses.(P29, L465)

**5- The use of an unstructured covariance matrix in a small sample repeated-measures setting may be overparameterized and insufficiently justified.**

**Response**

We thank the reviewer for this important comment. We recognize the concern regarding the use of an unstructured covariance matrix in a relatively small study. An unstructured covariance matrix was prespecified because it makes minimal assumptions about the correlation pattern among repeated measurements and is a commonly used covariance structure in MMRM analyses. To clarify the rationale for this choice, we added the following sentence to the Statistical Analysis section:

“The covariance matrix structure was specified as unstructured because this approach places minimal assumptions on the correlation pattern among repeated measurements.”(P11, L159)

**6- Parametric tests are used for ordinal or bounded numeric rating scale outcomes without justification or sensitivity analyses to assess robustness.**

**Response**

We thank the reviewer for this important comment. We agree that several outcomes were measured using ordinal or bounded rating scales. However, these scales consisted of multiple ordered response categories, including 0–10 numerical rating scales and a 7-point global impression scale, and were analyzed as approximately continuous variables, consistent with common practice in clinical research and randomized clinical trials.

Parametric methods were selected because they allow estimation of mean differences between groups and are generally considered robust when applied to rating scales with multiple response categories. To clarify the rationale for this analytical approach, we have added the following statement to the Statistical Analysis section:

NRS outcomes were treated as approximately continuous variables for analysis, consistent with common practice in clinical research.(P12, L174)

**7- Missing data are not formally addressed beyond relying on the mixed model, and there is no assessment of the missing data mechanism or potential bias.**

**Response**

We thank the reviewer for this important comment. Missing outcome data were limited in this study. Two participants in the KSS group discontinued study participation because of restricted medication use and withdrawal of consent. The primary analysis was conducted using a mixed-effects model for repeated measures (MMRM), which incorporates all available longitudinal observations under the missing-at-random (MAR) assumption without ad hoc imputation. Given the small amount of missing data and the reasons for discontinuation, we believe that the risk of substantial bias due to missing data is limited.

To improve transparency, we have revised the Statistical Analysis section to clarify the assumption underlying the MMRM approach and added the following statement:

The primary analysis was conducted using MMRM, which uses all available observations under the missing-at-random assumption.(P11, L164)

**8- The analysis population is not clearly defined, leaving ambiguity about whether a true intention-to-treat approach was implemented.**

**Response**

We thank the reviewer for this important comment. We agree that the analysis population was not sufficiently described in the original manuscript. Efficacy analyses were conducted using the Full Analysis Set (FAS), which consisted of all randomized participants who had at least one post-baseline efficacy assessment. Missing values were not imputed, and all available data were analyzed using the prespecified statistical methods.

To improve clarity, we have revised the Statistical Analysis section to explicitly define the analysis population and clarify the use of the FAS.

The Full Analysis Set (FAS) consisted of all randomized participants who had available post-baseline efficacy data.(P12, L178)

**9- Exploratory secondary and subgroup findings are interpreted as supportive evidence without sufficient caution given the lack of multiplicity control.**

**Response**

We thank the reviewer for this important comment. We agree that secondary, subscale, and time-specific analyses should be interpreted with caution given the exploratory nature of this study and the absence of multiplicity adjustment.

To address this concern, we have substantially revised the manuscript to clearly distinguish the primary endpoint analysis from exploratory analyses. Specifically, we clarified in the Statistical Analysis section that no adjustment for multiplicity was performed for secondary, subscale, or time-specific analyses and that the corresponding p-values should be interpreted as nominal and exploratory (P11, L168 - P12, L170).

In addition, we revised the Results, figure legends, Discussion, and Conclusion sections to avoid confirmatory interpretations of exploratory findings. References to "significant" effects or "confirmed efficacy" were replaced with more cautious descriptions, and exploratory results are now consistently described as nominal findings that should be interpreted with caution.

We also added the following statement to the Limitations section:

“The study was exploratory, and no adjustment for multiplicity was performed for secondary, subscale, or time-specific analyses. Therefore, these findings should be interpreted cautiously.” (P29, L468)

Accordingly, the prespecified primary endpoint analysis and the exploratory secondary analyses are now clearly distinguished, and all secondary findings are reported as nominal and exploratory.

**10- Subgroup analyses, such as those involving work productivity outcomes, are based on very small sample sizes and are not adequately qualified as underpowered.**

**Response**

We thank the reviewer for this important comment. We agree that the WPAI analyses involving absenteeism, presenteeism, and work productivity loss were based on a relatively small number of working participants and should therefore be interpreted cautiously.

To address this concern, we revised the manuscript to explicitly report the sample sizes for these analyses. Specifically, absenteeism, presenteeism, and work productivity loss outcomes were evaluated only among working participants (Observation group, n = 12; KSS group, n = 14). (P22, L337) Given this limited sample size, these analyses may have been underpowered to detect differences in work productivity outcomes.

In contrast, activity impairment was assessed in all evaluable participants, including non-workers (Observation group, n = 25; KSS group, n = 23). Therefore, the activity impairment analysis was based on a larger sample size than the work-related WPAI outcomes. (P22, L339)

We also revised the Results, Figure 6 legend, and Discussion section to clarify the sample sizes for each WPAI domain and to emphasize that these results should be regarded as exploratory findings.

Fuminori Taniguchi MD., PhD.

Obstetrics and Gynecology, Tottori University Faculty of Medicine, Tottori, Japan

E-mail addresses: tani4327@tottori-u.ac.jp
