# Supplementary material for: A Kampo medicine, kamishoyosan, ameliorates menstruation-associated symptoms in patients with premenstrual syndrome: An exploratory open-label, randomized controlled trial
Source: PLoS One. 2026 Nov 6;21(11):e0362303. doi: 10.1371/journal.pone.0362303 (PMC13742958; doi:10.1371/journal.pone.0362303)
Supplement: Supplementary file 6 [file pone.0362303.s008.docx]

List of Point-by-Point Response to the Reviewer’s Comments

**Response to Reviewer #1**

We thank the reviewer for the additional statistical comments and for the careful consideration of our revised manuscript.

We carefully considered the suggested additional analyses. This study was prospectively designed as a small exploratory randomized trial with a prespecified primary endpoint and analysis strategy. The primary endpoint was analyzed using the prespecified baseline-adjusted mixed-effects model for repeated measures (MMRM), whereas the other outcomes were intended to be exploratory.

We have therefore retained the prespecified analyses. Instead, we have further strengthened the manuscript to make clear that these secondary analyses are exploratory, that their p-values are nominal and unadjusted for multiplicity, and that the findings should be regarded as hypothesis-generating rather than confirmatory. We have also further qualified the interpretation of outcomes for which baseline imbalances or substantial missing data were observed.

Our detailed responses are provided below.

Reviewer #1:

1- Adjust exploratory outcomes with baseline imbalances using an appropriate longitudinal or covariate-adjusted approach rather than relying solely on unadjusted between-group comparisons.

**Response**

We thank the reviewer for this suggestion.

For the prespecified primary endpoint and the MDQ-related longitudinal analyses, baseline values were already included as covariates in the MMRM. Thus, the primary efficacy analysis and MDQ-related longitudinal analyses appropriately account for baseline measurements.

For the other secondary outcomes, the analyses were prespecified as exploratory and were not designed as confirmatory treatment-effect analyses. Importantly, we do not believe that selecting covariate adjustment specifically for outcomes showing observed baseline imbalances after randomization would be an appropriate basis for modifying the analysis strategy after completion of the trial. In a randomized study, baseline differences can occur by chance, particularly with a modest sample size, and choosing an adjusted analysis according to the observed degree of baseline imbalance would constitute a post hoc, data-dependent analytical decision.

We have therefore retained the prespecified exploratory analyses rather than introducing additional outcome-specific adjusted models. To address the reviewer’s concern, however, we have further clarified in the manuscript that these comparisons are exploratory and that findings for outcomes with baseline imbalances should be interpreted with particular caution.

No additional statistical analysis was performed in response to this comment.

2- Assess whether the observed differences in diary and Fitbit outcomes persist after accounting for the reported baseline differences between treatment groups.

**Response**

We appreciate this comment and agree that the reported baseline differences are relevant to interpretation.

The diary and Fitbit outcomes were secondary exploratory assessments and were not intended to provide confirmatory evidence of treatment efficacy. Baseline-adjusted analyses of these outcomes were not prespecified. We therefore decided not to introduce new covariate-adjusted or longitudinal models after examination of the observed baseline differences.

This consideration is particularly important for the Fitbit outcomes. As already reported in the manuscript, these measurements contained substantial missing data because of difficulties with device attachment, and baseline differences were also observed for several Fitbit variables. Under these circumstances, a newly introduced adjusted model in this small sample would itself depend on additional modeling assumptions and would not convert these exploratory measurements into confirmatory evidence.

We have instead further qualified the interpretation of both diary and Fitbit findings. The manuscript now explicitly emphasizes that findings affected by baseline imbalance should be regarded as hypothesis-generating and should not be interpreted as demonstrating treatment effects independent of the observed baseline differences. The Fitbit findings are additionally described as exploratory and limited by substantial missing data.

No additional statistical analysis was performed.

3- Reanalyze the multiple visit-specific t-tests used for repeated exploratory outcomes with longitudinal models that account for within-subject correlation over time.

**Response**

We thank the reviewer for this important suggestion. We agree that a longitudinal model accounting for within-subject correlation would be appropriate if the objective were to estimate an overall treatment-by-time effect or to make joint longitudinal inferences across visits.

However, the visit-specific analyses in the present study had a different, explicitly exploratory objective, namely, to describe between-group differences at individual assessment times. These analyses were conducted in accordance with the prespecified exploratory analysis strategy and were not intended to provide confirmatory inference regarding an overall longitudinal treatment effect.

We therefore considered that replacing these analyses with a newly specified longitudinal model at this stage would change both the analytical approach and, to some extent, the inferential target after examination of the study data. Accordingly, we have retained the prespecified exploratory analyses rather than introducing a post hoc longitudinal model.

We nevertheless acknowledge the reviewer’s concern that repeated visit-specific testing increases the opportunity for chance findings. We have therefore further clarified throughout the manuscript that the corresponding p-values are nominal and unadjusted for multiplicity, and that these findings should be interpreted as hypothesis-generating rather than confirmatory.

No additional statistical analysis was performed in response to this comment.

4- Demonstrate the robustness of exploratory findings to the large number of statistical tests performed, particularly for symptom diary, WPAI, Fitbit, and subscale analyses.

**Response**

We agree with the reviewer that the large number of exploratory comparisons substantially increases the probability of nominally significant findings occurring by chance.

However, no multiplicity-adjustment strategy or confirmatory family of hypotheses was prespecified for these secondary endpoints. A post hoc adjustment would require retrospective choices regarding which endpoints and time points constitute a family of hypotheses and which multiplicity procedure should be applied. We believe that making these choices after completion of the trial would introduce additional analytical flexibility rather than provide a prespecified assessment of robustness.

Importantly, the study has a single prespecified primary endpoint, and inference regarding this endpoint is separate from the exploratory secondary analyses. We make no claim that the nominal secondary findings are robust to multiplicity adjustment or constitute confirmatory evidence.

We have therefore not performed post hoc multiplicity-adjusted analyses. Instead, we have further strengthened the manuscript to state explicitly that the symptom diary, WPAI, Fitbit, MDQ subscale, and other secondary findings are based on nominal testing without multiplicity adjustment and should be interpreted solely as hypothesis-generating observations requiring confirmation in future studies.

No additional statistical analysis was performed.

5- Further qualify the interpretation of statistically significant exploratory findings by emphasizing that they arise from nominal testing without multiplicity adjustment and should be considered hypothesis-generating rather than confirmatory.

**Response**

We agree with the reviewer and have revised the manuscript accordingly.

Throughout the manuscript, we have further qualified the interpretation of secondary findings. We now explicitly state that p-values for secondary, subscale, and time-specific analyses are nominal and were not adjusted for multiplicity, and that these findings should be considered hypothesis-generating rather than confirmatory.

We have also revised the Abstract, Statistical Analysis section, Results, Discussion, Limitations, and Conclusion to avoid language suggesting definitive efficacy on the basis of exploratory outcomes.

We thank the reviewer for this suggestion, which has helped us further clarify the intended interpretation of these analyses.

The proposed revisions are summarized below.

Abstract:

These exploratory findings suggest that KSS may improve menstrual-related symptoms in PMS.(P3, L39)

Statistical Analysis section:

No adjustment for multiplicity was performed for secondary, subscale, or time-specific analyses; therefore, the corresponding p-values are nominal and exploratory and should be interpreted as hypothesis-generating rather than confirmatory.(P12, L170)

Results:

However, baseline imbalances in anxiety and emotional instability were observed before treatment. Accordingly, the corresponding exploratory between-group findings should be interpreted cautiously and should not be regarded as evidence of treatment effects independent of these baseline differences.(P19, L280)

Given the substantial missing data and the baseline imbalances in several Fitbit measures, these results should be considered descriptive and hypothesis-generating and should not be interpreted as confirmatory evidence of a treatment effect.(P23, L355)

Discussion:

Overall, the findings from the secondary analyses should be interpreted with caution, as these analyses involved numerous outcomes and assessment times without adjustment for multiplicity. Individual nominally significant findings should therefore be regarded as hypothesis-generating rather than as confirmatory evidence of treatment efficacy. (P28, L443)

Limitations:

Accordingly, these nominal findings should be regarded as hypothesis-generating rather than confirmatory. Chance baseline imbalances were also present for some exploratory outcomes, and these may have influenced the corresponding unadjusted comparisons. (P29, L455)

Conclusion:

The findings of this exploratory randomized trial suggest that KSS may improve menstrual-related symptoms in PMS. Additionally, no serious adverse events were reported during study. Taken together, these findings suggest the potential clinical utility of KSS for PMS. (P29, L462)
